# Exploring multimorbidity profiles in middle-aged inpatients: a network-based comparative study of China and the United Kingdom

**DOI:** 10.1186/s12916-023-03204-y

**Published:** 2023-12-13

**Authors:** Yining Bao, Pengyi Lu, Mengjie Wang, Xueli Zhang, Aowei Song, Xiaoyun Gu, Ting Ma, Shu Su, Lin Wang, Xianwen Shang, Zhuoting Zhu, Yuhang Zhai, Mingguang He, Zengbin Li, Hanting Liu, Christopher K. Fairley, Jiangcun Yang, Lei Zhang

**Affiliations:** 1 China-Australia Joint Research Center for Infectious Diseases, School of Public Health, Xi’an Jiaotong University Health Science Center, Xi’an, 710061 Shaanxi China; 2 Melbourne Sexual Health Centre, Alfred Health, Melbourne, VIC Australia; 3 Central Clinical School, Faculty of Medicine, Nursing and Health Sciences, Monash University, Melbourne, VIC Australia; 4 Medical Research Institute, Guangdong Provincial People’s Hospital (Guangdong Academy of Medical Sciences), Southern Medical University, Guangzhou, China; 5 Guangdong Eye Institute, Department of Ophthalmology, Guangdong Provincial People’s Hospital (Guangdong Academy of Medical Sciences), Southern Medical University, Guangzhou, China; 6 Department of Transfusion Medicine, Shaanxi Provincial People’s Hospital, 256 Youyi West Road, Xi’an, 710068 China; 7 Department of Information Technological, Shaanxi Health Information Center, Xi’an, China; 8 Clinical Research Management Office, The Second Affiliated Hospital of ChongQing Medical University, Chongqing, China; 9 AIM Lab, Faculty of IT, Monash University, Melbourne, VIC Australia; 10 College of Intelligent Systems Science and Engineering, Harbin Engineering University, Harbin, 150001 China; 11 Royal Melbourne Hospital, University of Melbourne, Melbourne, VIC Australia; 12 Centre for Eye Research Australia, Royal Victorian Eye and Ear Hospital, Melbourne, VIC Australia; 13 Division of Ophthalmology, Department of Surgery, University of Melbourne, Melbourne, VIC Australia; 14 Gies College of Business, University of Illinois Urbana-Champaign, Champaign, IL USA

**Keywords:** Multimorbidity, Comorbidity, Network analysis, Middle-aged, Inpatients, China, United Kingdom

## Abstract

**Background:**

Multimorbidity is better prevented in younger ages than in older ages. This study aims to identify the differences in comorbidity patterns in middle-aged inpatients from China and the United Kingdom (UK).

**Methods:**

We utilized 184,133 and 180,497 baseline hospitalization records in middle-aged populations (40–59 years) from Shaanxi, China, and UK Biobank. Logistic regression was used to calculate odds ratios and *P* values for 43,110 unique comorbidity patterns in Chinese inpatients and 21,026 unique comorbidity patterns in UK inpatients. We included the statistically significant (*P* values adjusted by Bonferroni correction) and common comorbidity patterns (the pattern with prevalence > 1/10,000 in each dataset) and employed network analysis to construct multimorbidity networks and compare feature differences in multimorbidity networks for Chinese and UK inpatients, respectively. We defined hub diseases as diseases having the top 10 highest number of unique comorbidity patterns in the multimorbidity network.

**Results:**

We reported that 57.12% of Chinese inpatients had multimorbidity, substantially higher than 30.39% of UK inpatients. The complete multimorbidity network for Chinese inpatients consisted of 1367 comorbidities of 341 diseases and was 2.93 × more complex than that of 467 comorbidities of 215 diseases in the UK. In males, the complexity of the multimorbidity network in China was 2.69 × more than their UK counterparts, while the ratio was 2.63 × in females. Comorbidities associated with hub diseases represented 68.26% of comorbidity frequencies in the complete multimorbidity network in Chinese inpatients and 55.61% in UK inpatients. Essential hypertension, dyslipidemia, type 2 diabetes mellitus, and gastritis and duodenitis were the hub diseases in both populations. The Chinese inpatients consistently demonstrated a higher frequency of comorbidities related to circulatory and endocrine/nutritional/metabolic diseases. In the UK, aside from these comorbidities, comorbidities related to digestive and genitourinary diseases were also prevalent, particularly the latter among female inpatients.

**Conclusions:**

Chinese inpatients exhibit higher multimorbidity prevalence and more complex networks compared to their UK counterparts. Multimorbidity with circulatory and endocrine/nutritional/metabolic diseases among both Chinese and UK inpatients necessitates tailored surveillance, prevention, and intervention approaches. Targeted interventions for digestive and genitourinary diseases are warranted for the UK.

**Supplementary Information:**

The online version contains supplementary material available at 10.1186/s12916-023-03204-y.

## Background

Multimorbidity, affecting 33.1% of adults globally in 2021, is a significant and rapidly growing public health concern worldwide [1, 2], with rising prevalence in England [3], Southern Europe [4], and China [5]. Multimorbidity leads to polypharmacy and adverse drug events [1, 6]. Furthermore, managing and treating multimorbidity puts a substantial strain on healthcare resources and increases health expenditure [1, 6, 7]. The World Health Organization recognizes the magnitude of the global multimorbidity challenge and has prioritized the goal of reducing the burden of multimorbidity [7].

Preventing multimorbidity in younger age is a more effective and economically viable strategy than treating it in older age. Elderly individuals with multimorbidity experience worse clinical outcomes, lower quality of life, reduced functionality, and increased hospitalization and mortality compared to single-disease counterparts [1, 6]. The likelihood of poor health status and limited physical capacity in the elderly increases by 3–9 times with multimorbidity [4]. Interventions in the early stages of life can reduce the subsequent disease burden of multimorbidity in later life stages. Middle age may be the perfect stage of life for prevention and early intervention, as severe chronic ailments are either absent or only in their early stages of development. Prevention and treatment for multimorbidity are much more efficient in this stage of life. Comprehending multimorbidity patterns in middle-aged populations has crucial implications for effective interventions that may reduce the subsequent disease and economic burden of multimorbidity [8, 9].

The current research on multimorbidity is riddled with significant knowledge gaps, despite numerous efforts. Existing studies are largely limited to developed countries, with only limited research from developing countries [10–13]. Besides, the current research has focused on older adults [14–16] but not the younger middle-aged population. More importantly, most existing studies have focused on a small group of chronic diseases, with limited exploration of multimorbidity on a wider spectrum of diseases, except only a handful of studies in Southwest China [17], Denmark [11], America [12], and Austria [13]. To date, no studies comprehensively compared comorbidity patterns at a population level between developed and developing countries.

Complex disease interactions significantly influence diagnosis and treatment and pivotal inpatient care. Untangling these less-understood relationships is crucial [13, 18]. Our study employed network analysis, an innovative tool revealing intricate interconnections among diseases [10–13]. We used this approach to construct multimorbidity networks in China and the UK, identifying hub diseases with numerous comorbidity patterns. This innovation enhances our understanding of disease interactions and enables the development of targeted surveillance and prevention strategies, benefiting clinical practice.

Our study aims to compare the comorbidity patterns among inpatients in China and the United Kingdom (UK) to enhance the understanding of the differences in disease profiles in these countries. We facilitate the comparison based on two sizable population datasets in middle-aged populations from Northwest China and the UK-Biobank. The diagnosis of diseases is according to the International Statistical Classification of Diseases and Related Health Problems 10th revision (ICD-10) [19]. We included 14 systematic chapters on diseases and used sophisticated network analysis to develop multimorbidity networks, compare comorbidity patterns, and identify hub diseases between China and the UK [13]. Our findings are pivotal in global healthcare by highlighting disease disparities across countries with different economic development statuses. Our study will inform decision-makers to understand regional health challenges and endorse targeted strategies for multimorbidity prevention and treatment. Our findings will also guide global health institutes and governments, aiding in the formulation of more effective multimorbidity prevention strategies, thereby reducing both disease and economic burden caused by multimorbidity.

## Methods

### Study participants

Our study drew on data from two population studies from China and the UK. Both studies utilized the same disease diagnosis measure (ICD-10) [19]. The Chinese population initially recruited 678,666 participants in Shaanxi province, China (1998–2018), with 1,058,398 hospitalization records from the Centralized Hospital Medical Records (CHMRs) system [20]. The UK-Biobank population initially recruited 502,414 participants from the National Health Service (2006–2010), with 2,372,119 hospitalization records linked to the Hospital Episode Statistics (HES) [21]. The baseline was the date of the first record for each participant. ICD-10 codes were grouped into 22 chapters (Additional File 1: Table S1) [19]. Recruitment procedures and other details are documented elsewhere [20, 22] and presented in Additional File 2: Figure S1-S2.

### Inclusion and exclusion criteria

We employed consistent inclusion criteria for both datasets, requiring (1) baseline records of each inpatient, (2) health records with ICD-10 codes, and (3) records with ages ranging from 40 to 59 years (middle-aged). The exclusion criteria were as follows: health records only with ICD-10 codes from chapters 15–22, as these correspond to congenital diseases, injury, poisoning, pregnancy, childbirth and the puerperium, and others. Only records with ICD-10 codes from chapters 1–14 were included [10] (Additional File 2: Figure S1-S2).

### Study population and subpopulations

Inclusion and exclusion criteria yielded 184,133 Chinese hospitalization records and 180,497 UK hospitalization records. We further stratified our dataset by sex, examining comorbidities among males (China: 103,334 inpatients, UK: 79,652 inpatients) and females (China: 80,799 inpatients, UK: 100,845 inpatients) (Additional File 1: Table S2).

### Study indicators

Disease categories were identified using 3-character codes of the ICD-10 system. In Chinese inpatients, 929 diseases (854 in males, 819 in females) were recorded, and in UK inpatients, 834 diseases (724 in males, 736 in females) were recorded (Additional File 3).

### Definition of comorbidity and multimorbidity

We defined “comorbidity” as the coexistence of disorders in addition to a primary disease. The “comorbidity pattern” refers to the presence of exactly two diseases, whereas multimorbidity indicates the presence of two or more diseases in the same individual [1].

### Construction of multimorbidity network

The basic elements of the networks are nodes (diseases) and edges (coexistence of disease) that connect nodes within the networks. In the initial analysis, 43,110 unique comorbidity patterns emerged for the Chinese middle-aged inpatients (30,209 among males, 26,554 among females), and in the UK, 21,026 unique comorbidity patterns were found (12,887 among males, 13,197 among females). For disease associations, logistic regression models calculated odds ratios (ORs) for each comorbidity pattern (edge). Edges were selected based on criteria: (1) OR > 1; (2) *P*-value < 0.05/*N* (*N* = the number of patterns satisfying OR > 1, Bonferroni correction applied [23]); (3) patterns with prevalence > 1/10,000 in each dataset were included, while patterns with the lower prevalence were excluded (Additional File 2: Figure S3-S6).

### Hub diseases and associated multimorbidity network

The overall disease network encompassed all comorbidity patterns that satisfied the specified criteria for the entire study population (Additional File 2: Figure S3, S5). The same disease network was constructed in males and females to obtain sex-specific disease networks (Additional File 2: Figure S4, S6). Employing the degree network metric [24], we identified the top ten diseases with the most connections within the network, defining them as Hub diseases. If two diseases displayed the same degree, the diseases with a higher prevalence would be given the preference. The networks formed by comorbidities linked to the Hub diseases were defined as Hub diseases’ associated networks.

### Multimorbidity network metric

We obtained seven main metrics for each node: degree [24], maximal clique centrality (MCC) [25], closeness centrality (Clo_Cen) [24], clustering coefficient (Clu_Coe) [24], betweenness centrality (Bet_Cen) [24], pageranks [26], and eigencentrality [24] (the explanation of network metrics can be seen in Additional File 1: Additional Text). Node size was determined by degree, while edge thickness varied proportionally to OR values. Nodes were color-coded based on ICD-10 chapters. Additional File 3 provided detailed node metrics and OR values of edges.

### Statistical analysis

The proportion of each disease and comorbidity pattern was calculated. Age was measured by mean ± standard deviation (mean ± SD). Analyses were conducted using R 4.1.0 and Python 2020.1.3, and Cytoscape and Gephi were utilized for network diagram and metric analysis. Microsoft Excel and GraphPad Prism 9.3.1 were used to create stacked bar charts, line charts, and violin plots.

## Results

### Sociodemographic characteristics of the Chinese and UK inpatients

In the Chinese inpatients, the mean age of the overall, male, and female inpatients was 48.45 (SD ± 5.29), 48.27 (SD ± 5.31), and 48.67 (SD ± 5.27), respectively, and the proportion of females was 43.88%. In comparison, the UK inpatients were older with the corresponding ages of 51.53 (SD ± 5.27), 51.82 (SD ± 5.26), and 51.30 (SD ± 5.26) years and had a higher proportion of females (55.87%) (Additional File 1: Table S2).

### Disease profile in Chinese and UK inpatients

**Fig. 1:**
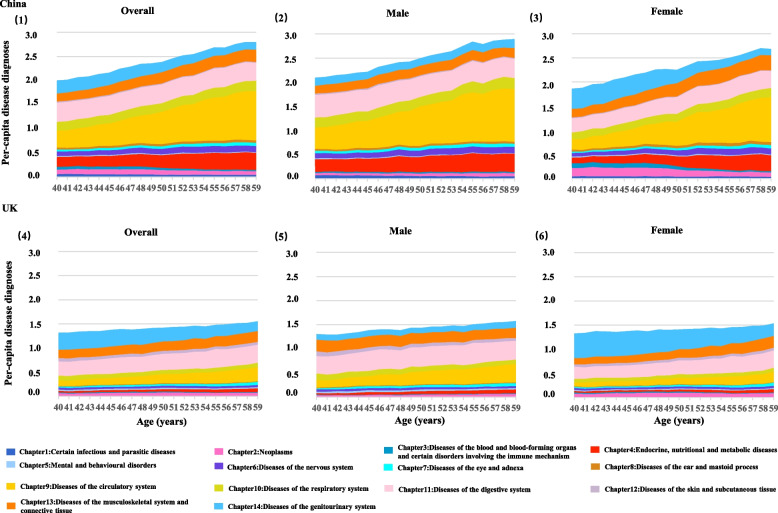
Stacked bar chart of per-capita disease diagnoses according to ICD-10 (chapters 1–14) among the Chinese and UK inpatients. The *x*-axis represents age (from 40 to 59 years, unit: years), and the *y*-axis represents the per-capita disease diagnoses. The color of each bar represents disease systematic chapters

The per-capita disease diagnoses increased with age in both Chinese and UK inpatients (Fig. 1), but the counts were consistently higher in Chinese inpatients than in UK inpatients across all ages. The overall per-capita disease diagnoses in the Chinese inpatients were 2.35/person, which was higher than that of the UK counterparts (1.44/person). In Chinese inpatients, circulatory diseases dominated (0.62/person), followed by digestive (0.39/person) and endocrine/nutritional/metabolic diseases (0.27/person). In comparison, in UK inpatients, digestive diseases dominated (0.34/person), followed by genitourinary (0.27/person) and circulatory diseases (0.21/person) (Additional File 1: Table S3).

The disease profiles were similar when stratified by sex in China. In the UK, musculoskeletal diseases were among the top three most common disease categories for both sexes (Additional File 1: Table S3).

### Multimorbidity profile in Chinese and UK inpatients

**Fig. 2:**
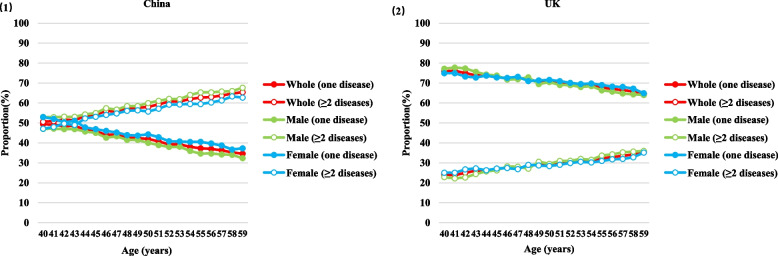
The proportion of single disease and multimorbidity, stratified by overall, male, and female inpatients in China and the UK

We found that 57.12% of Chinese inpatients were diagnosed with multimorbidity (≥ 2 diseases), while in the UK, it was much lower (30.39%) (Table 1). Notably, among the Chinese inpatients, the proportion of multimorbidity was higher than the proportion of a single disease in all age groups, whereas the opposite was observed in the UK inpatients (Fig. 2). Notably, the proportion of multimorbidity in both males and females followed a similar trend in both inpatient groups, although males tended to have a higher multimorbidity proportion than females.

**Table 1:** The number of health records, disease and multimorbidity diagnoses, and characteristics of complete multimorbidity network and hub diseases’ associated network in the Chinese and UK inpatients

	**Chinese inpatients**			**UK inpatients**		
	Overall	Male	Female	Overall	Male	Female
Number of health records	184,133	103,334	80,799	180,497	79,652	100,845
Number of diseases and diagnoses	341/440,702	320/237,746	297/169,760	215/221,470	215/101,683	187/119,214
Number of inpatients with a single disease (% of the total number of inpatients)	78,963 (42.88%)	42,871 (41.49%)	36,092 (44.67%)	125,648 (69.61%)	54,840 (68.85%)	70,808 (70.21%)
Number of inpatients with multimorbidity (≧ 2 conditions) (% of the total number of inpatients)	105,170 (57.12%)	60,463 (58.51%)	44,707 (55.33%)	54,849 (30.39%)	25,812 (31.15%)	30,037 (29.79%)
Number of nodes and edges in the complete multimorbidity network	341 1367	320 1179	297 990	215 467	215 438	187 377
Number of nodes and edges in hub diseases’ associated network (% of the complete multimorbidity network)	193 (56.60%), 483 (35.33%)	173 (54.06%) 410 (34.78%)	160 (53.87%) 347 (35.05%)	95 (44.19%) 176 (37.69%)	73 (33.95%) 154 (35.16%)	79 (42.25%) 141 (37.40%)
Frequency of comorbidity patterns in the complete multimorbidity network	287,195	174,985	99,086	50,375	24,180	25,508
Frequency of comorbidity patterns in hub diseases’ associated network (% of the complete multimorbidity network)	196,064 (68.26%)	128,025 (73.16%)	64,403 (65.00%)	28,014 (55.61%)	13,094 (54.15%)	14,034 (55.02%)

### Multimorbidity networks in overall Chinese and UK inpatients

**Fig. 3:**
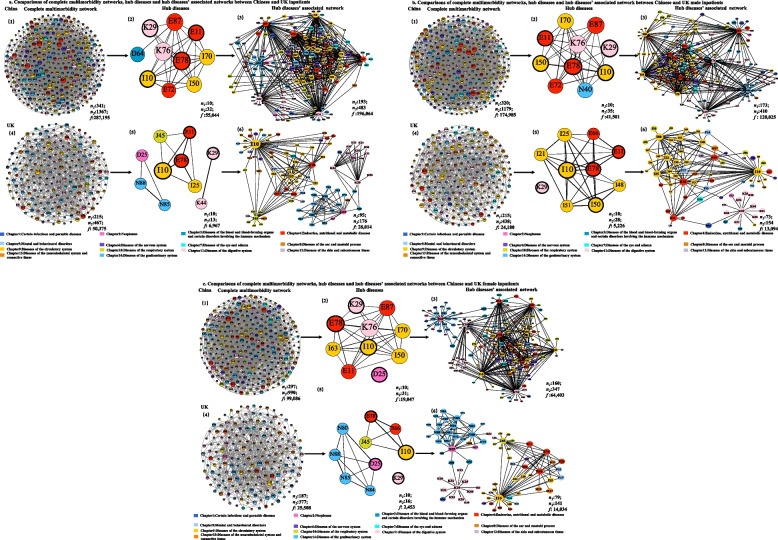
**a** Comparisons of complete multimorbidity networks, hub diseases, and hub diseases’ associated network between Chinese and UK inpatients. **b** Comparisons of complete multimorbidity networks, hub diseases, and hub diseases’ associated network between Chinese and UK male inpatients. **c** Comparisons of complete multimorbidity networks, hub diseases, and hub diseases’ associated network between Chinese and UK female inpatients. We presented the diseases in ICD-10 codes. Different colors denote different disease systematic chapters in the figure legend. The hub diseases between the Chinese and UK inpatients were highlighted in bold circles. *n*_*1*_: The number of nodes in the corresponding networks. *n*_*2*_: The number of edges in the corresponding networks. *f*: The total frequency of comorbidity patterns in the corresponding networks. The color of node was marked by according to disease systematic chapters. Disease name with ICD-10 codes (listed by alphabetical order): D25: Leiomyoma of uterus, D64: Other anemias, E11: Type 2 diabetes mellitus, E66: Obesity, E72: Other disorders of amino-acid metabolism, E78: Dyslipidemia, E87: Other disorders of fluid, electrolyte and acid–base balance, I10: Essential hypertension, I21: Acute myocardial infarction, I25: Chronic ischemic heart disease, I48: Atrial fibrillation and flutter, I50: Heart failure, I51: Complications and ill-defined descriptions of heart disease, I63: Cerebral infarction, I70: Atherosclerosis, J45: Asthma, K29: Gastritis and duodenitis, K44: Diaphragmatic hernia, K76: Other diseases of liver, N40: Hyperplasia of prostate, N80: Endometriosis, N84: Polyp of female genital tract, N85: Other noninflammatory disorders of cervix uteri, N88: Other noninflammatory disorders of uterus, except cervix

The *complete multimorbidity network* in Chinese inpatients was almost three times more complex than in UK inpatients (Fig. 3a). The Chinese multimorbidity network consisted of 341 diseases (nodes), which contributed to 1367 pairs of comorbidity patterns (edges), whereas the UK multimorbidity network consisted of 215 diseases and 467 pairs of comorbidity patterns (Additional File 1: Table S4-S6, Additional File 3). In Chinese inpatients, circulatory, endocrine/nutritional/metabolic, and digestive diseases-related comorbidities were most prevalent, while in UK, the most prevalent were circulatory, digestive, and genitourinary-related comorbidities (Additional File 2: Figure S7.1–7.2). Among Chinese inpatients, the most common comorbidity was essential hypertension/cerebral infarction comorbidity (I10 + I63, 6596/287,195), whereas duodenitis/diaphragmatic hernia comorbidity (K29 + K44, 1524/50,375) was the most common comorbidity in UK inpatients (Additional File 1: Table S7).

**Fig. 4:**
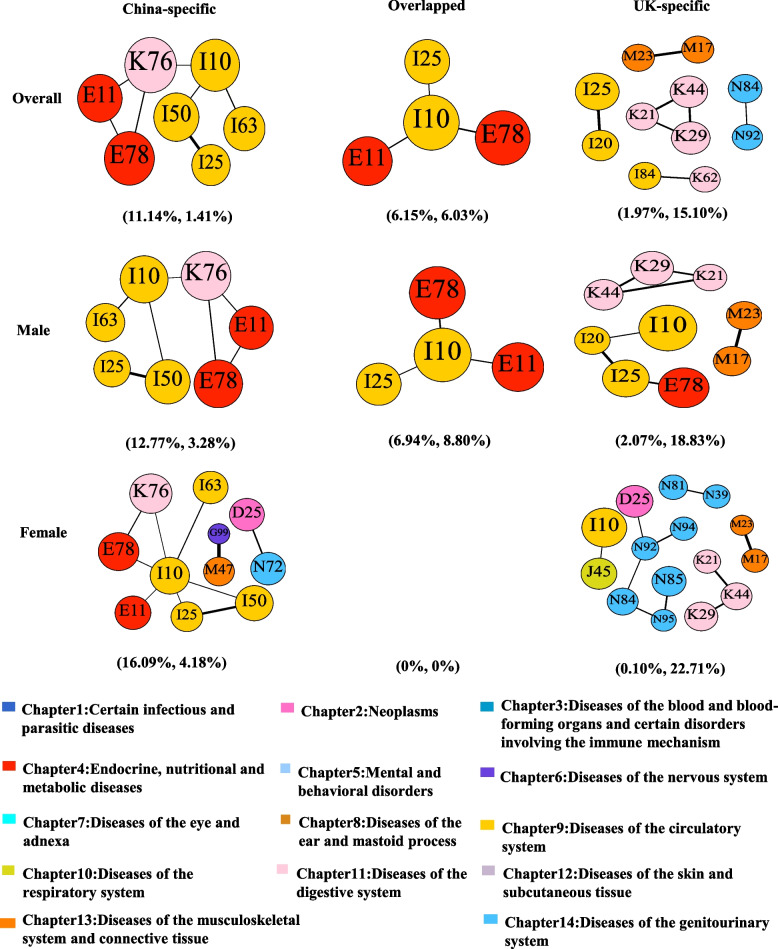
Overlapped and country-specific multimorbidity network patterns constructed based on the top 10 most prevalent comorbidities in overall, male, and female inpatients. In the brackets, the first number indicates ratio of the total frequency of comorbidity patterns in China-specific network and the total frequency of comorbidity patterns in the Chinese complete multimorbidity network, whereas the second number represents the corresponding ratio in the UK. The color of node was marked according to disease systematic chapters. We listed the diseases name with corresponding ICD-10 codes: D25: Leiomyoma of uterus, E11: Type 2 diabetes mellitus, E78: Dyslipidemia, G99: Other disorders of nervous system in diseases classified elsewhere, I10: Essential hypertension, I20: Angina pectoris, I25: Chronic ischemic heart disease, I50: Heart failure, I63: Cerebral infarction, I84: Hemorrhoids, J45: Asthma, K21: Gastroesophageal reflux disease, K29: Gastritis and duodenitis, K44: Diaphragmatic hernia, K62: Other diseases of anus and rectum, K76: Other diseases of liver, M17: Gonarthrosis [arthrosis of knee], M23: Internal derangement of knee, M47: Spondylosis, N39: Other disorders of urinary system, N72: Inflammatory disease of cervix uteri, N81: Female genital prolapse, N84: Polyp of female genital tract, N85: Other noninflammatory disorders of uterus, except cervix, N92: Excessive, frequent and irregular menstruation, N94: Pain and other conditions associated with female genital organs and menstrual cycle, N95: Menopausal and other perimenopausal disorders

The most common comorbidities shared between the Chinese and UK inpatients were all essential hypertension (I10)-related comorbidities (with dyslipidemia, E78; type 2 diabetes mellitus, E11; chronic ischemic heart disease, I25). Several comorbidities were very specific among Chinese inpatients. These included essential hypertension (I10)-related comorbidities (with cerebral infarction, I63; heart failure, I50), liver diseases (K76)-related comorbidities (with essential hypertension; I10, dyslipidemia, E78; type 2 diabetes mellitus, E11), and other heart-related (I25 + I50) and endocrinological comorbidities (E11 + E78). Comparatively, UK-specific comorbidities included gastritis/duodenitis (K29)-related comorbidities (with a diaphragmatic hernia, K44; gastroesophageal reflux disease, K21), musculoskeletal and connective tissue-related comorbidities (M17 + M23), and digestive-related (K21 + K44), heart-related (I20 + I25), and genitourinary comorbidities (N84 + N92) (Fig. 4 and Additional File 1: Table S7).

We identified ten hub diseases each from the Chinese and UK inpatients. Dyslipidemia (E78), type 2 diabetes (E11), essential hypertension (I10), and gastritis and duodenitis (K29) were overlapped hub diseases from the two populations. In Chinese inpatients, anemias (D64), disorders of fluid, electrolyte, acid–base balance (E87), amino-acid metabolism (E72), atherosclerosis (I70), heart failure (I50), and liver diseases (K76) were specific hub diseases (Additional File 1: Table S8). Comorbidities covered by its *hub diseases’ associated network* represented 35.33% (483/1367) of unique comorbidity patterns and 68.26% (196,046/287,195) of frequency of comorbidities in the complete network. In UK inpatients, leiomyoma of the uterus (D25), chronic ischemic heart disease (I25), asthma (J45), noninflammatory disorders of cervix uteri (N88), and uterus, except cervix (N85), were specific hub diseases. Comorbidities covered by its *hub diseases’ associated network* represented 37.69% (176/467) of unique comorbidity patterns and 55.61% (28,014/50,375) of frequency of comorbidities in the complete network (Fig. 3a, Table 1, Additional File 1: Table S4).

### Multimorbidity networks in Chinese and UK male inpatients

The *complete multimorbidity network* in Chinese male inpatients was 2.69 times more complex than in UK male inpatients (Fig. 3b, Additional File 1: Table S4, Additional File 3, China: 320 diseases/1,179 comorbidities; UK: 215 diseases/438 comorbidities). In Chinese inpatients, circulatory, endocrine/nutritional/metabolic, and digestive diseases-related comorbidities were most prevalent, while in UK, the most prevalent were circulatory, digestive, and musculoskeletal diseases-related comorbidities (Additional File 2: Figure S7.1–7.2). Among Chinese male inpatients, the most common comorbidity was essential hypertension/cerebral infarction comorbidity (I10 + I63, 4598/174,985), whereas dyslipidemia/essential hypertension comorbidity (E78 + I10, 900/24,180) was the most common in UK male inpatients (Additional File 1: Table S7).

Notably, the three most common comorbidities shared between the Chinese and UK male inpatients were identical to those of the overall inpatients, and so were the China-specific comorbidities. On the contrary, common UK-specific comorbidities in males included gastritis/duodenitis (K29)-related comorbidities (with a diaphragmatic hernia, K44; gastroesophageal reflux disease, K21), other digestive-related comorbidity (K21 + K44), chronic ischemic heart disease (I25)-related comorbidities (with dyslipidemia, E78; angina pectoris, I20), gonarthrosis/internal derangement of knee (M17 + M23), and angina pectoris/essential hypertension (I20 + I10) (Fig. 4 and Additional File 1: Table S7).

For hub diseases, dyslipidemia (E78), type 2 diabetes (E11), essential hypertension (I10), gastritis and duodenitis (K29), and heart failure (I50) overlapped in both Chinese and UK males. In Chinese inpatients, disorders of fluid, electrolyte, and acid–base balance (E87), disorders of amino-acid metabolism (E72), atherosclerosis (I70), liver diseases (K76), and hyperplasia of the prostate (N40) were specific hub diseases (Additional File 1: Table S8). Comorbidities covered by its *hub diseases’ associated network* represented 34.78% (410/1179) of unique comorbidity patterns and 73.16% (128,025/174,985) of frequency of comorbidities in the complete network. In UK inpatients, heart-related diseases including acute myocardial infarction (I21), chronic ischemic heart disease (I25), atrial fibrillation and flutter (I48), complications and ill-defined descriptions of heart disease (I51), and obesity (E66) were specific hub diseases. Comorbidities covered by its *hub diseases’ associated network* represented 35.16% (154/438) of unique comorbidity patterns and 54.15% (13,094/24,180) of frequency of comorbidities in the complete network (Fig. 3b, Table 1, Additional File 1: Table S4).

### Multimorbidity networks in Chinese and UK female inpatients

The *complete multimorbidity network* in Chinese female inpatients was 2.63 times more complex than in UK female inpatients (Fig. 3c, Additional File 1: Table S4, Additional File 3, China: 297 diseases/990 comorbidities; UK: 187 diseases/377 comorbidities). In Chinese inpatients, circulatory, metabolic, and genitourinary diseases-related comorbidities were most prevalent, while in UK, the most prevalent were circulatory, digestive, and genitourinary diseases-related comorbidities (Additional File 2: Figure S7.1–7.2). Among Chinese female inpatients, the most common comorbidity was chronic ischemic heart disease/heart failure comorbidity (I25 + I50, 2221/99,086), whereas polyp of female genital tract/excessive, frequent, and irregular menstruation (N84 + N92, 817/25,508) was the most common in UK female inpatients.

Among the top ten most common comorbidities among Chinese and UK female inpatients, there were no overlapped comorbidities. Among Chinese inpatients, the most common group of specific comorbidities was essential hypertension (I10)-related comorbidities (with cerebral infarction, I63; chronic ischemic heart disease, I25; type 2 diabetes mellitus, E11; liver diseases, K76; dyslipidemia, E78). In contrast, UK-specific comorbidities were mostly associated with diseases of the genitourinary system. Diaphragmatic hernia (K44)-related comorbidities (with gastritis and duodenitis, K29; gastroesophageal reflux disease, K21) were also specific in UK female inpatients (Fig. 4, Additional File 1: Table S7).

For hub diseases, dyslipidemia (E78), essential hypertension (I10), gastritis and duodenitis (K29), and leiomyoma of the uterus (D25) overlapped between Chinese and UK female inpatients. In Chinese female inpatients, disorders of fluid, electrolyte and acid–base balance (E87), type 2 diabetes mellitus (E11), liver diseases (K76), heart failure (I50), atherosclerosis (I70), and cerebral infarction (I63) were specific hub diseases (Additional File 1: Table S8). Comorbidities covered by its *hub diseases’ associated network* represented 35.05% (347/990) of unique comorbidity patterns and 65.00% (64,403/99,086) of frequency of comorbidities in the complete network. In UK female inpatients, obesity (E66), asthma (J45), genitourinary diseases including endometriosis (N80), polyp of the female genital tract (N84), noninflammatory disorders of cervix uteri (N85), and uterus, except cervix (N88), were specific hub diseases. Comorbidities covered by its *hub diseases’ associated network* represented 37.40% (141/377) of unique comorbidity patterns and 55.02% (14,034/25,508) of frequency of comorbidities in the complete network (Fig. 3c, Table 1, Additional File 1: Table S4).

### The metrics distribution of nodes in Chinese and UK inpatients

The hub diseases metrics were found to be ranked at the forefront of all node-related metrics in both Chinese and UK inpatients, suggesting that hub diseases are more likely to appear in inpatients with multimorbidity. Additional File 2: Figure S8 revealed a wider metric distribution among nodes in China, indicating greater complexity in multimorbidity networks compared to the UK (Additional File 3).

## Discussion

This study is the first to compare comorbidity patterns in Chinese and UK inpatients using multimorbidity networks. We observed higher per-capita disease counts and a greater proportion of multimorbidity among aging Chinese inpatients compared to their UK counterparts. Chinese inpatients consistently exhibit more complex multimorbidity networks. Comorbidities involving essential hypertension (I10), dyslipidemia (E78), type 2 diabetes mellitus (E11), and gastritis and duodenitis (K29) are most common in both populations. However, Chinese inpatients consistently demonstrate a higher frequency of comorbidities in circulatory diseases and endocrine/nutritional/metabolic diseases. In the UK, digestive diseases-related and genitourinary diseases-related comorbidities are also common, particularly the latter among female inpatients.

Our investigation reveals that Chinese inpatients exhibit a higher proportion of multimorbidity and a more intricate multimorbidity network compared to their UK counterparts. Notably, the lower socio-economic status of China, coupled with environmental and early life stressors associated with poverty and inadequate social infrastructure, may lead to the earlier onset of diseases, consequently resulting in a higher frequency of multimorbidity [1]. Besides, lower health literacy in China leads to delayed diagnosis and treatment of chronic diseases and comorbidities [1, 27, 28]. Lifestyle factors, such as tobacco use, unhealthy diets, and physical inactivity, are also more common in China [28, 29]. Furthermore, limited healthcare access and the absence of state-driven multimorbidity prevention programs in China result in greater disease burden and complexity [1]. Conversely, the UK has implemented comprehensive type 2 diabetes mellitus-related complication surveillance to manage multimorbidity in diabetic individuals [30]. The National Institute for Health and Care Excellence provides key healthcare services for adults with multimorbidity [31], a key resource currently lacking in China.

Our study confirms previous findings that circulatory and endocrine/nutritional/metabolic diseases-related comorbidities are prevalent in China [17, 32]. These patterns were echoed by a recent systematic review by Prados-Torres et al., where similar findings have been reported in 10 of the 14 multimorbidity studies [32]. The heightened prevalence of these comorbidities in China may be linked to the swift transformation of dietary patterns in the population. Western-style diets, increasingly popular in developed Chinese regions, have led to greater red and processed meat consumption [1, 33, 34]. Additionally, our data from Shaanxi Province may also indicate a common “starchy” dietary pattern, characterized by rice, noodles, and flour products in this Chinese region [35]. This dietary pattern may contribute to overweight and obesity, resulting in abnormal adipocytokine secretion and elevated risk of related comorbidities [36, 37]. Other lifestyle issues, including tobacco use, sedentary behavior, abnormal sleep duration, and low social participation, have also been identified as contributing risk factors to circulatory and endocrine/nutritional/metabolic diseases-related comorbidities in China [29, 34, 38, 39].

In the UK, there are multiple underlying reasons for the notable comorbidities related to digestive and genitourinary diseases. First, UK inpatients exhibit higher awareness of and greater access to clinics for diagnostic testing due to good public health literacy. UK clinicians also place greater emphasis on managing genitourinary and digestive diseases, resulting in more frequent diagnoses of these comorbidities. Second, the population-wide average of standard drinks consumed per day is higher in the UK than in China [40], with excessive alcohol consumption contributing to alterations in the gut microbiome and gut epithelial integrity that increase the likelihood of digestive disorders and their associated comorbidities [41–43]. Excessive alcohol consumption may also cause urinary system diseases such as chronic kidney disease [44, 45]. It may also contribute to risky sexual behavior and increased risk of sexually transmitted infections, leading to the development of genitourinary diseases and associated comorbidities [46]. Female UK inpatients have a high prevalence of genitourinary disease-related comorbidities, likely attributed to their older age. The older age means a higher likelihood of experiencing menopause, which is associated with a decrease in estrogen stimulation and the development of genitourinary disorders such as vaginitis, bladder dysfunction, and urethral dysfunction [47, 48]. Other potential contributing factors include tobacco use, a higher number of sexual partners, and higher fertility rates in the UK [29, 46, 48–51].

We discovered that prevalent diseases often serve as hub diseases in multimorbidity networks. We found that comorbidities covered by the hub diseases’ associated network account for over 68% and 55% frequency of comorbidities in the complete network for China and the UK, respectively. This finding highlights the potential importance of identifying hub diseases as a means of recognizing potential risk factors and underlying biological mechanisms for multimorbidity. Targeted surveillance and prevention of hub diseases could reduce the onset of associated comorbidities, improve healthcare utilization, and enable healthcare professionals to provide appropriate treatment plans [1]. This approach to care would shift from a single-condition treatment measurement to a patient-centered approach, allowing for comprehensive care and reducing the burden of polypharmacy. Policymakers can use these findings to establish more effective multimorbidity treatment guidelines, while patients can receive cost-effective treatment, and healthcare providers can enhance treatment efficiency and save medical resources [52, 53].

The main strength of our study is the important comparison of multimorbidity patterns in 14 ICD-10 disease chapters among middle-aged inpatients in China and the UK. However, several limitations still existed. First, we used undirected graphs based on baseline inpatient records, rather than directed graphs derived from inpatient cohort data. Our primary objective was to compare comorbidity patterns between two regions. However, conducting direct comparisons of temporal multimorbidity patterns between these regions posed challenges due to different observation periods. Second, our study, exclusively examining inpatients, may introduce sampling bias, limiting our understanding of multimorbidity. This hinders generalizability to outpatients with different patterns and factors. Third, the initial absolute number of diagnoses in different datasets which is related to the accessibility of different healthcare systems will affect the complexity of the multimorbidity network. Fourth, half of Chinese inpatients being blood donors may lead to underestimating disease prevalence due to the healthy donor effect, introducing sampling bias, and compromising the study’s representativeness for the general population, affecting its external validity. Fifth, the high prevalence of misdiagnosis and missed diagnosis in China may have underestimated comorbidity complexity. Sixth, the UK-Biobank population, being healthier and more health-literate than the general UK population, may underestimate disease prevalence and miss common comorbidity patterns, making it less representative of the broader population. Nevertheless, our conclusion remains that multimorbidity networks are more complex in Chinese inpatients than in those in the UK.

## Conclusions

In conclusion, we identified higher multimorbidity prevalence and more diverse comorbidity patterns and complex multimorbidity networks in Chinese inpatients, compared to the UK inpatients. While circulatory diseases and endocrine/nutritional/metabolic disease-related comorbidities are common in both Chinese and UK inpatients, digestive and genitourinary diseases-related comorbidities are more specific in UK inpatients. Hub diseases’ associated network accounts for the majority of comorbidity counts in the complete network in both China and the UK. Identifying common comorbidity patterns and hub diseases can inform prevention strategies, thus enhancing patient-centered care, reducing healthcare burden, and providing valuable insights for policymakers, patients, and treatment guidelines in different regions.

### Supplementary Information

**Additional file 1: **Additional Text: The explanation of network metrics. **Table S1.** ICD-10 chapters and corresponding content categories. Table S2. The number of different datasets. **Table S3.** The per-capita disease diagnoses from 1-14 ICD-10 chapters in overall, male and female inpatients among China and the UK. **Table S4.** Number of nodes and edges in the overall, male and female networks among Chinese and UK inpatients. **Table S5.** The odds ratios (ORs) and frequency of the statistically significant (*P* value, Bonferroni correction) and common (prevalence >1/10000) comorbidity patterns included in the complete multimorbidity network in Chinese overall inpatients (1367 patterns). **Table S6.** The odds ratios (ORs) and frequency of the statistically significant (*P* value, Bonferroni correction) and common (prevalence >1/10000) comorbidity patterns included in the complete multimorbidity network in the UK overall inpatients (467 patterns). **Table S7.** The most common comorbidity patterns in the overall, male and female inpatients among Chinese and UK inpatients (27 patterns). **Table S8.** The hub diseases in the overall, male and female inpatients among Chinese and UK inpatients (24 diseases). **Table S9.** The most common diseases (top10) in the overall, male and female inpatients among Chinese and UK inpatients (24 diseases).

**Additional file 2: Figure S1.** The records selection flowchart of the Shaanxi, China dataset. **Figure S2.** The records selection flowchart of the UK-Biobank dataset. **Figure S3.** The selection flowchart of the multimorbidity networks for the overall inpatients among Chinese inpatients. **Figure S4.** The selection flowchart of the multimorbidity networks for male and female among Chinese inpatients. **Figure S5.** The selection flowchart of the multimorbidity networks for the overall among UK inpatients. **Figure S6.** The selection flowchart of the multimorbidity networks for male and female among UK inpatients. **Figure S7.1.** The number of unique comorbidity patterns related to each chapter (ICD-10, 1–14 chapters) in overall, male and female inpatients in China and the United-Kingdom (UK). **Figure S7.2.** The total frequency of comorbidities related to each chapter (ICD-10, 1–14 chapters) in overall, male and female inpatients in China and the United-Kingdom (UK). **Figure S8.** Property distribution for all nodes and hub nodes of the over, male and female inpatients among China and UK. **Figure S9.** The proportion trend of ICD-10 1–14 chapters by age.

**Additional file 3.** The initial diseases included and the detailed information of nodes and edges among each multimorbidity networks.

## Data Availability

The data from Shaanxi, China, needs to be obtained by sending a data request application to the corresponding author’s email and requires approval from Shaanxi Provincial People’s Hospital. The data of UK Biobank are available in a public, open access repository. This research has been conducted using the UK Biobank Resource under application number 79244. The UK Biobank data are available on application to the UK Biobank (www.ukbiobank.ac.uk/accessed on 17 August 2022).

## Abbreviations

UK: United Kingdom
ICD-10: International Statistical Classification of Diseases and Related Health Problems 10th revision
CHMRs: Centralized Hospital Medical Records
HES: Hospital Episode Statistics
ORs: Odds ratios
MCC: Maximal clique centrality
Clo_Cen: Closeness centrality
Clu_Coe: Clustering coefficient
Bet_Cen: Betweenness centrality
