# Supplementary material for: Exploring multimorbidity profiles in middle-aged inpatients: a network-based comparative study of China and the United Kingdom
Source: BMC Med. 2023 Dec 13;21:495. doi: 10.1186/s12916-023-03204-y (PMC10720230; doi:10.1186/s12916-023-03204-y)
Supplement: Supplementary file 1 — Additional file 1: Additional Text: The explanation of network metrics. Table S1. ICD-10 chapters and corresponding content categories. Table S2. The number of different datasets. Table S3. The per-capita disease diagnoses from 1-14 ICD-10 chapters in overall, male and female inpatients among China and the UK. Table S4. Number of nodes and edges in the overall, male and female networks among Chinese and UK inpatients. Table S5. The odds ratios (ORs) and frequency of the statistically significant (P value, Bonferroni correction) and common (prevalence >1/10000) comorbidity patterns included in the complete multimorbidity network in Chinese overall inpatients (1367 patterns). Table S6. The odds ratios (ORs) and frequency of the statistically significant (P value, Bonferroni correction) and common (prevalence >1/10000) comorbidity patterns included in the complete multimorbidity network in the UK overall inpatients (467 patterns). Table S7. The most common comorbidity patterns in the overall, male and female inpatients among Chinese and UK inpatients (27 patterns). Table S8. The hub diseases in the overall, male and female inpatients among Chinese and UK inpatients (24 diseases). Table S9. The most common diseases (top10) in the overall, male and female inpatients among Chinese and UK inpatients (24 diseases). [file 12916_2023_3204_MOESM1_ESM.docx]

**Additional File 1**

**Additional Text: The explanation of network metrics.**

**1. Degree**

The degree of a node in a network is the number of edges the node connected to other nodes.

**2.** **Closeness centrality (Clo_Cen)**

Clo_Cen indicates nodes that can communicate quickly with other nodes of the network. Let G = (V, E) be an undirected graph. Then, the centrality is defined as:

$$Cclo\left( i \right)=\frac{1}{\sum_{t\in V}^{|V|} dist(i,j)}$$

where dist(i, j) denotes the distance or else the shortest path p between the nodes i and j.

**3. Clustering coefficient (Clu_Coe)**

The clustering coefficient assesses the connectivity in a node’s neighborhood: a node has a high clustering coefficient if its neighbors tend to be directly connected with each other. The coefficient is fundamental to assessing the small-world property, and it can be interpreted as an index of the redundancy of a node. A triangle is a subgraph of three nodes all connected to each other. It can be conceived of as a direct connection of a node j with a node q, given by (j, q), plus an indirect connection that travels through another node, i, given by (j, i, q). The local clustering coefficient was initially defined by Watts and Strogatz for unweighted networks as the number of connections among the neighbors of a focal node over the maximum possible number of such connections,

$C_{i,w}=\frac{\sum_{j,q} (a(j,i)a(i,q)a(j,q)}{k_{i}(k_{i}-1)}$

where 
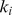
 is the degree of node i. The clustering coefficient can be equivalently conceived of as the number of triangles in the neighborhood of a focal node (t_i_), normalized by the maximum possible number of such triangles, and it can be interpreted as a measure of how much a focal node i is redundant in establishing connections in its neighborhood.

**4. Between centrality (Bet_Cen)**

**Betweenness Centrality**shows that nodes which are intermediate between neighbors rank higher. Without these nodes, there would be no way for two neighbors to communicate with each other. Thus, betweenness centrality shows important nodes that lie on a high proportion of paths between other nodes in the network. For distinct nodes i, j, w ∈ V(G), let σ_ij_be the total number of shortest paths between i and j and σ_ij_(w) be the number of shortest paths from i to j that pass through w. Moreover, for w ∈ V(G), let V (i) denote the set of all ordered pairs, (i, j) in V(G) × V(G) such that i, j, w are all distinct. Then, the Betweenness Centrality is calculated as:

$Cb\left( w \right)=\sum_{(i,j)\in V(w)} \frac{\sigma ij(w)}{\sigma ij}$.

**5. Pagerank**

PageRank is a famous ranking algorithm that forms the basis of the Google™ search engine. In practice, PageRank assigns a score *s_i_* to denote the attractiveness of the webpage *i*. Webpage *i* obtains a higher score if many other important webpages point to it. From the physical perspective, PageRank describes a random walk process on a directed network, where the score *s_i_* is proportional to the frequency of visits to a particular node *i* by a random walker. In the PageRank algorithm, the parameter *c* (0 ≤ *c* ≤ 1) called *return probability* is introduced, which represents the probability for a random walker to jump to a random node and (1 − *c*) is the probability for the random walker to continue walking through the directed links. In this way, the node *i*'s centrality score at time *t* (*t* ≥ 1) is given by:

$$Si\left( t \right)=c+\left( 1-c \right)\sum_{j=1}^{N} [\frac{Aij}{K_{j}^{out}}\left( 1-\delta_{K_{j}^{out},0} \right)+\frac{1}{N}\delta_{K_{j}^{out},0}]S_{j}(t-1)$$

where δ*_a_*_,_*_b_* = 1 when *a* = *b* and δ*_a_*_,_*_b_* = 0 otherwise. Initially, we assign each node one random walker, namely *s_i_*(0) = 1 for i=1, 2, ….., N The typical value of the return probability in computer science is approximately 0.15. The final score of each node is defined as the steady value after the convergence of *s_i_*(*t*). The final ranking of nodes in PageRank, denoted as *R_p_*, will be obtained by sorting *s_i_* in a descending order when *s_i_* reaches the stable state.

**6. Eigencentrality**

The heuristic behind the eigencentrality measure is based on the key idea that the centrality of a node is proportional to the sum of centralities of its neighbourhood and therefore, a node connected to central nodes, will also be central. As explained in the introductory section of this paper, this heuristic leads us to calculate the centrality of a node i by an equation of the form:

$$C_{i}=\frac{1}{\lambda_{max}}\sum_{j\epsilon N(i)} C_{j}=\frac{1}{\lambda_{max}}\sum_{j=1}^{n} A_{ij}c_{j,} i=1,2,\ldots,n$$

where λmax is the largest eigenvalue of the adjacency matrix A. Note that the above equation leads directly to the eigenvector problem:

$A_{c}=\lambda_{max}c$

where c exist and it is positive as consequence of the Perron-Frobenius theorem.

**7. Maximal clique centrality (MCC)**

MCC was calculated by a Cytoscape plugin cytohubba and it has been proved to have a better performance on the precision to rank the importance of the nodes in the network than other topological analyses. Given a node v, the MCC of v is defined as MCC(v) = ∑_C∈S(v)_(|C| - 1)!, where S(v) is the collection of maximal cliques which contain v, and (|C|-1)! is the product of all positive integers less than |C|.

**Table S1.** **ICD-10 Chapters and corresponding content categories.**

| Chapter | Abbreviation | Content |
| --- | --- | --- |
| Chapter1 | C1 | Certain infectious and parasitic diseases |
| Chapter2 | C2 | Neoplasms |
| Chapter3 | C3 | Diseases of the blood and blood-forming organs and certain disorders involving the immune mechanism |
| Chapter4 | C4 | Endocrine, nutritional and metabolic diseases |
| Chapter5 | C5 | Mental and behavioral disorders |
| Chapter6 | C6 | Diseases of the nervous system |
| Chapter7 | C7 | Diseases of the eye and adnexa |
| Chapter8 | C8 | Diseases of the ear and mastoid process |
| Chapter9 | C9 | Diseases of the circulatory system |
| Chapter10 | C10 | Diseases of the respiratory system |
| Chapter11 | C11 | Diseases of the digestive system |
| Chapter12 | C12 | Diseases of the skin and subcutaneous tissue |
| Chapter13 | C13 | Diseases of the musculoskeletal system and connective tissue |
| Chapter14 | C14 | Diseases of the genitourinary system |
| Chapter15 | C15 | Pregnancy, childbirth and the puerperium |
| Chapter16 | C16 | Certain conditions originating in the perinatal period |
| Chapter17 | C17 | Congenital malformations, deformations and chromosomal abnormalities |
| Chapter18 | C18 | Symptoms, signs and abnormal clinical and laboratory findings, not elsewhere classified |
| Chapter19 | C19 | Injury, poisoning and certain other consequences of external causes |
| Chapter20 | C20 | External causes of morbidity and mortality |
| Chapter21 | C21 | Factors influencing health status and contact with health services |
| Chapter22 | C22 | Codes for special purposes |

All diagnoses were coded with the International Statistical Classification of Diseases and Related Health Problems 10th revision (ICD-10). ICD-10 codes were grouped into 22 disease system chapters. However, our study excluded 15-22 chapters because these diseases are congenital, pregnancy, childbirth, puerperium, perinatal period diseases and symptoms, and causes, factors of diseases.

**Table S2. The number of** different data**sets.**

|  | China-Shannxi | | |
| --- | --- | --- | --- |
|  | Overall | Male | Female |
| The number of hospitalization records | 184,133 | 103,334 (56.12%) | 80,799 (43.88%) |
| Mean age (mean±standard deviation) | 48.45 (SD±5.29) | 48.27 (SD±5.31) | 48.67 (SD±5.27) |
|  | UK-Biobank | | |
|  | Overall | Male | Female |
| The number of hospitalization records | 180,497 | 79,652 (44.13%) | 100,845 (55.87%) |
| Mean age (mean±standard deviation) | 51.53 (SD±5.27) | 51.82 (SD±5.26) | 51.30 (SD±5.26) |

**Table S3. The per-capita disease diagnoses from 1-14 ICD-10 chapters in overall, male and female** **inpatients among China and the UK.**

|  |  |  | Chinese inpatients  (/person) | | | UK inpatients  (/person) | | |
| --- | --- | --- | --- | --- | --- | --- | --- | --- |
| Chapter | Abbreviation | Content | Overall | Male | Female | Overall | Male | Female |
| Chapter1 | C1 | Certain infectious and parasitic diseases | 0.05 | 0.06 | 0.05 | 0.005 | 0.006 | 0.004 |
| Chapter2 | C2 | Neoplasms | 0.10 | 0.05 | 0.15 | 0.07 | 0.05 | 0.09 |
| Chapter3 | C3 | Diseases of the blood and blood-forming organs and certain disorders involving the immune mechanism | 0.05 | 0.03 | 0.07 | 0.01 | 0.01 | 0.02 |
| Chapter4 | C4 | Endocrine, nutritional and metabolic diseases | 0.27 | 0.31 | 0.21 | 0.06 | 0.06 | 0.05 |
| Chapter5 | C5 | Mental and behavioral disorders | 0.02 | 0.02 | 0.02 | 0.03 | 0.03 | 0.02 |
| Chapter6 | C6 | Diseases of the nervous system | 0.11 | 0.12 | 0.10 | 0.04 | 0.04 | 0.04 |
| Chapter7 | C7 | Diseases of the eye and adnexa | 0.05 | 0.06 | 0.05 | 0.04 | 0.05 | 0.04 |
| Chapter8 | C8 | Diseases of the ear and mastoid process | 0.05 | 0.04 | 0.05 | 0.01 | 0.01 | 0.01 |
| Chapter9 | C9 | Diseases of the circulatory system | 0.62 | 0.72 | 0.49 | 0.21 | 0.29 | 0.14 |
| Chapter10 | C10 | Diseases of the respiratory system | 0.19 | 0.21 | 0.16 | 0.09 | 0.10 | 0.08 |
| Chapter11 | C11 | Diseases of the digestive system | 0.39 | 0.44 | 0.33 | 0.34 | 0.40 | 0.30 |
| Chapter12 | C12 | Diseases of the skin and subcutaneous tissue | 0.02 | 0.02 | 0.02 | 0.06 | 0.07 | 0.06 |
| Chapter13 | C13 | Diseases of the musculoskeletal system and connective tissue | 0.21 | 0.19 | 0.24 | 0.20 | 0.22 | 0.19 |
| Chapter14 | C14 | Diseases of the genitourinary system | 0.23 | 0.16 | 0.31 | 0.27 | 0.12 | 0.39 |

All diagnoses were coded with the International Statistical Classification of Diseases and Related Health Problems 10th revision (ICD-10). ICD-10 codes were grouped into 22 disease system chapters. However, our study excluded 15-22 chapters because these diseases are congenital, pregnancy, childbirth, puerperium, perinatal period diseases and symptoms, and the causes, and factors of diseases.

**Table S4. Number of nodes and edges in the overall, male and female networks among Chinese and UK inpatients.**

|  | Chinese inpatients | | | | | | UK inpatients | | | | | |
| --- | --- | --- | --- | --- | --- | --- | --- | --- | --- | --- | --- | --- |
|  | Overall | | Male | | Female | | Overall | | Male | | Female | |
|  | nodes | edges | nodes | edges | nodes | edges | nodes | edges | nodes | edges | nodes | edges |
| Complete network | 341 | 1367 | 320 | 1179 | 297 | 990 | 215 | 467 | 215 | 438 | 187 | 377 |
| Hub diseases’ direct network* | 10 | 32 | 10 | 35 | 10 | 31 | 10 | 13 | 10 | 28 | 10 | 16 |
| Hub diseases’ associated network | 193 | 483 | 173 | 410 | 160 | 347 | 95 | 176 | 73 | 154 | 79 | 141 |

* The networks directly formed by the hub diseases were defined as Hub diseases’ direct network.

**Table S5. The odds ratios (ORs) and frequency of the statistically significant (P value, Bonferroni correction) and common (prevalence >1/10000) comorbidity patterns included in the complete multimorbidity network in Chinese overall inpatients (1367 patterns).**

| Source | Target | SourceName | TargetName | Frequency | P | OR(95%CI) |
| --- | --- | --- | --- | --- | --- | --- |
| I10 | I63 | Essential (primary) hypertension | Cerebral infarction | 6595 | 0 | 5.16 (4.96, 5.36) |
| E78 | I10 | Disorders of lipoprotein metabolism and other lipidaemias | Essential (primary) hypertension | 6445 | 0 | 3.49 (3.36, 3.61) |
| I25 | I50 | Chronic ischaemic heart disease | Heart failure | 6174 | 0 | 61.32 (57.95, 64.91) |
| I10 | I25 | Essential (primary) hypertension | Chronic ischaemic heart disease | 5753 | 0 | 2.79 (2.69, 2.9) |
| E11 | I10 | Type 2 diabetes mellitus | Essential (primary) hypertension | 5454 | 0 | 2.07 (2, 2.15) |
| I10 | K76 | Essential (primary) hypertension | Other diseases of liver | 4377 | 2.68E-302 | 2.09 (2.01, 2.18) |
| E11 | E78 | Type 2 diabetes mellitus | Disorders of lipoprotein metabolism and other lipidaemias | 3841 | 0 | 4.02 (3.86, 4.19) |
| I10 | I50 | Essential (primary) hypertension | Heart failure | 3828 | 0 | 3.61 (3.45, 3.78) |
| E78 | K76 | Disorders of lipoprotein metabolism and other lipidaemias | Other diseases of liver | 3818 | 0 | 5.38 (5.15, 5.61) |
| E11 | K76 | Type 2 diabetes mellitus | Other diseases of liver | 3361 | 0 | 3.81 (3.64, 3.98) |
| I20 | I25 | Angina pectoris | Chronic ischaemic heart disease | 3317 | 0 | 77.59 (71.01, 84.91) |
| E11 | G63 | Type 2 diabetes mellitus | Polyneuropathy in diseases classified elsewhere | 2637 | 0 | 161.04 (139.16, 187.49) |
| I10 | I70 | Essential (primary) hypertension | Atherosclerosis | 2520 | 0 | 3.79 (3.57, 4.01) |
| I20 | I50 | Angina pectoris | Heart failure | 2450 | 0 | 45.87 (42.73, 49.25) |
| I10 | M47 | Essential (primary) hypertension | Spondylosis | 2414 | 3.78E-56 | 1.48 (1.41, 1.55) |
| G99 | M47 | Other disorders of nervous system in diseases classified elsewhere | Spondylosis | 2369 | 0 | 416.38 (351.67, 497.2) |
| E78 | I63 | Disorders of lipoprotein metabolism and other lipidaemias | Cerebral infarction | 2347 | 0 | 2.72 (2.59, 2.86) |
| E78 | I25 | Disorders of lipoprotein metabolism and other lipidaemias | Chronic ischaemic heart disease | 2143 | 4.96E-150 | 1.95 (1.85, 2.05) |
| E72 | I10 | Other disorders of amino-acid metabolism | Essential (primary) hypertension | 2050 | 0 | 4.96 (4.64, 5.3) |
| K29 | K76 | Gastritis and duodenitis | Other diseases of liver | 1933 | 1.02E-245 | 2.44 (2.31, 2.57) |
| E11 | I25 | Type 2 diabetes mellitus | Chronic ischaemic heart disease | 1869 | 5.81E-32 | 1.37 (1.3, 1.44) |
| E11 | I79 | Type 2 diabetes mellitus | Disorders of arteries, arterioles and capillaries in diseases classified elsewhere | 1765 | 0 | 239.82 (192.52, 303.35) |
| I10 | I20 | Essential (primary) hypertension | Angina pectoris | 1723 | 7.15E-225 | 2.91 (2.73, 3.11) |
| K76 | K80 | Other diseases of liver | Cholelithiasis | 1652 | 0 | 3.05 (2.88, 3.23) |
| E78 | K29 | Disorders of lipoprotein metabolism and other lipidaemias | Gastritis and duodenitis | 1631 | 7.77E-73 | 1.66 (1.57, 1.75) |
| E78 | M47 | Disorders of lipoprotein metabolism and other lipidaemias | Spondylosis | 1589 | 1.95E-202 | 2.42 (2.29, 2.57) |
| E11 | I63 | Type 2 diabetes mellitus | Cerebral infarction | 1576 | 9.64E-18 | 1.28 (1.21, 1.35) |
| I10 | I65 | Essential (primary) hypertension | Occlusion and stenosis of precerebral arteries, not resulting in cerebral infarction | 1536 | 0 | 4.67 (4.32, 5.05) |
| E78 | I70 | Disorders of lipoprotein metabolism and other lipidaemias | Atherosclerosis | 1458 | 0 | 4.49 (4.21, 4.79) |
| E11 | N08 | Type 2 diabetes mellitus | Glomerular disorders in diseases classified elsewhere | 1420 | 0 | 40.87 (36.38, 46.01) |
| D25 | N72 | Leiomyoma of uterus | Inflammatory disease of cervix uteri | 1408 | 0 | 12.53 (11.6, 13.52) |
| E72 | E78 | Other disorders of amino-acid metabolism | Disorders of lipoprotein metabolism and other lipidaemias | 1394 | 0 | 6.62 (6.18, 7.09) |
| I25 | I63 | Chronic ischaemic heart disease | Cerebral infarction | 1385 | 4.44E-11 | 1.22 (1.15, 1.3) |
| E78 | I50 | Disorders of lipoprotein metabolism and other lipidaemias | Heart failure | 1344 | 1.28E-126 | 2.13 (2, 2.27) |
| I25 | K76 | Chronic ischaemic heart disease | Other diseases of liver | 1341 | 1.50E-12 | 1.24 (1.17, 1.32) |
| M47 | M51 | Spondylosis | Other intervertebral disc disorders | 1301 | 2.26E-269 | 3.06 (2.88, 3.26) |
| I10 | I67 | Essential (primary) hypertension | Other cerebrovascular diseases | 1285 | 2.66E-131 | 2.44 (2.27, 2.62) |
| I84 | K62 | Haemorrhoids | Other diseases of anus and rectum | 1282 | 0 | 51.72 (47.13, 56.8) |
| E87 | I10 | Other disorders of fluid, electrolyte and acid-base balance | Essential (primary) hypertension | 1281 | 2.13E-118 | 2.29 (2.13, 2.46) |
| I10 | I49 | Essential (primary) hypertension | Other cardiac arrhythmias | 1278 | 4.66E-60 | 1.76 (1.64, 1.88) |
| E72 | I63 | Other disorders of amino-acid metabolism | Cerebral infarction | 1262 | 0 | 7.09 (6.59, 7.63) |
| K29 | K31 | Gastritis and duodenitis | Other diseases of stomach and duodenum | 1251 | 0 | 30.36 (27.5, 33.57) |
| I25 | K29 | Chronic ischaemic heart disease | Gastritis and duodenitis | 1250 | 1.83E-13 | 1.26 (1.18, 1.34) |
| G45 | I10 | Transient cerebral ischaemic attacks and related syndromes | Essential (primary) hypertension | 1247 | 2.97E-99 | 2.15 (2, 2.31) |
| E11 | I70 | Type 2 diabetes mellitus | Atherosclerosis | 1231 | 4.12E-207 | 2.89 (2.7, 3.1) |
| I63 | I70 | Cerebral infarction | Atherosclerosis | 1219 | 0 | 3.77 (3.52, 4.04) |
| E11 | I50 | Type 2 diabetes mellitus | Heart failure | 1188 | 4.05E-39 | 1.54 (1.44, 1.64) |
| E11 | H36 | Type 2 diabetes mellitus | Retinal disorders in diseases classified elsewhere | 1175 | 0 | 162.66 (129.99, 206.63) |
| H81 | I10 | Disorders of vestibular function | Essential (primary) hypertension | 1107 | 5.86E-07 | 1.19 (1.11, 1.28) |
| I25 | I49 | Chronic ischaemic heart disease | Other cardiac arrhythmias | 1105 | 0 | 4.22 (3.92, 4.54) |
| K29 | K80 | Gastritis and duodenitis | Cholelithiasis | 1076 | 1.27E-56 | 1.7 (1.59, 1.82) |
| I63 | M47 | Cerebral infarction | Spondylosis | 1071 | 5.16E-70 | 1.85 (1.73, 1.98) |
| I10 | I61 | Essential (primary) hypertension | Intracerebral haemorrhage | 1068 | 0 | 11.51 (10.22, 12.97) |
| G55 | M51 | Nerve root and plexus compressions in diseases classified elsewhere | Other intervertebral disc disorders | 1063 | 0 | 57.84 (51.32, 65.34) |
| I70 | K76 | Atherosclerosis | Other diseases of liver | 1061 | 8.06E-232 | 3.28 (3.05, 3.52) |
| I25 | I51 | Chronic ischaemic heart disease | Complications and ill-defined descriptions of heart disease | 1027 | 0 | 20.98 (18.92, 23.28) |
| I10 | I11 | Essential (primary) hypertension | Hypertensive heart disease | 1006 | 1.07E-263 | 47.13 (38.15, 58.98) |
| N13 | N20 | Obstructive and reflux uropathyC | Calculus of kidney and ureter | 997 | 0 | 61.64 (55.79, 68.12) |
| K21 | K29 | Gastro-oesophageal reflux disease | Gastritis and duodenitis | 942 | 0 | 23.57 (21.21, 26.22) |
| I25 | I70 | Chronic ischaemic heart disease | Atherosclerosis | 940 | 5.79E-100 | 2.24 (2.08, 2.42) |
| K76 | M47 | Other diseases of liver | Spondylosis | 938 | 1.92E-27 | 1.47 (1.37, 1.58) |
| E78 | I65 | Disorders of lipoprotein metabolism and other lipidaemias | Occlusion and stenosis of precerebral arteries, not resulting in cerebral infarction | 928 | 0 | 5.36 (4.94, 5.82) |
| K29 | M47 | Gastritis and duodenitis | Spondylosis | 909 | 4.21E-20 | 1.39 (1.29, 1.49) |
| I49 | I50 | Other cardiac arrhythmias | Heart failure | 908 | 0 | 6.19 (5.72, 6.69) |
| I63 | I65 | Cerebral infarction | Occlusion and stenosis of precerebral arteries, not resulting in cerebral infarction | 897 | 0 | 5.76 (5.29, 6.26) |
| I84 | K60 | Haemorrhoids | Fissure and fistula of anal and rectal regions | 895 | 0 | 22.52 (20.52, 24.71) |
| I21 | I25 | Acute myocardial infarction | Chronic ischaemic heart disease | 894 | 0 | 45.35 (39.28, 52.54) |
| I50 | I63 | Heart failure | Cerebral infarction | 893 | 1.79E-19 | 1.4 (1.3, 1.51) |
| E78 | H81 | Disorders of lipoprotein metabolism and other lipidaemias | Disorders of vestibular function | 873 | 3.54E-120 | 2.46 (2.28, 2.65) |
| G63 | K76 | Polyneuropathy in diseases classified elsewhere | Other diseases of liver | 869 | 0 | 5.38 (4.95, 5.84) |
| K80 | K81 | Cholelithiasis | Cholecystitis | 859 | 0 | 8.55 (7.87, 9.29) |
| G63 | I79 | Polyneuropathy in diseases classified elsewhere | Disorders of arteries, arterioles and capillaries in diseases classified elsewhere | 857 | 0 | 68.21 (61.51, 75.63) |
| E78 | G63 | Disorders of lipoprotein metabolism and other lipidaemias | Polyneuropathy in diseases classified elsewhere | 847 | 1.56E-276 | 4.52 (4.16, 4.91) |
| I50 | K76 | Heart failure | Other diseases of liver | 843 | 2.64E-17 | 1.38 (1.28, 1.48) |
| I10 | N40 | Essential (primary) hypertension | Hyperplasia of prostate | 831 | 2.38E-12 | 1.37 (1.26, 1.5) |
| G63 | I10 | Polyneuropathy in diseases classified elsewhere | Essential (primary) hypertension | 815 | 1.26E-18 | 1.46 (1.34, 1.58) |
| D25 | D64 | Leiomyoma of uterus | Other anaemias | 813 | 0 | 5.99 (5.49, 6.54) |
| K76 | N40 | Other diseases of liver | Hyperplasia of prostate | 806 | 1.02E-257 | 4.64 (4.26, 5.06) |
| K26 | K29 | Duodenal ulcer | Gastritis and duodenitis | 785 | 0 | 18.68 (16.77, 20.83) |
| K76 | N28 | Other diseases of liver | Other disorders of kidney and ureter, not elsewhere classified | 772 | 0 | 5.77 (5.28, 6.3) |
| E78 | G45 | Disorders of lipoprotein metabolism and other lipidaemias | Transient cerebral ischaemic attacks and related syndromes | 767 | 8.46E-153 | 3.01 (2.77, 3.26) |
| I50 | K29 | Heart failure | Gastritis and duodenitis | 766 | 1.20E-15 | 1.37 (1.27, 1.48) |
| E78 | I67 | Disorders of lipoprotein metabolism and other lipidaemias | Other cerebrovascular diseases | 739 | 5.24E-150 | 3.04 (2.79, 3.3) |
| H81 | M47 | Disorders of vestibular function | Spondylosis | 737 | 6.71E-168 | 3.15 (2.9, 3.41) |
| N72 | N73 | Inflammatory disease of cervix uteri | Other female pelvic inflammatory diseases | 725 | 0 | 8.01 (7.29, 8.8) |
| I10 | N28 | Essential (primary) hypertension | Other disorders of kidney and ureter, not elsewhere classified | 715 | 1.08E-23 | 1.58 (1.45, 1.73) |
| I79 | K76 | Disorders of arteries, arterioles and capillaries in diseases classified elsewhere | Other diseases of liver | 715 | 0 | 7.57 (6.88, 8.34) |
| E79 | I10 | Disorders of purine and pyrimidine metabolism | Essential (primary) hypertension | 712 | 2.74E-151 | 4.29 (3.84, 4.78) |
| I10 | I51 | Essential (primary) hypertension | Complications and ill-defined descriptions of heart disease | 711 | 3.95E-104 | 3.03 (2.74, 3.34) |
| E78 | E87 | Disorders of lipoprotein metabolism and other lipidaemias | Other disorders of fluid, electrolyte and acid-base balance | 703 | 5.51E-113 | 2.64 (2.42, 2.87) |
| E78 | I49 | Disorders of lipoprotein metabolism and other lipidaemias | Other cardiac arrhythmias | 690 | 1.08E-70 | 2.14 (1.96, 2.32) |
| K76 | K82 | Other diseases of liver | Other diseases of gallbladder | 674 | 1.30E-248 | 4.75 (4.33, 5.2) |
| E78 | E79 | Disorders of lipoprotein metabolism and other lipidaemias | Disorders of purine and pyrimidine metabolism | 672 | 0 | 10.24 (9.19, 11.41) |
| E78 | I20 | Disorders of lipoprotein metabolism and other lipidaemias | Angina pectoris | 661 | 6.55E-61 | 2.07 (1.9, 2.25) |
| G99 | I10 | Other disorders of nervous system in diseases classified elsewhere | Essential (primary) hypertension | 660 | 2.31E-20 | 1.54 (1.4, 1.69) |
| D25 | N83 | Leiomyoma of uterus | Noninflammatory disorders of ovary, fallopian tube and broad ligament | 653 | 0 | 8.77 (7.91, 9.71) |
| I50 | I70 | Heart failure | Atherosclerosis | 638 | 1.66E-102 | 2.62 (2.4, 2.86) |
| G45 | I63 | Transient cerebral ischaemic attacks and related syndromes | Cerebral infarction | 637 | 8.94E-117 | 2.88 (2.63, 3.15) |
| I10 | I69 | Essential (primary) hypertension | Sequelae of cerebrovascular disease | 630 | 1.39E-95 | 3.24 (2.9, 3.62) |
| J32 | J34 | Chronic sinusitis | Other disorders of nose and nasal sinuses | 623 | 0 | 81.62 (72.72, 91.57) |
| I63 | I67 | Cerebral infarction | Other cerebrovascular diseases | 620 | 3.72E-116 | 2.92 (2.66, 3.19) |
| G45 | M47 | Transient cerebral ischaemic attacks and related syndromes | Spondylosis | 614 | 4.59E-186 | 3.79 (3.46, 4.15) |
| E78 | I79 | Disorders of lipoprotein metabolism and other lipidaemias | Disorders of arteries, arterioles and capillaries in diseases classified elsewhere | 613 | 1.77E-228 | 5.15 (4.66, 5.68) |
| E72 | I70 | Other disorders of amino-acid metabolism | Atherosclerosis | 611 | 0 | 6.62 (6.03, 7.27) |
| I10 | N08 | Essential (primary) hypertension | Glomerular disorders in diseases classified elsewhere | 605 | 1.88E-37 | 1.93 (1.74, 2.13) |
| E11 | N40 | Type 2 diabetes mellitus | Hyperplasia of prostate | 585 | 3.19E-43 | 1.98 (1.8, 2.17) |
| E11 | I65 | Type 2 diabetes mellitus | Occlusion and stenosis of precerebral arteries, not resulting in cerebral infarction | 572 | 2.46E-61 | 2.23 (2.02, 2.45) |
| D25 | N73 | Leiomyoma of uterus | Other female pelvic inflammatory diseases | 570 | 4.02E-138 | 3.54 (3.21, 3.9) |
| E72 | K76 | Other disorders of amino-acid metabolism | Other diseases of liver | 568 | 4.59E-54 | 2.07 (1.88, 2.26) |
| I10 | I79 | Essential (primary) hypertension | Disorders of arteries, arterioles and capillaries in diseases classified elsewhere | 560 | 1.61E-16 | 1.54 (1.39, 1.7) |
| M48 | M51 | Other spondylopathies | Other intervertebral disc disorders | 550 | 0 | 8.13 (7.33, 9) |
| K29 | K81 | Gastritis and duodenitis | Cholecystitis | 528 | 2.04E-114 | 3.05 (2.77, 3.35) |
| H81 | I63 | Disorders of vestibular function | Cerebral infarction | 527 | 1.68E-25 | 1.65 (1.5, 1.82) |
| I25 | I65 | Chronic ischaemic heart disease | Occlusion and stenosis of precerebral arteries, not resulting in cerebral infarction | 527 | 1.34E-60 | 2.28 (2.06, 2.51) |
| E87 | K76 | Other disorders of fluid, electrolyte and acid-base balance | Other diseases of liver | 526 | 5.21E-54 | 2.1 (1.91, 2.31) |
| I65 | K76 | Occlusion and stenosis of precerebral arteries, not resulting in cerebral infarction | Other diseases of liver | 520 | 2.43E-87 | 2.71 (2.45, 2.98) |
| G63 | N08 | Polyneuropathy in diseases classified elsewhere | Glomerular disorders in diseases classified elsewhere | 519 | 0 | 27.65 (24.7, 30.9) |
| D64 | N72 | Other anaemias | Inflammatory disease of cervix uteri | 517 | 3.44E-189 | 4.75 (4.28, 5.26) |
| E11 | I20 | Type 2 diabetes mellitus | Angina pectoris | 513 | 5.91E-07 | 1.27 (1.15, 1.4) |
| I70 | M47 | Atherosclerosis | Spondylosis | 512 | 1.22E-53 | 2.12 (1.93, 2.33) |
| G55 | M47 | Nerve root and plexus compressions in diseases classified elsewhere | Spondylosis | 506 | 0 | 10.54 (9.42, 11.77) |
| E78 | N40 | Disorders of lipoprotein metabolism and other lipidaemias | Hyperplasia of prostate | 504 | 3.33E-47 | 2.16 (1.95, 2.39) |
| J31 | J34 | Chronic rhinitis, nasopharyngitis and pharyngitis | Other disorders of nose and nasal sinuses | 502 | 0 | 61.63 (54.62, 69.48) |
| E78 | N08 | Disorders of lipoprotein metabolism and other lipidaemias | Glomerular disorders in diseases classified elsewhere | 501 | 2.83E-149 | 4.06 (3.65, 4.5) |
| K25 | K29 | Gastric ulcer | Gastritis and duodenitis | 497 | 6.97E-297 | 8.43 (7.52, 9.44) |
| G63 | H36 | Polyneuropathy in diseases classified elsewhere | Retinal disorders in diseases classified elsewhere | 496 | 0 | 44.32 (39.18, 50.08) |
| E11 | E87 | Type 2 diabetes mellitus | Other disorders of fluid, electrolyte and acid-base balance | 493 | 8.40E-19 | 1.55 (1.41, 1.71) |
| E72 | I25 | Other disorders of amino-acid metabolism | Chronic ischaemic heart disease | 487 | 3.71E-19 | 1.56 (1.41, 1.72) |
| K35 | K65 | Acute appendicitis | Peritonitis | 483 | 0 | 102.11 (89.41, 116.59) |
| M50 | M51 | Cervical disc disorders | Other intervertebral disc disorders | 476 | 1.09E-107 | 3.06 (2.76, 3.37) |
| N72 | N83 | Inflammatory disease of cervix uteri | Noninflammatory disorders of ovary, fallopian tube and broad ligament | 472 | 7.13E-282 | 7.92 (7.07, 8.86) |
| E14 | I10 | Unspecified diabetes mellitus | Essential (primary) hypertension | 471 | 6.13E-19 | 1.65 (1.47, 1.84) |
| I11 | I50 | Hypertensive heart disease | Heart failure | 470 | 0 | 15.51 (13.69, 17.58) |
| K76 | K81 | Other diseases of liver | Cholecystitis | 467 | 1.00E-77 | 2.62 (2.37, 2.9) |
| N73 | N76 | Other female pelvic inflammatory diseases | Other inflammation of vagina and vulva | 467 | 0 | 19.44 (17.11, 22.06) |
| I10 | I21 | Essential (primary) hypertension | Acute myocardial infarction | 464 | 7.41E-49 | 2.48 (2.2, 2.8) |
| E87 | I63 | Other disorders of fluid, electrolyte and acid-base balance | Cerebral infarction | 460 | 5.59E-40 | 1.99 (1.79, 2.2) |
| K29 | K52 | Gastritis and duodenitis | Other noninfective gastroenteritis and colitis | 460 | 4.49E-122 | 3.48 (3.13, 3.86) |
| E78 | M50 | Disorders of lipoprotein metabolism and other lipidaemias | Cervical disc disorders | 458 | 2.62E-33 | 1.85 (1.67, 2.05) |
| I21 | I50 | Acute myocardial infarction | Heart failure | 458 | 0 | 13.75 (12.14, 15.56) |
| K29 | K63 | Gastritis and duodenitis | Other diseases of intestine | 457 | 6.64E-256 | 7.6 (6.76, 8.53) |
| D25 | N70 | Leiomyoma of uterus | Salpingitis and oophoritis | 452 | 0 | 15.2 (13.27, 17.41) |
| E78 | G99 | Disorders of lipoprotein metabolism and other lipidaemias | Other disorders of nervous system in diseases classified elsewhere | 444 | 4.91E-65 | 2.48 (2.23, 2.75) |
| K76 | N08 | Other diseases of liver | Glomerular disorders in diseases classified elsewhere | 441 | 9.40E-127 | 3.83 (3.43, 4.27) |
| E72 | M47 | Other disorders of amino-acid metabolism | Spondylosis | 439 | 1.88E-78 | 2.67 (2.41, 2.96) |
| I49 | K76 | Other cardiac arrhythmias | Other diseases of liver | 435 | 2.11E-11 | 1.41 (1.27, 1.56) |
| I67 | M47 | Other cerebrovascular diseases | Spondylosis | 432 | 1.33E-75 | 2.64 (2.38, 2.93) |
| K76 | N20 | Other diseases of liver | Calculus of kidney and ureter | 428 | 8.05E-11 | 1.41 (1.27, 1.56) |
| E87 | I25 | Other disorders of fluid, electrolyte and acid-base balance | Chronic ischaemic heart disease | 425 | 1.56E-14 | 1.5 (1.35, 1.66) |
| K29 | K82 | Gastritis and duodenitis | Other diseases of gallbladder | 425 | 1.41E-75 | 2.7 (2.42, 2.99) |
| J18 | K76 | Pneumonia, organism unspecified | Other diseases of liver | 414 | 3.32E-12 | 1.44 (1.3, 1.6) |
| E11 | N28 | Type 2 diabetes mellitus | Other disorders of kidney and ureter, not elsewhere classified | 413 | 7.13E-28 | 1.83 (1.64, 2.04) |
| E78 | J06 | Disorders of lipoprotein metabolism and other lipidaemias | Acute upper respiratory infections of multiple and unspecified sites | 413 | 3.88E-17 | 1.56 (1.4, 1.73) |
| I47 | I49 | Paroxysmal tachycardia | Other cardiac arrhythmias | 413 | 0 | 43.6 (37.96, 50.06) |
| N72 | N76 | Inflammatory disease of cervix uteri | Other inflammation of vagina and vulva | 411 | 0 | 11.98 (10.54, 13.6) |
| H36 | I10 | Retinal disorders in diseases classified elsewhere | Essential (primary) hypertension | 407 | 1.29E-19 | 1.75 (1.55, 1.97) |
| D25 | D50 | Leiomyoma of uterus | Iron deficiency anaemia | 403 | 1.09E-177 | 5.89 (5.21, 6.64) |
| E72 | G45 | Other disorders of amino-acid metabolism | Transient cerebral ischaemic attacks and related syndromes | 396 | 1.40E-234 | 6.42 (5.73, 7.16) |
| E78 | N28 | Disorders of lipoprotein metabolism and other lipidaemias | Other disorders of kidney and ureter, not elsewhere classified | 394 | 1.46E-35 | 2.02 (1.8, 2.25) |
| D25 | N84 | Leiomyoma of uterus | Polyp of female genital tract | 391 | 4.46E-203 | 7.02 (6.19, 7.96) |
| E87 | K29 | Other disorders of fluid, electrolyte and acid-base balance | Gastritis and duodenitis | 391 | 4.08E-16 | 1.55 (1.39, 1.72) |
| I21 | I51 | Acute myocardial infarction | Complications and ill-defined descriptions of heart disease | 389 | 0 | 67.32 (58.53, 77.36) |
| I79 | N08 | Disorders of arteries, arterioles and capillaries in diseases classified elsewhere | Glomerular disorders in diseases classified elsewhere | 389 | 0 | 29.41 (25.91, 33.32) |
| I49 | K29 | Other cardiac arrhythmias | Gastritis and duodenitis | 387 | 2.68E-07 | 1.32 (1.18, 1.46) |
| E78 | K82 | Disorders of lipoprotein metabolism and other lipidaemias | Other diseases of gallbladder | 371 | 1.03E-29 | 1.9 (1.7, 2.13) |
| E79 | K76 | Disorders of purine and pyrimidine metabolism | Other diseases of liver | 368 | 4.20E-118 | 4.19 (3.71, 4.73) |
| J31 | J32 | Chronic rhinitis, nasopharyngitis and pharyngitis | Chronic sinusitis | 365 | 0 | 24.86 (21.94, 28.12) |
| E72 | H81 | Other disorders of amino-acid metabolism | Disorders of vestibular function | 362 | 1.38E-141 | 4.35 (3.88, 4.87) |
| E72 | I65 | Other disorders of amino-acid metabolism | Occlusion and stenosis of precerebral arteries, not resulting in cerebral infarction | 362 | 6.19E-218 | 6.64 (5.89, 7.45) |
| K80 | K85 | Cholelithiasis | Acute pancreatitis | 362 | 2.66E-228 | 7.64 (6.75, 8.64) |
| I67 | K76 | Other cerebrovascular diseases | Other diseases of liver | 360 | 3.30E-11 | 1.45 (1.3, 1.62) |
| K29 | N40 | Gastritis and duodenitis | Hyperplasia of prostate | 359 | 6.98E-36 | 2.11 (1.88, 2.36) |
| H81 | I70 | Disorders of vestibular function | Atherosclerosis | 356 | 7.87E-72 | 2.83 (2.53, 3.17) |
| I50 | I65 | Heart failure | Occlusion and stenosis of precerebral arteries, not resulting in cerebral infarction | 356 | 4.00E-60 | 2.62 (2.33, 2.93) |
| D25 | N80 | Leiomyoma of uterus | Endometriosis | 355 | 1.74E-201 | 8.43 (7.34, 9.66) |
| N70 | N72 | Salpingitis and oophoritis | Inflammatory disease of cervix uteri | 355 | 8.97E-303 | 14.59 (12.66, 16.78) |
| E11 | K82 | Type 2 diabetes mellitus | Other diseases of gallbladder | 351 | 2.63E-17 | 1.64 (1.46, 1.84) |
| E87 | I50 | Other disorders of fluid, electrolyte and acid-base balance | Heart failure | 350 | 1.63E-44 | 2.25 (2.01, 2.52) |
| J06 | K76 | Acute upper respiratory infections of multiple and unspecified sites | Other diseases of liver | 350 | 5.37E-12 | 1.48 (1.32, 1.65) |
| G47 | I10 | Sleep disorders | Essential (primary) hypertension | 349 | 1.50E-33 | 2.28 (1.99, 2.6) |
| E72 | I50 | Other disorders of amino-acid metabolism | Heart failure | 348 | 3.56E-31 | 1.96 (1.75, 2.19) |
| N72 | N93 | Inflammatory disease of cervix uteri | Other abnormal uterine and vaginal bleeding | 343 | 1.24E-167 | 5.99 (5.27, 6.8) |
| I65 | M47 | Occlusion and stenosis of precerebral arteries, not resulting in cerebral infarction | Spondylosis | 337 | 5.87E-57 | 2.59 (2.3, 2.91) |
| I67 | K29 | Other cerebrovascular diseases | Gastritis and duodenitis | 336 | 2.72E-09 | 1.41 (1.26, 1.58) |
| E72 | I67 | Other disorders of amino-acid metabolism | Other cerebrovascular diseases | 335 | 6.74E-169 | 5.39 (4.78, 6.06) |
| J06 | K29 | Acute upper respiratory infections of multiple and unspecified sites | Gastritis and duodenitis | 333 | 7.96E-09 | 1.4 (1.24, 1.56) |
| H36 | N08 | Retinal disorders in diseases classified elsewhere | Glomerular disorders in diseases classified elsewhere | 328 | 0 | 38.58 (33.56, 44.25) |
| K76 | K85 | Other diseases of liver | Acute pancreatitis | 328 | 4.24E-109 | 4.25 (3.74, 4.82) |
| N20 | N28 | Calculus of kidney and ureter | Other disorders of kidney and ureter, not elsewhere classified | 326 | 2.22E-213 | 7.1 (6.26, 8.01) |
| G63 | I70 | Polyneuropathy in diseases classified elsewhere | Atherosclerosis | 324 | 2.80E-105 | 3.86 (3.41, 4.35) |
| E78 | H36 | Disorders of lipoprotein metabolism and other lipidaemias | Retinal disorders in diseases classified elsewhere | 323 | 2.89E-83 | 3.57 (3.13, 4.05) |
| I20 | I70 | Angina pectoris | Atherosclerosis | 322 | 1.11E-53 | 2.56 (2.27, 2.88) |
| I63 | M50 | Cerebral infarction | Cervical disc disorders | 320 | 9.02E-13 | 1.55 (1.37, 1.74) |
| G99 | I63 | Other disorders of nervous system in diseases classified elsewhere | Cerebral infarction | 316 | 2.60E-31 | 2.07 (1.83, 2.34) |
| N72 | N80 | Inflammatory disease of cervix uteri | Endometriosis | 311 | 7.60E-220 | 10.11 (8.75, 11.65) |
| D64 | N93 | Other anaemias | Other abnormal uterine and vaginal bleeding | 310 | 7.55E-166 | 6.45 (5.65, 7.35) |
| I70 | N40 | Atherosclerosis | Hyperplasia of prostate | 309 | 3.02E-70 | 3.16 (2.78, 3.58) |
| H36 | I79 | Retinal disorders in diseases classified elsewhere | Disorders of arteries, arterioles and capillaries in diseases classified elsewhere | 306 | 0 | 32.11 (27.86, 36.93) |
| K29 | N28 | Gastritis and duodenitis | Other disorders of kidney and ureter, not elsewhere classified | 306 | 3.00E-26 | 1.93 (1.71, 2.18) |
| I20 | I49 | Angina pectoris | Other cardiac arrhythmias | 302 | 7.14E-85 | 3.39 (2.99, 3.83) |
| G45 | I70 | Transient cerebral ischaemic attacks and related syndromes | Atherosclerosis | 300 | 2.13E-69 | 3.04 (2.69, 3.44) |
| K31 | K76 | Other diseases of stomach and duodenum | Other diseases of liver | 300 | 1.23E-50 | 2.61 (2.3, 2.96) |
| N73 | N83 | Other female pelvic inflammatory diseases | Noninflammatory disorders of ovary, fallopian tube and broad ligament | 299 | 8.49E-126 | 5.06 (4.43, 5.77) |
| I20 | I51 | Angina pectoris | Complications and ill-defined descriptions of heart disease | 297 | 2.40E-248 | 9.64 (8.44, 10.99) |
| I49 | I70 | Other cardiac arrhythmias | Atherosclerosis | 297 | 9.35E-49 | 2.51 (2.21, 2.83) |
| E78 | I51 | Disorders of lipoprotein metabolism and other lipidaemias | Complications and ill-defined descriptions of heart disease | 295 | 1.75E-36 | 2.28 (2, 2.58) |
| D25 | N93 | Leiomyoma of uterus | Other abnormal uterine and vaginal bleeding | 294 | 6.50E-68 | 3.21 (2.81, 3.66) |
| K22 | K29 | Other diseases of oesophagus | Gastritis and duodenitis | 289 | 1.44E-225 | 28.7 (23.43, 35.33) |
| M51 | M54 | Other intervertebral disc disorders | Dorsalgia | 287 | 2.65E-89 | 3.72 (3.27, 4.23) |
| J32 | J33 | Chronic sinusitis | Nasal polyp | 286 | 0 | 278.66 (221.83, 352.77) |
| E03 | I10 | Other hypothyroidism | Essential (primary) hypertension | 284 | 1.45E-13 | 1.7 (1.47, 1.95) |
| I61 | I63 | Intracerebral haemorrhage | Cerebral infarction | 284 | 2.25E-57 | 2.99 (2.61, 3.42) |
| N70 | N83 | Salpingitis and oophoritis | Noninflammatory disorders of ovary, fallopian tube and broad ligament | 282 | 0 | 21.59 (18.52, 25.1) |
| I42 | I50 | Cardiomyopathy | Heart failure | 281 | 4.83E-286 | 38.99 (32.02, 47.62) |
| N28 | N40 | Other disorders of kidney and ureter, not elsewhere classified | Hyperplasia of prostate | 281 | 2.16E-175 | 7.13 (6.21, 8.15) |
| I84 | K61 | Haemorrhoids | Abscess of anal and rectal regions | 280 | 9.94E-133 | 5.32 (4.64, 6.07) |
| K29 | K92 | Gastritis and duodenitis | Other diseases of digestive system | 276 | 2.37E-109 | 4.83 (4.19, 5.54) |
| I10 | N18 | Essential (primary) hypertension | Chronic kidney disease | 275 | 1.19E-31 | 2.48 (2.13, 2.88) |
| I48 | I50 | Atrial fibrillation and flutter | Heart failure | 275 | 7.71E-240 | 16.79 (14.2, 19.84) |
| K80 | K82 | Cholelithiasis | Other diseases of gallbladder | 275 | 4.17E-38 | 2.3 (2.02, 2.61) |
| N72 | N84 | Inflammatory disease of cervix uteri | Polyp of female genital tract | 275 | 5.07E-144 | 6.3 (5.46, 7.24) |
| G45 | I65 | Transient cerebral ischaemic attacks and related syndromes | Occlusion and stenosis of precerebral arteries, not resulting in cerebral infarction | 273 | 1.02E-137 | 5.34 (4.67, 6.08) |
| K52 | K76 | Other noninfective gastroenteritis and colitis | Other diseases of liver | 272 | 1.86E-18 | 1.77 (1.56, 2.01) |
| K21 | K76 | Gastro-oesophageal reflux disease | Other diseases of liver | 268 | 7.37E-44 | 2.58 (2.25, 2.94) |
| N20 | N39 | Calculus of kidney and ureter | Other disorders of urinary system | 267 | 5.17E-224 | 9.27 (8.07, 10.61) |
| E78 | I11 | Disorders of lipoprotein metabolism and other lipidaemias | Hypertensive heart disease | 266 | 3.78E-62 | 3.29 (2.86, 3.78) |
| H36 | K76 | Retinal disorders in diseases classified elsewhere | Other diseases of liver | 266 | 1.83E-59 | 3.14 (2.73, 3.59) |
| I50 | J44 | Heart failure | Other chronic obstructive pulmonary disease | 264 | 3.26E-57 | 2.99 (2.61, 3.42) |
| H35 | I10 | Other retinal disorders | Essential (primary) hypertension | 262 | 8.32E-22 | 2.11 (1.81, 2.46) |
| D25 | N85 | Leiomyoma of uterus | Other noninflammatory disorders of uterus, except cervix | 261 | 1.40E-125 | 6.41 (5.5, 7.45) |
| D64 | N83 | Other anaemias | Noninflammatory disorders of ovary, fallopian tube and broad ligament | 259 | 3.10E-90 | 4.22 (3.67, 4.84) |
| K60 | K62 | Fissure and fistula of anal and rectal regions | Other diseases of anus and rectum | 257 | 2.07E-287 | 13.43 (11.65, 15.43) |
| G99 | M51 | Other disorders of nervous system in diseases classified elsewhere | Other intervertebral disc disorders | 256 | 1.23E-25 | 2.01 (1.76, 2.29) |
| E11 | E79 | Type 2 diabetes mellitus | Disorders of purine and pyrimidine metabolism | 255 | 4.72E-22 | 1.98 (1.72, 2.27) |
| G99 | H81 | Other disorders of nervous system in diseases classified elsewhere | Disorders of vestibular function | 254 | 4.28E-90 | 3.93 (3.43, 4.49) |
| I50 | J98 | Heart failure | Other respiratory disorders | 254 | 2.03E-29 | 2.14 (1.87, 2.44) |
| M47 | M50 | Spondylosis | Cervical disc disorders | 254 | 3.74E-09 | 1.47 (1.29, 1.68) |
| E14 | E78 | Unspecified diabetes mellitus | Disorders of lipoprotein metabolism and other lipidaemias | 253 | 1.15E-23 | 2.01 (1.75, 2.29) |
| I67 | I70 | Other cerebrovascular diseases | Atherosclerosis | 253 | 2.01E-44 | 2.59 (2.26, 2.96) |
| E77 | E87 | Disorders of glycoprotein metabolism | Other disorders of fluid, electrolyte and acid-base balance | 251 | 0 | 18.53 (15.94, 21.46) |
| K20 | K29 | Oesophagitis | Gastritis and duodenitis | 251 | 1.29E-188 | 37.65 (29.67, 48.23) |
| N72 | N85 | Inflammatory disease of cervix uteri | Other noninflammatory disorders of uterus, except cervix | 251 | 2.86E-168 | 9.01 (7.7, 10.51) |
| I10 | I47 | Essential (primary) hypertension | Paroxysmal tachycardia | 250 | 5.80E-13 | 1.73 (1.49, 2.01) |
| I11 | I63 | Hypertensive heart disease | Cerebral infarction | 249 | 2.67E-63 | 3.51 (3.03, 4.06) |
| D64 | N73 | Other anaemias | Other female pelvic inflammatory diseases | 244 | 9.12E-29 | 2.22 (1.93, 2.55) |
| H81 | M50 | Disorders of vestibular function | Cervical disc disorders | 243 | 2.14E-50 | 2.77 (2.42, 3.17) |
| A49 | K29 | Bacterial infection of unspecified site | Gastritis and duodenitis | 242 | 7.22E-147 | 8.49 (7.21, 9.98) |
| E78 | M48 | Disorders of lipoprotein metabolism and other lipidaemias | Other spondylopathies | 242 | 3.52E-14 | 1.69 (1.47, 1.94) |
| I10 | M10 | Essential (primary) hypertension | Gout | 242 | 8.59E-25 | 2.36 (2, 2.78) |
| E03 | E78 | Other hypothyroidism | Disorders of lipoprotein metabolism and other lipidaemias | 241 | 2.02E-67 | 3.69 (3.18, 4.27) |
| D50 | K29 | Iron deficiency anaemia | Gastritis and duodenitis | 240 | 1.22E-27 | 2.18 (1.89, 2.5) |
| D50 | N72 | Iron deficiency anaemia | Inflammatory disease of cervix uteri | 237 | 8.81E-81 | 4.18 (3.61, 4.83) |
| K80 | K83 | Cholelithiasis | Other diseases of biliary tract | 234 | 6.03E-213 | 17.08 (14.28, 20.42) |
| I63 | J32 | Cerebral infarction | Chronic sinusitis | 233 | 5.56E-17 | 1.82 (1.58, 2.09) |
| D18 | K76 | Haemangioma and lymphangioma, any site | Other diseases of liver | 232 | 4.45E-52 | 3.07 (2.65, 3.55) |
| D64 | E87 | Other anaemias | Other disorders of fluid, electrolyte and acid-base balance | 232 | 5.22E-72 | 3.59 (3.12, 4.12) |
| E87 | J18 | Other disorders of fluid, electrolyte and acid-base balance | Pneumonia, organism unspecified | 231 | 3.33E-58 | 3.08 (2.67, 3.52) |
| E11 | I51 | Type 2 diabetes mellitus | Complications and ill-defined descriptions of heart disease | 230 | 8.17E-08 | 1.47 (1.27, 1.69) |
| K74 | K76 | Fibrosis and cirrhosis of liver | Other diseases of liver | 228 | 1.89E-28 | 2.25 (1.94, 2.59) |
| B18 | K76 | Chronic viral hepatitis | Other diseases of liver | 226 | 5.24E-14 | 1.72 (1.49, 1.97) |
| D50 | N93 | Iron deficiency anaemia | Other abnormal uterine and vaginal bleeding | 223 | 8.24E-182 | 9.91 (8.47, 11.55) |
| G93 | I10 | Other disorders of brain | Essential (primary) hypertension | 222 | 3.69E-16 | 1.97 (1.67, 2.32) |
| K81 | K82 | Cholecystitis | Other diseases of gallbladder | 222 | 3.96E-147 | 6.59 (5.7, 7.59) |
| E78 | K31 | Disorders of lipoprotein metabolism and other lipidaemias | Other diseases of stomach and duodenum | 221 | 1.74E-09 | 1.55 (1.34, 1.78) |
| D25 | N76 | Leiomyoma of uterus | Other inflammation of vagina and vulva | 220 | 1.21E-49 | 3.13 (2.69, 3.64) |
| J43 | J44 | Emphysema | Other chronic obstructive pulmonary disease | 220 | 4.60E-301 | 20.12 (17.14, 23.54) |
| J31 | J38 | Chronic rhinitis, nasopharyngitis and pharyngitis | Diseases of vocal cords and larynx, not elsewhere classified | 217 | 0 | 36.18 (30.71, 42.48) |
| I63 | I69 | Cerebral infarction | Sequelae of cerebrovascular disease | 215 | 1.75E-21 | 2.07 (1.78, 2.4) |
| E11 | H26 | Type 2 diabetes mellitus | Other cataract | 214 | 4.39E-34 | 2.62 (2.24, 3.05) |
| I48 | I49 | Atrial fibrillation and flutter | Other cardiac arrhythmias | 214 | 3.90E-282 | 23.13 (19.46, 27.43) |
| K80 | N28 | Cholelithiasis | Other disorders of kidney and ureter, not elsewhere classified | 214 | 1.13E-18 | 1.9 (1.64, 2.18) |
| E87 | I49 | Other disorders of fluid, electrolyte and acid-base balance | Other cardiac arrhythmias | 212 | 3.74E-40 | 2.62 (2.26, 3.01) |
| E79 | I63 | Disorders of purine and pyrimidine metabolism | Cerebral infarction | 210 | 1.40E-24 | 2.2 (1.89, 2.56) |
| K63 | K76 | Other diseases of intestine | Other diseases of liver | 210 | 3.48E-28 | 2.32 (1.99, 2.69) |
| M47 | N40 | Spondylosis | Hyperplasia of prostate | 210 | 6.35E-14 | 1.76 (1.52, 2.03) |
| N20 | N40 | Calculus of kidney and ureter | Hyperplasia of prostate | 210 | 6.88E-56 | 3.3 (2.84, 3.81) |
| E11 | I21 | Type 2 diabetes mellitus | Acute myocardial infarction | 209 | 1.33E-13 | 1.78 (1.52, 2.07) |
| B18 | K74 | Chronic viral hepatitis | Fibrosis and cirrhosis of liver | 207 | 1.11E-279 | 17.3 (14.76, 20.19) |
| E78 | G44 | Disorders of lipoprotein metabolism and other lipidaemias | Other headache syndromes | 203 | 1.02E-28 | 2.37 (2.03, 2.76) |
| E78 | K21 | Disorders of lipoprotein metabolism and other lipidaemias | Gastro-oesophageal reflux disease | 203 | 1.03E-10 | 1.63 (1.4, 1.89) |
| I10 | I12 | Essential (primary) hypertension | Hypertensive renal disease | 202 | 2.47E-68 | 9.46 (7.38, 12.22) |
| K26 | K76 | Duodenal ulcer | Other diseases of liver | 202 | 7.47E-20 | 2.02 (1.73, 2.35) |
| B18 | K29 | Chronic viral hepatitis | Gastritis and duodenitis | 200 | 5.83E-10 | 1.59 (1.37, 1.84) |
| I65 | I67 | Occlusion and stenosis of precerebral arteries, not resulting in cerebral infarction | Other cerebrovascular diseases | 200 | 5.58E-69 | 3.83 (3.29, 4.44) |
| N70 | N73 | Salpingitis and oophoritis | Other female pelvic inflammatory diseases | 200 | 1.84E-122 | 7.38 (6.24, 8.68) |
| H81 | I65 | Disorders of vestibular function | Occlusion and stenosis of precerebral arteries, not resulting in cerebral infarction | 199 | 2.13E-42 | 2.82 (2.42, 3.26) |
| E79 | I25 | Disorders of purine and pyrimidine metabolism | Chronic ischaemic heart disease | 197 | 1.97E-13 | 1.78 (1.52, 2.07) |
| I25 | I48 | Chronic ischaemic heart disease | Atrial fibrillation and flutter | 197 | 5.93E-72 | 5 (4.19, 5.96) |
| I65 | I70 | Occlusion and stenosis of precerebral arteries, not resulting in cerebral infarction | Atherosclerosis | 197 | 6.49E-22 | 2.08 (1.78, 2.41) |
| D64 | N80 | Other anaemias | Endometriosis | 194 | 1.10E-103 | 6.24 (5.28, 7.33) |
| I63 | M48 | Cerebral infarction | Other spondylopathies | 193 | 3.36E-09 | 1.59 (1.36, 1.85) |
| E78 | K85 | Disorders of lipoprotein metabolism and other lipidaemias | Acute pancreatitis | 192 | 1.98E-16 | 1.92 (1.64, 2.24) |
| I25 | I47 | Chronic ischaemic heart disease | Paroxysmal tachycardia | 192 | 2.62E-49 | 3.46 (2.92, 4.07) |
| N13 | N39 | Obstructive and reflux uropathyC | Other disorders of urinary system | 192 | 1.30E-228 | 13.79 (11.73, 16.13) |
| D18 | K29 | Haemangioma and lymphangioma, any site | Gastritis and duodenitis | 191 | 2.57E-29 | 2.43 (2.08, 2.84) |
| K21 | K31 | Gastro-oesophageal reflux disease | Other diseases of stomach and duodenum | 191 | 1.29E-259 | 16.8 (14.27, 19.68) |
| E72 | I49 | Other disorders of amino-acid metabolism | Other cardiac arrhythmias | 190 | 6.29E-24 | 2.16 (1.85, 2.5) |
| K74 | K92 | Fibrosis and cirrhosis of liver | Other diseases of digestive system | 189 | 0 | 28.21 (23.77, 33.32) |
| E04 | K76 | Other nontoxic goitre | Other diseases of liver | 188 | 1.18E-34 | 2.71 (2.3, 3.16) |
| E14 | K76 | Unspecified diabetes mellitus | Other diseases of liver | 188 | 1.32E-09 | 1.61 (1.37, 1.87) |
| I11 | K76 | Hypertensive heart disease | Other diseases of liver | 188 | 5.13E-27 | 2.4 (2.04, 2.81) |
| E72 | E87 | Other disorders of amino-acid metabolism | Other disorders of fluid, electrolyte and acid-base balance | 187 | 2.84E-33 | 2.53 (2.17, 2.94) |
| I47 | I50 | Paroxysmal tachycardia | Heart failure | 186 | 7.38E-101 | 6.17 (5.21, 7.28) |
| E14 | G63 | Unspecified diabetes mellitus | Polyneuropathy in diseases classified elsewhere | 185 | 3.99E-143 | 8.02 (6.81, 9.38) |
| E78 | G47 | Disorders of lipoprotein metabolism and other lipidaemias | Sleep disorders | 184 | 1.67E-26 | 2.41 (2.04, 2.82) |
| I45 | I49 | Other conduction disorders | Other cardiac arrhythmias | 184 | 1.09E-235 | 20.02 (16.71, 23.92) |
| I70 | N28 | Atherosclerosis | Other disorders of kidney and ureter, not elsewhere classified | 183 | 4.80E-30 | 2.46 (2.1, 2.87) |
| E11 | H52 | Type 2 diabetes mellitus | Disorders of refraction and accommodation | 182 | 1.24E-40 | 3.19 (2.69, 3.78) |
| G45 | M50 | Transient cerebral ischaemic attacks and related syndromes | Cervical disc disorders | 182 | 1.20E-42 | 2.92 (2.5, 3.4) |
| I10 | I48 | Essential (primary) hypertension | Atrial fibrillation and flutter | 182 | 1.21E-06 | 1.56 (1.3, 1.86) |
| A98 | N08 | Other viral haemorrhagic fevers, not elsewhere classified | Glomerular disorders in diseases classified elsewhere | 181 | 3.39E-23 | 21339.4 (4788.17, 375533.77) |
| J18 | J94 | Pneumonia, organism unspecified | Other pleural conditions | 181 | 1.60E-110 | 6.12 (5.2, 7.15) |
| D64 | N70 | Other anaemias | Salpingitis and oophoritis | 180 | 3.45E-98 | 6.23 (5.25, 7.35) |
| G63 | N40 | Polyneuropathy in diseases classified elsewhere | Hyperplasia of prostate | 179 | 9.25E-45 | 3.21 (2.72, 3.75) |
| I10 | I66 | Essential (primary) hypertension | Occlusion and stenosis of cerebral arteries, not resulting in cerebral infarction | 179 | 3.31E-42 | 4.78 (3.82, 6) |
| E87 | J98 | Other disorders of fluid, electrolyte and acid-base balance | Other respiratory disorders | 176 | 3.51E-54 | 3.45 (2.94, 4.02) |
| K25 | K76 | Gastric ulcer | Other diseases of liver | 176 | 3.19E-15 | 1.9 (1.62, 2.23) |
| I24 | I25 | Other acute ischaemic heart diseases | Chronic ischaemic heart disease | 175 | 8.91E-113 | 13.66 (10.9, 17.16) |
| N13 | N28 | Obstructive and reflux uropathyC | Other disorders of kidney and ureter, not elsewhere classified | 174 | 3.90E-137 | 8.01 (6.78, 9.41) |
| E11 | I11 | Type 2 diabetes mellitus | Hypertensive heart disease | 173 | 7.16E-09 | 1.63 (1.37, 1.92) |
| E87 | I70 | Other disorders of fluid, electrolyte and acid-base balance | Atherosclerosis | 173 | 5.10E-11 | 1.69 (1.44, 1.97) |
| D64 | N84 | Other anaemias | Polyp of female genital tract | 172 | 3.06E-61 | 4.14 (3.49, 4.88) |
| J43 | K76 | Emphysema | Other diseases of liver | 172 | 1.31E-12 | 1.82 (1.54, 2.14) |
| N73 | N93 | Other female pelvic inflammatory diseases | Other abnormal uterine and vaginal bleeding | 172 | 2.90E-37 | 2.98 (2.52, 3.5) |
| D64 | N18 | Other anaemias | Chronic kidney disease | 171 | 1.38E-220 | 19.49 (16.18, 23.36) |
| I27 | I50 | Other pulmonary heart diseases | Heart failure | 171 | 6.90E-166 | 26.2 (20.77, 33.12) |
| N73 | N80 | Other female pelvic inflammatory diseases | Endometriosis | 171 | 1.65E-75 | 5.11 (4.28, 6.06) |
| G45 | G99 | Transient cerebral ischaemic attacks and related syndromes | Other disorders of nervous system in diseases classified elsewhere | 170 | 8.34E-56 | 3.61 (3.06, 4.22) |
| I70 | M50 | Atherosclerosis | Cervical disc disorders | 169 | 5.18E-17 | 1.99 (1.69, 2.33) |
| I63 | I66 | Cerebral infarction | Occlusion and stenosis of cerebral arteries, not resulting in cerebral infarction | 167 | 7.38E-113 | 14.04 (11.17, 17.67) |
| J94 | J98 | Other pleural conditions | Other respiratory disorders | 167 | 1.00E-135 | 8.19 (6.92, 9.65) |
| E78 | G51 | Disorders of lipoprotein metabolism and other lipidaemias | Facial nerve disorders | 166 | 7.69E-15 | 1.93 (1.63, 2.28) |
| E78 | G54 | Disorders of lipoprotein metabolism and other lipidaemias | Nerve root and plexus disorders | 165 | 2.45E-08 | 1.59 (1.35, 1.87) |
| K76 | K92 | Other diseases of liver | Other diseases of digestive system | 165 | 1.02E-19 | 2.18 (1.83, 2.57) |
| I67 | M50 | Other cerebrovascular diseases | Cervical disc disorders | 164 | 1.04E-35 | 2.79 (2.36, 3.27) |
| G54 | M51 | Nerve root and plexus disorders | Other intervertebral disc disorders | 163 | 2.68E-26 | 2.44 (2.06, 2.87) |
| E03 | E11 | Other hypothyroidism | Type 2 diabetes mellitus | 162 | 1.75E-19 | 2.21 (1.86, 2.62) |
| M51 | M89 | Other intervertebral disc disorders | Other disorders of bone | 162 | 3.43E-69 | 4.8 (4.02, 5.71) |
| E03 | K76 | Other hypothyroidism | Other diseases of liver | 161 | 2.78E-29 | 2.67 (2.24, 3.16) |
| I49 | I65 | Other cardiac arrhythmias | Occlusion and stenosis of precerebral arteries, not resulting in cerebral infarction | 161 | 4.79E-26 | 2.41 (2.04, 2.83) |
| I20 | I65 | Angina pectoris | Occlusion and stenosis of precerebral arteries, not resulting in cerebral infarction | 160 | 1.47E-21 | 2.23 (1.88, 2.62) |
| H34 | I10 | Retinal vascular occlusions | Essential (primary) hypertension | 159 | 1.08E-27 | 3.3 (2.66, 4.08) |
| I70 | N08 | Atherosclerosis | Glomerular disorders in diseases classified elsewhere | 159 | 2.26E-36 | 2.93 (2.47, 3.46) |
| E72 | E79 | Other disorders of amino-acid metabolism | Disorders of purine and pyrimidine metabolism | 158 | 8.33E-81 | 5.24 (4.4, 6.2) |
| E87 | J06 | Other disorders of fluid, electrolyte and acid-base balance | Acute upper respiratory infections of multiple and unspecified sites | 158 | 4.61E-26 | 2.4 (2.03, 2.82) |
| F41 | I10 | Other anxiety disorders | Essential (primary) hypertension | 158 | 1.23E-06 | 1.58 (1.31, 1.9) |
| G31 | I63 | Other degenerative diseases of nervous system, not elsewhere classified | Cerebral infarction | 158 | 6.34E-89 | 10.58 (8.4, 13.33) |
| K52 | K63 | Other noninfective gastroenteritis and colitis | Other diseases of intestine | 158 | 1.30E-178 | 12.36 (10.36, 14.65) |
| M48 | M50 | Other spondylopathies | Cervical disc disorders | 158 | 1.06E-85 | 5.33 (4.5, 6.29) |
| D64 | E77 | Other anaemias | Disorders of glycoprotein metabolism | 157 | 1.71E-165 | 12.94 (10.75, 15.49) |
| E11 | M81 | Type 2 diabetes mellitus | Osteoporosis without pathological fracture | 156 | 1.57E-26 | 2.71 (2.25, 3.24) |
| N80 | N83 | Endometriosis | Noninflammatory disorders of ovary, fallopian tube and broad ligament | 156 | 1.42E-110 | 7.92 (6.59, 9.46) |
| G45 | I67 | Transient cerebral ischaemic attacks and related syndromes | Other cerebrovascular diseases | 155 | 2.56E-22 | 2.26 (1.91, 2.66) |
| I27 | J44 | Other pulmonary heart diseases | Other chronic obstructive pulmonary disease | 155 | 0 | 104.66 (82.55, 132.74) |
| E04 | E78 | Other nontoxic goitre | Disorders of lipoprotein metabolism and other lipidaemias | 153 | 4.03E-10 | 1.74 (1.46, 2.06) |
| I79 | N40 | Disorders of arteries, arterioles and capillaries in diseases classified elsewhere | Hyperplasia of prostate | 152 | 4.26E-55 | 4.13 (3.45, 4.9) |
| N83 | N93 | Noninflammatory disorders of ovary, fallopian tube and broad ligament | Other abnormal uterine and vaginal bleeding | 152 | 3.29E-61 | 4.43 (3.7, 5.26) |
| J33 | J34 | Nasal polyp | Other disorders of nose and nasal sinuses | 151 | 0 | 78.53 (63.3, 97.17) |
| C22 | K74 | Malignant neoplasm of liver and intrahepatic bile ducts | Fibrosis and cirrhosis of liver | 150 | 0 | 107.96 (85.86, 135.62) |
| E79 | I50 | Disorders of purine and pyrimidine metabolism | Heart failure | 150 | 1.35E-22 | 2.37 (1.98, 2.81) |
| G47 | K76 | Sleep disorders | Other diseases of liver | 150 | 1.70E-16 | 2.09 (1.75, 2.48) |
| K65 | K80 | Peritonitis | Cholelithiasis | 150 | 8.76E-39 | 3.18 (2.66, 3.78) |
| E72 | I20 | Other disorders of amino-acid metabolism | Angina pectoris | 149 | 5.43E-08 | 1.59 (1.34, 1.87) |
| K29 | K74 | Gastritis and duodenitis | Fibrosis and cirrhosis of liver | 148 | 7.33E-07 | 1.54 (1.29, 1.82) |
| D64 | N85 | Other anaemias | Other noninflammatory disorders of uterus, except cervix | 147 | 1.10E-67 | 5.17 (4.29, 6.19) |
| G44 | I63 | Other headache syndromes | Cerebral infarction | 146 | 1.17E-16 | 2.12 (1.77, 2.53) |
| G44 | M47 | Other headache syndromes | Spondylosis | 146 | 1.73E-25 | 2.54 (2.12, 3.01) |
| E04 | E11 | Other nontoxic goitre | Type 2 diabetes mellitus | 145 | 7.38E-08 | 1.63 (1.36, 1.94) |
| I44 | I49 | Atrioventricular and left bundle-branch block | Other cardiac arrhythmias | 145 | 5.40E-200 | 27.21 (21.92, 33.68) |
| K21 | K26 | Gastro-oesophageal reflux disease | Duodenal ulcer | 145 | 3.06E-180 | 14.2 (11.8, 16.97) |
| D73 | K74 | Diseases of spleen | Fibrosis and cirrhosis of liver | 144 | 0 | 162.23 (126.18, 208.89) |
| E66 | I10 | Obesity | Essential (primary) hypertension | 144 | 4.38E-45 | 5.97 (4.66, 7.66) |
| I70 | K82 | Atherosclerosis | Other diseases of gallbladder | 144 | 1.44E-18 | 2.17 (1.81, 2.56) |
| E02 | E78 | Subclinical iodine-deficiency hypothyroidism | Disorders of lipoprotein metabolism and other lipidaemias | 142 | 1.98E-62 | 5.68 (4.63, 6.95) |
| K29 | K85 | Gastritis and duodenitis | Acute pancreatitis | 142 | 5.79E-08 | 1.62 (1.36, 1.93) |
| N03 | N18 | Chronic nephritic syndrome | Chronic kidney disease | 142 | 0 | 83.86 (68.22, 102.56) |
| D25 | N88 | Leiomyoma of uterus | Other noninflammatory disorders of cervix uteri | 141 | 8.43E-45 | 3.93 (3.23, 4.74) |
| D50 | N83 | Iron deficiency anaemia | Noninflammatory disorders of ovary, fallopian tube and broad ligament | 141 | 2.74E-59 | 4.6 (3.82, 5.51) |
| E02 | I10 | Subclinical iodine-deficiency hypothyroidism | Essential (primary) hypertension | 141 | 7.09E-12 | 2.06 (1.67, 2.53) |
| E72 | M50 | Other disorders of amino-acid metabolism | Cervical disc disorders | 141 | 6.87E-22 | 2.34 (1.96, 2.78) |
| G31 | I10 | Other degenerative diseases of nervous system, not elsewhere classified | Essential (primary) hypertension | 140 | 5.97E-16 | 2.59 (2.06, 3.25) |
| I65 | N40 | Occlusion and stenosis of precerebral arteries, not resulting in cerebral infarction | Hyperplasia of prostate | 140 | 2.15E-21 | 2.39 (1.99, 2.85) |
| J44 | J96 | Other chronic obstructive pulmonary disease | Respiratory failure, not elsewhere classified | 140 | 3.81E-267 | 42.94 (34.7, 52.89) |
| K26 | K92 | Duodenal ulcer | Other diseases of digestive system | 139 | 1.91E-206 | 19.18 (15.82, 23.09) |
| N20 | N23 | Calculus of kidney and ureter | Unspecified renal colic | 139 | 1.40E-176 | 110.35 (80.23, 153.99) |
| E11 | H35 | Type 2 diabetes mellitus | Other retinal disorders | 138 | 3.21E-15 | 2.13 (1.76, 2.56) |
| H36 | I70 | Retinal disorders in diseases classified elsewhere | Atherosclerosis | 137 | 2.15E-42 | 3.57 (2.96, 4.27) |
| I10 | I42 | Essential (primary) hypertension | Cardiomyopathy | 136 | 3.56E-07 | 1.7 (1.38, 2.08) |
| I50 | J43 | Heart failure | Emphysema | 136 | 2.99E-17 | 2.2 (1.83, 2.64) |
| I61 | J98 | Intracerebral haemorrhage | Other respiratory disorders | 136 | 3.27E-93 | 6.67 (5.54, 7.97) |
| K82 | N40 | Other diseases of gallbladder | Hyperplasia of prostate | 136 | 4.10E-46 | 3.76 (3.12, 4.5) |
| N13 | N23 | Obstructive and reflux uropathyC | Unspecified renal colic | 135 | 1.17E-244 | 226.64 (165.81, 313.67) |
| E78 | I47 | Disorders of lipoprotein metabolism and other lipidaemias | Paroxysmal tachycardia | 134 | 9.93E-14 | 2.02 (1.67, 2.42) |
| E78 | M10 | Disorders of lipoprotein metabolism and other lipidaemias | Gout | 133 | 1.33E-22 | 2.61 (2.15, 3.16) |
| I51 | I70 | Complications and ill-defined descriptions of heart disease | Atherosclerosis | 133 | 1.29E-27 | 2.74 (2.28, 3.28) |
| E79 | I70 | Disorders of purine and pyrimidine metabolism | Atherosclerosis | 132 | 7.00E-36 | 3.25 (2.69, 3.89) |
| I24 | I50 | Other acute ischaemic heart diseases | Heart failure | 132 | 1.85E-113 | 14.23 (11.29, 17.88) |
| I10 | I24 | Essential (primary) hypertension | Other acute ischaemic heart diseases | 131 | 5.57E-17 | 2.64 (2.1, 3.31) |
| E87 | K52 | Other disorders of fluid, electrolyte and acid-base balance | Other noninfective gastroenteritis and colitis | 130 | 2.36E-34 | 3.07 (2.55, 3.67) |
| J18 | J43 | Pneumonia, organism unspecified | Emphysema | 130 | 4.24E-69 | 5.3 (4.38, 6.36) |
| D24 | N62 | Benign neoplasm of breast | Hypertrophy of breast | 129 | 3.18E-204 | 27.61 (22.25, 34.07) |
| I25 | I45 | Chronic ischaemic heart disease | Other conduction disorders | 129 | 9.85E-30 | 3.19 (2.6, 3.88) |
| E83 | E87 | Disorders of mineral metabolism | Other disorders of fluid, electrolyte and acid-base balance | 128 | 3.68E-207 | 39.8 (31.42, 50.31) |
| E87 | I65 | Other disorders of fluid, electrolyte and acid-base balance | Occlusion and stenosis of precerebral arteries, not resulting in cerebral infarction | 128 | 2.73E-19 | 2.3 (1.91, 2.75) |
| K29 | K56 | Gastritis and duodenitis | Paralytic ileus and intestinal obstruction without hernia | 128 | 2.30E-13 | 2.01 (1.66, 2.41) |
| G63 | N28 | Polyneuropathy in diseases classified elsewhere | Other disorders of kidney and ureter, not elsewhere classified | 127 | 1.28E-32 | 3.05 (2.53, 3.66) |
| E79 | M47 | Disorders of purine and pyrimidine metabolism | Spondylosis | 126 | 5.57E-14 | 2.04 (1.68, 2.44) |
| I98 | K74 | Other disorders of circulatory system in diseases classified elsewhere | Fibrosis and cirrhosis of liver | 125 | 1.91E-22 | 18009.97 (4018.76, 317432.52) |
| K60 | K61 | Fissure and fistula of anal and rectal regions | Abscess of anal and rectal regions | 125 | 3.90E-78 | 6.21 (5.11, 7.49) |
| H26 | H52 | Other cataract | Disorders of refraction and accommodation | 124 | 2.04E-275 | 41.01 (33.28, 50.18) |
| D50 | K92 | Iron deficiency anaemia | Other diseases of digestive system | 123 | 2.85E-203 | 25.15 (20.36, 30.85) |
| I84 | K63 | Haemorrhoids | Other diseases of intestine | 123 | 4.50E-30 | 2.99 (2.47, 3.6) |
| N73 | N84 | Other female pelvic inflammatory diseases | Polyp of female genital tract | 123 | 8.98E-25 | 2.76 (2.27, 3.33) |
| A16 | J98 | Respiratory tuberculosis, not confirmed bacteriologically or histologically | Other respiratory disorders | 122 | 9.69E-92 | 7.3 (5.99, 8.8) |
| E87 | G45 | Other disorders of fluid, electrolyte and acid-base balance | Transient cerebral ischaemic attacks and related syndromes | 122 | 1.73E-08 | 1.69 (1.4, 2.02) |
| G47 | I25 | Sleep disorders | Chronic ischaemic heart disease | 122 | 5.74E-07 | 1.64 (1.34, 1.98) |
| D18 | K80 | Haemangioma and lymphangioma, any site | Cholelithiasis | 121 | 3.37E-13 | 2.01 (1.66, 2.42) |
| E87 | J20 | Other disorders of fluid, electrolyte and acid-base balance | Acute bronchitis | 121 | 1.21E-08 | 1.7 (1.41, 2.04) |
| G99 | I65 | Other disorders of nervous system in diseases classified elsewhere | Occlusion and stenosis of precerebral arteries, not resulting in cerebral infarction | 121 | 7.76E-38 | 3.45 (2.84, 4.15) |
| M51 | M81 | Other intervertebral disc disorders | Osteoporosis without pathological fracture | 121 | 7.32E-34 | 3.38 (2.76, 4.1) |
| E11 | N18 | Type 2 diabetes mellitus | Chronic kidney disease | 120 | 7.72E-10 | 1.86 (1.52, 2.26) |
| I11 | I70 | Hypertensive heart disease | Atherosclerosis | 120 | 5.76E-38 | 3.6 (2.95, 4.36) |
| M17 | M51 | Gonarthrosis [arthrosis of knee] | Other intervertebral disc disorders | 120 | 9.17E-24 | 2.71 (2.22, 3.27) |
| N70 | N80 | Salpingitis and oophoritis | Endometriosis | 120 | 3.56E-128 | 12.96 (10.5, 15.88) |
| N83 | N85 | Noninflammatory disorders of ovary, fallopian tube and broad ligament | Other noninflammatory disorders of uterus, except cervix | 120 | 2.02E-76 | 6.78 (5.52, 8.27) |
| H81 | M48 | Disorders of vestibular function | Other spondylopathies | 119 | 1.26E-20 | 2.44 (2.01, 2.94) |
| K76 | M10 | Other diseases of liver | Gout | 119 | 1.36E-18 | 2.46 (2, 2.99) |
| K81 | K85 | Cholecystitis | Acute pancreatitis | 119 | 2.59E-83 | 6.7 (5.5, 8.09) |
| E06 | K76 | Thyroiditis | Other diseases of liver | 118 | 4.84E-36 | 3.69 (2.99, 4.5) |
| E79 | E87 | Disorders of purine and pyrimidine metabolism | Other disorders of fluid, electrolyte and acid-base balance | 118 | 6.35E-53 | 4.5 (3.69, 5.43) |
| K61 | K62 | Abscess of anal and rectal regions | Other diseases of anus and rectum | 118 | 8.16E-82 | 6.79 (5.56, 8.23) |
| K82 | N28 | Other diseases of gallbladder | Other disorders of kidney and ureter, not elsewhere classified | 118 | 1.19E-41 | 3.71 (3.05, 4.47) |
| B37 | N77 | Candidiasis | Vulvovaginal ulceration and inflammation in diseases classified elsewhere | 117 | 9.05E-254 | 5893.7 (3636.12, 9908.42) |
| J18 | J44 | Pneumonia, organism unspecified | Other chronic obstructive pulmonary disease | 117 | 9.12E-30 | 3.01 (2.48, 3.63) |
| N08 | N40 | Glomerular disorders in diseases classified elsewhere | Hyperplasia of prostate | 117 | 1.91E-33 | 3.35 (2.74, 4.06) |
| E72 | I11 | Other disorders of amino-acid metabolism | Hypertensive heart disease | 116 | 6.47E-57 | 4.95 (4.04, 6) |
| G40 | I63 | Epilepsy | Cerebral infarction | 116 | 1.74E-23 | 2.85 (2.31, 3.49) |
| I45 | I50 | Other conduction disorders | Heart failure | 116 | 1.05E-52 | 5.09 (4.12, 6.25) |
| I49 | I51 | Other cardiac arrhythmias | Complications and ill-defined descriptions of heart disease | 116 | 1.01E-30 | 3.08 (2.53, 3.72) |
| E16 | I10 | Other disorders of pancreatic internal secretion | Essential (primary) hypertension | 115 | 3.82E-19 | 3.16 (2.45, 4.06) |
| K29 | K59 | Gastritis and duodenitis | Other functional intestinal disorders | 115 | 6.51E-58 | 6.18 (4.93, 7.7) |
| K31 | K63 | Other diseases of stomach and duodenum | Other diseases of intestine | 115 | 3.45E-122 | 10.85 (8.85, 13.18) |
| D25 | N71 | Leiomyoma of uterus | Inflammatory disease of uterus, except cervix | 114 | 5.01E-58 | 6.41 (5.1, 8.03) |
| E11 | E66 | Type 2 diabetes mellitus | Obesity | 113 | 2.80E-63 | 8.65 (6.72, 11.1) |
| E87 | I61 | Other disorders of fluid, electrolyte and acid-base balance | Intracerebral haemorrhage | 113 | 1.67E-44 | 4.03 (3.3, 4.88) |
| I25 | I44 | Chronic ischaemic heart disease | Atrioventricular and left bundle-branch block | 113 | 1.51E-38 | 4.58 (3.63, 5.74) |
| E87 | I67 | Other disorders of fluid, electrolyte and acid-base balance | Other cerebrovascular diseases | 112 | 5.16E-07 | 1.63 (1.34, 1.96) |
| K74 | K80 | Fibrosis and cirrhosis of liver | Cholelithiasis | 112 | 2.62E-07 | 1.66 (1.36, 2.01) |
| E11 | E55 | Type 2 diabetes mellitus | Vitamin D deficiency | 111 | 3.52E-68 | 37.56 (25.32, 57.32) |
| E66 | E78 | Obesity | Disorders of lipoprotein metabolism and other lipidaemias | 110 | 7.26E-63 | 8.32 (6.48, 10.64) |
| F41 | K29 | Other anxiety disorders | Gastritis and duodenitis | 110 | 1.06E-23 | 2.9 (2.34, 3.55) |
| G93 | I61 | Other disorders of brain | Intracerebral haemorrhage | 110 | 2.63E-187 | 23.85 (19.19, 29.39) |
| J45 | K76 | Asthma | Other diseases of liver | 110 | 1.90E-07 | 1.7 (1.38, 2.07) |
| N72 | N81 | Inflammatory disease of cervix uteri | Female genital prolapse | 110 | 1.64E-92 | 11.75 (9.26, 14.84) |
| M13 | M51 | Other arthritis | Other intervertebral disc disorders | 108 | 2.58E-43 | 4.46 (3.59, 5.5) |
| N83 | N84 | Noninflammatory disorders of ovary, fallopian tube and broad ligament | Polyp of female genital tract | 108 | 3.41E-40 | 4.01 (3.25, 4.9) |
| E78 | M89 | Disorders of lipoprotein metabolism and other lipidaemias | Other disorders of bone | 107 | 3.50E-08 | 1.78 (1.44, 2.18) |
| D70 | K29 | Agranulocytosis | Gastritis and duodenitis | 106 | 3.75E-13 | 2.15 (1.74, 2.63) |
| E02 | K76 | Subclinical iodine-deficiency hypothyroidism | Other diseases of liver | 106 | 7.35E-40 | 4.5 (3.58, 5.59) |
| I61 | J69 | Intracerebral haemorrhage | Pneumonitis due to solids and liquids | 106 | 1.52E-251 | 147.15 (110.32, 196.59) |
| J18 | J96 | Pneumonia, organism unspecified | Respiratory failure, not elsewhere classified | 106 | 1.64E-117 | 13.29 (10.62, 16.49) |
| M47 | M75 | Spondylosis | Shoulder lesions | 106 | 9.24E-45 | 4.73 (3.79, 5.85) |
| N71 | N72 | Inflammatory disease of uterus, except cervix | Inflammatory disease of cervix uteri | 106 | 1.04E-72 | 8.56 (6.75, 10.77) |
| D24 | N60 | Benign neoplasm of breast | Benign mammary dysplasia | 104 | 1.04E-151 | 21.48 (17.02, 26.9) |
| G63 | I65 | Polyneuropathy in diseases classified elsewhere | Occlusion and stenosis of precerebral arteries, not resulting in cerebral infarction | 104 | 1.50E-11 | 2 (1.62, 2.43) |
| I11 | I51 | Hypertensive heart disease | Complications and ill-defined descriptions of heart disease | 104 | 1.25E-110 | 10.9 (8.79, 13.37) |
| I11 | M47 | Hypertensive heart disease | Spondylosis | 104 | 1.48E-10 | 1.95 (1.58, 2.38) |
| N84 | N93 | Polyp of female genital tract | Other abnormal uterine and vaginal bleeding | 104 | 4.83E-45 | 4.5 (3.63, 5.52) |
| D26 | N72 | Other benign neoplasms of uterus | Inflammatory disease of cervix uteri | 103 | 8.70E-63 | 7.17 (5.67, 9) |
| E72 | I69 | Other disorders of amino-acid metabolism | Sequelae of cerebrovascular disease | 103 | 2.64E-31 | 3.39 (2.75, 4.15) |
| E87 | J94 | Other disorders of fluid, electrolyte and acid-base balance | Other pleural conditions | 103 | 9.64E-34 | 3.5 (2.84, 4.27) |
| I10 | I71 | Essential (primary) hypertension | Aortic aneurysm and dissection | 103 | 3.82E-31 | 7.06 (5.1, 9.88) |
| I84 | L30 | Haemorrhoids | Other dermatitis | 103 | 2.89E-71 | 7.57 (6.03, 9.41) |
| I10 | J69 | Essential (primary) hypertension | Pneumonitis due to solids and liquids | 102 | 8.51E-25 | 4.44 (3.34, 5.9) |
| I79 | N28 | Disorders of arteries, arterioles and capillaries in diseases classified elsewhere | Other disorders of kidney and ureter, not elsewhere classified | 102 | 4.58E-36 | 3.72 (3.01, 4.54) |
| J31 | J35 | Chronic rhinitis, nasopharyngitis and pharyngitis | Chronic diseases of tonsils and adenoids | 102 | 3.68E-217 | 51.25 (39.98, 65.34) |
| E02 | E11 | Subclinical iodine-deficiency hypothyroidism | Type 2 diabetes mellitus | 101 | 7.96E-26 | 3.43 (2.71, 4.29) |
| I70 | M48 | Atherosclerosis | Other spondylopathies | 101 | 5.37E-11 | 1.99 (1.61, 2.43) |
| C34 | J18 | Malignant neoplasm of bronchus and lung | Pneumonia, organism unspecified | 100 | 3.06E-85 | 8.8 (7.04, 10.9) |
| D69 | K76 | Purpura and other haemorrhagic conditions | Other diseases of liver | 100 | 4.73E-12 | 2.12 (1.7, 2.61) |
| B16 | K74 | Acute hepatitis B | Fibrosis and cirrhosis of liver | 99 | 3.43E-134 | 15.38 (12.31, 19.01) |
| D64 | D70 | Other anaemias | Agranulocytosis | 99 | 6.12E-55 | 5.71 (4.56, 7.07) |
| E78 | F45 | Disorders of lipoprotein metabolism and other lipidaemias | Somatoform disorders | 99 | 3.86E-21 | 2.9 (2.31, 3.6) |
| I50 | J96 | Heart failure | Respiratory failure, not elsewhere classified | 99 | 3.54E-47 | 5.35 (4.24, 6.69) |
| N13 | N40 | Obstructive and reflux uropathyC | Hyperplasia of prostate | 99 | 3.64E-31 | 3.56 (2.86, 4.37) |
| E14 | N08 | Unspecified diabetes mellitus | Glomerular disorders in diseases classified elsewhere | 98 | 2.90E-65 | 6.31 (5.07, 7.75) |
| J42 | J43 | Unspecified chronic bronchitis | Emphysema | 98 | 8.88E-117 | 13.03 (10.41, 16.15) |
| N39 | N40 | Other disorders of urinary system | Hyperplasia of prostate | 98 | 1.04E-53 | 5.69 (4.55, 7.04) |
| N73 | N85 | Other female pelvic inflammatory diseases | Other noninflammatory disorders of uterus, except cervix | 98 | 3.50E-24 | 3.07 (2.46, 3.8) |
| D25 | N81 | Leiomyoma of uterus | Female genital prolapse | 97 | 2.06E-50 | 6.3 (4.93, 7.99) |
| D27 | N72 | Benign neoplasm of ovary | Inflammatory disease of cervix uteri | 97 | 1.76E-53 | 6.36 (5.01, 8.02) |
| J43 | J98 | Emphysema | Other respiratory disorders | 97 | 1.17E-53 | 5.35 (4.29, 6.58) |
| E78 | I45 | Disorders of lipoprotein metabolism and other lipidaemias | Other conduction disorders | 96 | 1.33E-10 | 2.06 (1.64, 2.56) |
| E87 | J96 | Other disorders of fluid, electrolyte and acid-base balance | Respiratory failure, not elsewhere classified | 96 | 8.56E-106 | 12.7 (10.06, 15.88) |
| G47 | J34 | Sleep disorders | Other disorders of nose and nasal sinuses | 96 | 2.38E-108 | 11.75 (9.39, 14.53) |
| G63 | K82 | Polyneuropathy in diseases classified elsewhere | Other diseases of gallbladder | 96 | 8.32E-18 | 2.49 (2.01, 3.05) |
| D25 | D27 | Leiomyoma of uterus | Benign neoplasm of ovary | 95 | 1.30E-32 | 4.18 (3.29, 5.26) |
| E11 | H43 | Type 2 diabetes mellitus | Disorders of vitreous body | 95 | 2.38E-12 | 2.26 (1.79, 2.83) |
| K25 | K26 | Gastric ulcer | Duodenal ulcer | 95 | 6.41E-99 | 10.37 (8.29, 12.81) |
| K27 | K29 | Peptic ulcer, site unspecified | Gastritis and duodenitis | 95 | 3.18E-37 | 4.58 (3.6, 5.76) |
| K76 | M81 | Other diseases of liver | Osteoporosis without pathological fracture | 95 | 7.79E-09 | 1.91 (1.53, 2.37) |
| A16 | J18 | Respiratory tuberculosis, not confirmed bacteriologically or histologically | Pneumonia, organism unspecified | 94 | 1.65E-32 | 3.64 (2.92, 4.47) |
| E87 | N28 | Other disorders of fluid, electrolyte and acid-base balance | Other disorders of kidney and ureter, not elsewhere classified | 94 | 9.07E-10 | 1.92 (1.55, 2.36) |
| G47 | I50 | Sleep disorders | Heart failure | 94 | 4.15E-13 | 2.23 (1.78, 2.75) |
| J31 | J33 | Chronic rhinitis, nasopharyngitis and pharyngitis | Nasal polyp | 94 | 2.83E-178 | 31.51 (24.73, 39.79) |
| K66 | K80 | Other disorders of peritoneum | Cholelithiasis | 94 | 1.01E-71 | 10.12 (7.83, 13) |
| E66 | K76 | Obesity | Other diseases of liver | 93 | 2.35E-50 | 7.04 (5.43, 9.07) |
| E78 | F41 | Disorders of lipoprotein metabolism and other lipidaemias | Other anxiety disorders | 93 | 2.47E-11 | 2.14 (1.7, 2.66) |
| E87 | N08 | Other disorders of fluid, electrolyte and acid-base balance | Glomerular disorders in diseases classified elsewhere | 93 | 1.34E-18 | 2.59 (2.08, 3.18) |
| D50 | K26 | Iron deficiency anaemia | Duodenal ulcer | 92 | 2.60E-101 | 11.68 (9.26, 14.55) |
| E06 | E78 | Thyroiditis | Disorders of lipoprotein metabolism and other lipidaemias | 92 | 9.85E-13 | 2.26 (1.8, 2.81) |
| E87 | J44 | Other disorders of fluid, electrolyte and acid-base balance | Other chronic obstructive pulmonary disease | 92 | 2.72E-17 | 2.51 (2.02, 3.09) |
| I44 | I50 | Atrioventricular and left bundle-branch block | Heart failure | 92 | 7.89E-49 | 6.22 (4.85, 7.9) |
| J44 | J47 | Other chronic obstructive pulmonary disease | Bronchiectasis | 92 | 6.04E-118 | 14.81 (11.72, 18.53) |
| A49 | K76 | Bacterial infection of unspecified site | Other diseases of liver | 91 | 1.81E-12 | 2.24 (1.78, 2.79) |
| K25 | K92 | Gastric ulcer | Other diseases of digestive system | 91 | 8.30E-114 | 13.27 (10.54, 16.5) |
| A59 | N77 | Trichomoniasis | Vulvovaginal ulceration and inflammation in diseases classified elsewhere | 90 | 7.19E-183 | 5129.28 (2947.65, 9428.38) |
| I79 | K82 | Disorders of arteries, arterioles and capillaries in diseases classified elsewhere | Other diseases of gallbladder | 90 | 1.09E-31 | 3.65 (2.92, 4.51) |
| K25 | K31 | Gastric ulcer | Other diseases of stomach and duodenum | 90 | 1.25E-76 | 7.99 (6.37, 9.89) |
| D64 | J98 | Other anaemias | Other respiratory disorders | 89 | 1.73E-09 | 1.95 (1.56, 2.4) |
| G55 | M50 | Nerve root and plexus compressions in diseases classified elsewhere | Cervical disc disorders | 89 | 3.41E-31 | 3.63 (2.9, 4.48) |
| G93 | I63 | Other disorders of brain | Cerebral infarction | 89 | 2.24E-09 | 2.01 (1.59, 2.51) |
| I65 | M50 | Occlusion and stenosis of precerebral arteries, not resulting in cerebral infarction | Cervical disc disorders | 89 | 1.53E-08 | 1.87 (1.5, 2.31) |
| N40 | N41 | Hyperplasia of prostate | Inflammatory diseases of prostate | 89 | 4.08E-71 | 8.45 (6.65, 10.63) |
| D50 | N85 | Iron deficiency anaemia | Other noninflammatory disorders of uterus, except cervix | 88 | 1.41E-53 | 6.14 (4.85, 7.68) |
| D64 | K92 | Other anaemias | Other diseases of digestive system | 88 | 3.22E-56 | 6.24 (4.94, 7.78) |
| E72 | G99 | Other disorders of amino-acid metabolism | Other disorders of nervous system in diseases classified elsewhere | 88 | 1.04E-08 | 1.88 (1.5, 2.32) |
| I11 | I65 | Hypertensive heart disease | Occlusion and stenosis of precerebral arteries, not resulting in cerebral infarction | 88 | 2.03E-42 | 4.76 (3.78, 5.91) |
| I42 | I49 | Cardiomyopathy | Other cardiac arrhythmias | 88 | 3.98E-82 | 10.1 (7.93, 12.73) |
| K52 | K62 | Other noninfective gastroenteritis and colitis | Other diseases of anus and rectum | 88 | 1.56E-31 | 3.66 (2.92, 4.53) |
| M50 | M89 | Cervical disc disorders | Other disorders of bone | 88 | 7.86E-65 | 7.07 (5.61, 8.81) |
| N76 | N84 | Other inflammation of vagina and vulva | Polyp of female genital tract | 88 | 2.97E-44 | 5.01 (3.97, 6.25) |
| J20 | J31 | Acute bronchitis | Chronic rhinitis, nasopharyngitis and pharyngitis | 87 | 9.54E-15 | 2.36 (1.88, 2.91) |
| K76 | N03 | Other diseases of liver | Chronic nephritic syndrome | 87 | 1.79E-09 | 2.01 (1.59, 2.51) |
| K76 | N88 | Other diseases of liver | Other noninflammatory disorders of cervix uteri | 87 | 2.73E-19 | 2.94 (2.32, 3.67) |
| E05 | H06 | Thyrotoxicosis [hyperthyroidism] | Disorders of lacrimal system and orbit in diseases classified elsewhere | 86 | 1.20E-23 | 24185.41 (5364.31, 426983.17) |
| E72 | G44 | Other disorders of amino-acid metabolism | Other headache syndromes | 86 | 6.01E-35 | 4.1 (3.25, 5.1) |
| E77 | J94 | Disorders of glycoprotein metabolism | Other pleural conditions | 86 | 7.25E-99 | 11.79 (9.31, 14.74) |
| I10 | N19 | Essential (primary) hypertension | Unspecified kidney failure | 86 | 5.75E-09 | 2.23 (1.7, 2.91) |
| J84 | K76 | Other interstitial pulmonary diseases | Other diseases of liver | 86 | 5.45E-14 | 2.47 (1.94, 3.11) |
| H25 | H52 | Senile cataract | Disorders of refraction and accommodation | 85 | 3.75E-191 | 40.33 (31.37, 51.3) |
| H90 | H93 | Conductive and sensorineural hearing loss | Other disorders of ear, not elsewhere classified | 85 | 5.55E-265 | 93.85 (72.3, 120.7) |
| J30 | J34 | Vasomotor and allergic rhinitis | Other disorders of nose and nasal sinuses | 85 | 1.15E-195 | 57.49 (43.88, 74.76) |
| K21 | K25 | Gastro-oesophageal reflux disease | Gastric ulcer | 84 | 7.18E-74 | 8.28 (6.54, 10.33) |
| K62 | K63 | Other diseases of anus and rectum | Other diseases of intestine | 84 | 7.20E-59 | 6.5 (5.14, 8.1) |
| E11 | G59 | Type 2 diabetes mellitus | Mononeuropathy in diseases classified elsewhere | 83 | 2.40E-38 | 98.9 (52.13, 212.79) |
| E72 | M48 | Other disorders of amino-acid metabolism | Other spondylopathies | 83 | 2.01E-13 | 2.31 (1.83, 2.88) |
| I70 | J43 | Atherosclerosis | Emphysema | 83 | 1.32E-08 | 1.94 (1.54, 2.43) |
| M47 | M89 | Spondylosis | Other disorders of bone | 83 | 7.91E-11 | 2.14 (1.69, 2.68) |
| E77 | J18 | Disorders of glycoprotein metabolism | Pneumonia, organism unspecified | 82 | 3.87E-35 | 4.24 (3.35, 5.3) |
| G44 | I70 | Other headache syndromes | Atherosclerosis | 82 | 4.87E-19 | 2.84 (2.24, 3.55) |
| G47 | J31 | Sleep disorders | Chronic rhinitis, nasopharyngitis and pharyngitis | 82 | 8.17E-75 | 8.67 (6.83, 10.85) |
| G99 | I67 | Other disorders of nervous system in diseases classified elsewhere | Other cerebrovascular diseases | 82 | 1.07E-06 | 1.74 (1.38, 2.16) |
| I50 | N18 | Heart failure | Chronic kidney disease | 82 | 2.71E-16 | 2.64 (2.08, 3.32) |
| I67 | M48 | Other cerebrovascular diseases | Other spondylopathies | 82 | 1.04E-14 | 2.42 (1.92, 3.01) |
| D35 | I10 | Benign neoplasm of other and unspecified endocrine glands | Essential (primary) hypertension | 81 | 6.60E-07 | 1.99 (1.51, 2.6) |
| I09 | I50 | Other rheumatic heart diseases | Heart failure | 81 | 8.99E-83 | 26.46 (18.99, 36.99) |
| N08 | N18 | Glomerular disorders in diseases classified elsewhere | Chronic kidney disease | 81 | 5.09E-93 | 11.91 (9.33, 15.01) |
| N70 | N84 | Salpingitis and oophoritis | Polyp of female genital tract | 81 | 2.72E-51 | 6.2 (4.86, 7.8) |
| A16 | A18 | Respiratory tuberculosis, not confirmed bacteriologically or histologically | Tuberculosis of other organs | 80 | 1.18E-184 | 48.73 (37.27, 63.09) |
| G99 | M50 | Other disorders of nervous system in diseases classified elsewhere | Cervical disc disorders | 80 | 1.46E-06 | 1.74 (1.37, 2.16) |
| I45 | K76 | Other conduction disorders | Other diseases of liver | 80 | 3.31E-07 | 1.86 (1.45, 2.34) |
| N28 | N39 | Other disorders of kidney and ureter, not elsewhere classified | Other disorders of urinary system | 80 | 3.62E-30 | 3.81 (3, 4.75) |
| N43 | N45 | Hydrocele and spermatocele | Orchitis and epididymitis | 80 | 7.04E-225 | 81.07 (61.63, 105.65) |
| N76 | N83 | Other inflammation of vagina and vulva | Noninflammatory disorders of ovary, fallopian tube and broad ligament | 80 | 1.21E-18 | 2.86 (2.25, 3.59) |
| I11 | I67 | Hypertensive heart disease | Other cerebrovascular diseases | 79 | 1.82E-29 | 3.82 (3, 4.78) |
| J18 | J47 | Pneumonia, organism unspecified | Bronchiectasis | 79 | 4.42E-49 | 5.94 (4.65, 7.48) |
| K22 | K31 | Other diseases of oesophagus | Other diseases of stomach and duodenum | 79 | 1.26E-138 | 24.42 (18.9, 31.17) |
| D18 | K82 | Haemangioma and lymphangioma, any site | Other diseases of gallbladder | 78 | 2.57E-42 | 5.05 (3.97, 6.33) |
| D50 | N70 | Iron deficiency anaemia | Salpingitis and oophoritis | 78 | 8.93E-39 | 5.02 (3.92, 6.34) |
| E14 | H36 | Unspecified diabetes mellitus | Retinal disorders in diseases classified elsewhere | 78 | 2.53E-59 | 7.1 (5.56, 8.92) |
| E16 | E78 | Other disorders of pancreatic internal secretion | Disorders of lipoprotein metabolism and other lipidaemias | 78 | 5.74E-28 | 4.5 (3.42, 5.86) |
| E79 | I49 | Disorders of purine and pyrimidine metabolism | Other cardiac arrhythmias | 78 | 8.52E-14 | 2.41 (1.89, 3.01) |
| I61 | J18 | Intracerebral haemorrhage | Pneumonia, organism unspecified | 78 | 1.64E-14 | 2.46 (1.94, 3.08) |
| N70 | N85 | Salpingitis and oophoritis | Other noninflammatory disorders of uterus, except cervix | 78 | 8.09E-67 | 8.83 (6.86, 11.21) |
| E72 | J32 | Other disorders of amino-acid metabolism | Chronic sinusitis | 77 | 1.79E-06 | 1.76 (1.38, 2.2) |
| I12 | N18 | Hypertensive renal disease | Chronic kidney disease | 77 | 3.59E-228 | 88.72 (67.2, 116) |
| J43 | J47 | Emphysema | Bronchiectasis | 77 | 3.37E-117 | 18.83 (14.57, 24.03) |
| K76 | K83 | Other diseases of liver | Other diseases of biliary tract | 77 | 4.08E-12 | 2.38 (1.85, 3.02) |
| E77 | J98 | Disorders of glycoprotein metabolism | Other respiratory disorders | 76 | 1.35E-46 | 5.72 (4.47, 7.2) |
| I25 | I42 | Chronic ischaemic heart disease | Cardiomyopathy | 76 | 2.32E-10 | 2.25 (1.74, 2.88) |
| J30 | J32 | Vasomotor and allergic rhinitis | Chronic sinusitis | 76 | 3.32E-148 | 35.43 (26.91, 46.19) |
| K21 | K63 | Gastro-oesophageal reflux disease | Other diseases of intestine | 76 | 4.56E-60 | 7.32 (5.72, 9.23) |
| M17 | M23 | Gonarthrosis [arthrosis of knee] | Internal derangement of knee | 76 | 7.92E-183 | 47.26 (36.16, 61.03) |
| D18 | N28 | Haemangioma and lymphangioma, any site | Other disorders of kidney and ureter, not elsewhere classified | 75 | 3.18E-41 | 5.14 (4.02, 6.48) |
| E79 | I65 | Disorders of purine and pyrimidine metabolism | Occlusion and stenosis of precerebral arteries, not resulting in cerebral infarction | 75 | 1.09E-21 | 3.2 (2.5, 4.03) |
| G55 | M48 | Nerve root and plexus compressions in diseases classified elsewhere | Other spondylopathies | 75 | 3.56E-47 | 5.74 (4.49, 7.23) |
| I11 | I49 | Hypertensive heart disease | Other cardiac arrhythmias | 75 | 1.31E-18 | 2.9 (2.27, 3.65) |
| I85 | K74 | Oesophageal varices | Fibrosis and cirrhosis of liver | 75 | 6.19E-175 | 188.17 (131.14, 271.93) |
| K26 | K31 | Duodenal ulcer | Other diseases of stomach and duodenum | 75 | 1.14E-52 | 6.43 (5.03, 8.11) |
| K31 | K52 | Other diseases of stomach and duodenum | Other noninfective gastroenteritis and colitis | 75 | 1.73E-26 | 3.58 (2.81, 4.5) |
| E72 | I66 | Other disorders of amino-acid metabolism | Occlusion and stenosis of cerebral arteries, not resulting in cerebral infarction | 74 | 2.08E-78 | 12.77 (9.72, 16.58) |
| E78 | I66 | Disorders of lipoprotein metabolism and other lipidaemias | Occlusion and stenosis of cerebral arteries, not resulting in cerebral infarction | 74 | 1.04E-16 | 3.04 (2.33, 3.93) |
| K22 | K76 | Other diseases of oesophagus | Other diseases of liver | 74 | 1.18E-13 | 2.6 (2, 3.33) |
| M47 | M81 | Spondylosis | Osteoporosis without pathological fracture | 74 | 1.81E-06 | 1.81 (1.41, 2.29) |
| A09 | K29 | Other gastroenteritis and colitis of infectious and unspecified origin | Gastritis and duodenitis | 73 | 1.77E-47 | 9.12 (6.73, 12.27) |
| E79 | I20 | Disorders of purine and pyrimidine metabolism | Angina pectoris | 73 | 2.38E-10 | 2.17 (1.69, 2.73) |
| N70 | N76 | Salpingitis and oophoritis | Other inflammation of vagina and vulva | 73 | 1.44E-41 | 5.55 (4.3, 7.06) |
| D50 | N80 | Iron deficiency anaemia | Endometriosis | 72 | 3.26E-28 | 4.1 (3.17, 5.22) |
| D64 | K74 | Other anaemias | Fibrosis and cirrhosis of liver | 72 | 4.03E-23 | 3.45 (2.68, 4.37) |
| E87 | K92 | Other disorders of fluid, electrolyte and acid-base balance | Other diseases of digestive system | 72 | 1.47E-24 | 3.53 (2.75, 4.47) |
| E87 | N39 | Other disorders of fluid, electrolyte and acid-base balance | Other disorders of urinary system | 72 | 3.39E-08 | 1.95 (1.52, 2.46) |
| H35 | H52 | Other retinal disorders | Disorders of refraction and accommodation | 72 | 1.82E-140 | 26.3 (20.25, 33.68) |
| I21 | I49 | Acute myocardial infarction | Other cardiac arrhythmias | 72 | 7.07E-15 | 2.61 (2.03, 3.3) |
| D12 | I84 | Benign neoplasm of colon, rectum, anus and anal canal | Haemorrhoids | 71 | 1.11E-81 | 21.73 (15.81, 29.74) |
| E02 | I25 | Subclinical iodine-deficiency hypothyroidism | Chronic ischaemic heart disease | 71 | 2.39E-09 | 2.21 (1.69, 2.84) |
| E05 | I50 | Thyrotoxicosis [hyperthyroidism] | Heart failure | 71 | 2.70E-13 | 2.52 (1.95, 3.21) |
| G45 | M48 | Transient cerebral ischaemic attacks and related syndromes | Other spondylopathies | 71 | 3.76E-08 | 1.96 (1.52, 2.47) |
| I84 | K59 | Haemorrhoids | Other functional intestinal disorders | 71 | 3.84E-45 | 6.69 (5.1, 8.66) |
| N73 | N77 | Other female pelvic inflammatory diseases | Vulvovaginal ulceration and inflammation in diseases classified elsewhere | 71 | 4.97E-67 | 14.58 (10.72, 19.68) |
| E03 | I70 | Other hypothyroidism | Atherosclerosis | 70 | 1.31E-15 | 2.75 (2.13, 3.5) |
| G47 | I70 | Sleep disorders | Atherosclerosis | 70 | 4.44E-14 | 2.62 (2.02, 3.32) |
| H26 | H43 | Other cataract | Disorders of vitreous body | 70 | 4.57E-144 | 31.19 (23.78, 40.33) |
| K29 | K83 | Gastritis and duodenitis | Other diseases of biliary tract | 70 | 2.31E-09 | 2.16 (1.66, 2.77) |
| N71 | N93 | Inflammatory disease of uterus, except cervix | Other abnormal uterine and vaginal bleeding | 70 | 1.19E-70 | 11.48 (8.71, 14.93) |
| A16 | J94 | Respiratory tuberculosis, not confirmed bacteriologically or histologically | Other pleural conditions | 69 | 4.80E-49 | 6.51 (5.03, 8.29) |
| D25 | N87 | Leiomyoma of uterus | Dysplasia of cervix uteri | 69 | 5.96E-25 | 4.24 (3.2, 5.55) |
| D64 | N28 | Other anaemias | Other disorders of kidney and ureter, not elsewhere classified | 69 | 1.59E-07 | 1.95 (1.51, 2.46) |
| E87 | K31 | Other disorders of fluid, electrolyte and acid-base balance | Other diseases of stomach and duodenum | 69 | 1.26E-07 | 1.92 (1.49, 2.43) |
| J44 | J45 | Other chronic obstructive pulmonary disease | Asthma | 69 | 9.95E-60 | 8.15 (6.28, 10.41) |
| J44 | J98 | Other chronic obstructive pulmonary disease | Other respiratory disorders | 69 | 8.85E-13 | 2.44 (1.9, 3.1) |
| D53 | K29 | Other nutritional anaemias | Gastritis and duodenitis | 68 | 2.76E-25 | 4.28 (3.23, 5.61) |
| D73 | K76 | Diseases of spleen | Other diseases of liver | 68 | 5.09E-24 | 4.19 (3.15, 5.5) |
| F45 | K29 | Somatoform disorders | Gastritis and duodenitis | 68 | 2.05E-07 | 1.97 (1.51, 2.52) |
| H33 | H52 | Retinal detachments and breaks | Disorders of refraction and accommodation | 68 | 1.15E-218 | 129.39 (95.22, 174.34) |
| I65 | N08 | Occlusion and stenosis of precerebral arteries, not resulting in cerebral infarction | Glomerular disorders in diseases classified elsewhere | 68 | 1.39E-09 | 2.14 (1.66, 2.72) |
| K81 | K83 | Cholecystitis | Other diseases of biliary tract | 68 | 1.16E-70 | 10.45 (8, 13.44) |
| D50 | E87 | Iron deficiency anaemia | Other disorders of fluid, electrolyte and acid-base balance | 67 | 4.48E-10 | 2.22 (1.71, 2.82) |
| D50 | N84 | Iron deficiency anaemia | Polyp of female genital tract | 67 | 8.14E-18 | 3.08 (2.37, 3.94) |
| E72 | G51 | Other disorders of amino-acid metabolism | Facial nerve disorders | 67 | 2.46E-17 | 2.96 (2.28, 3.77) |
| E87 | K85 | Other disorders of fluid, electrolyte and acid-base balance | Acute pancreatitis | 67 | 1.60E-14 | 2.64 (2.04, 3.36) |
| E87 | N18 | Other disorders of fluid, electrolyte and acid-base balance | Chronic kidney disease | 67 | 2.18E-33 | 4.73 (3.64, 6.05) |
| I12 | K76 | Hypertensive renal disease | Other diseases of liver | 67 | 1.30E-18 | 3.43 (2.59, 4.49) |
| I66 | I70 | Occlusion and stenosis of cerebral arteries, not resulting in cerebral infarction | Atherosclerosis | 67 | 6.74E-48 | 7.85 (5.91, 10.3) |
| N08 | N28 | Glomerular disorders in diseases classified elsewhere | Other disorders of kidney and ureter, not elsewhere classified | 67 | 3.99E-13 | 2.5 (1.93, 3.19) |
| N43 | N50 | Hydrocele and spermatocele | Other disorders of male genital organs | 67 | 2.27E-215 | 131.04 (96.1, 177.05) |
| E04 | I70 | Other nontoxic goitre | Atherosclerosis | 66 | 8.69E-10 | 2.21 (1.7, 2.83) |
| E78 | I44 | Disorders of lipoprotein metabolism and other lipidaemias | Atrioventricular and left bundle-branch block | 66 | 1.14E-08 | 2.21 (1.68, 2.87) |
| H43 | H52 | Disorders of vitreous body | Disorders of refraction and accommodation | 66 | 1.53E-157 | 40.44 (30.6, 52.66) |
| I05 | I50 | Rheumatic mitral valve diseases | Heart failure | 66 | 2.47E-67 | 50.4 (32.66, 79.4) |
| J81 | J94 | Pulmonary oedema | Other pleural conditions | 66 | 1.79E-140 | 45.71 (33.77, 61.22) |
| K81 | N28 | Cholecystitis | Other disorders of kidney and ureter, not elsewhere classified | 66 | 8.03E-07 | 1.87 (1.44, 2.37) |
| E14 | I79 | Unspecified diabetes mellitus | Disorders of arteries, arterioles and capillaries in diseases classified elsewhere | 65 | 2.33E-24 | 3.77 (2.89, 4.82) |
| E79 | I67 | Disorders of purine and pyrimidine metabolism | Other cerebrovascular diseases | 65 | 4.96E-13 | 2.54 (1.95, 3.24) |
| E87 | K74 | Other disorders of fluid, electrolyte and acid-base balance | Fibrosis and cirrhosis of liver | 65 | 1.08E-10 | 2.29 (1.76, 2.91) |
| I79 | M81 | Disorders of arteries, arterioles and capillaries in diseases classified elsewhere | Osteoporosis without pathological fracture | 65 | 2.98E-61 | 9.38 (7.15, 12.11) |
| I80 | I83 | Phlebitis and thrombophlebitis | Varicose veins of lower extremities | 65 | 5.13E-140 | 33.13 (25.03, 43.19) |
| B18 | C22 | Chronic viral hepatitis | Malignant neoplasm of liver and intrahepatic bile ducts | 64 | 2.53E-107 | 22.98 (17.25, 30.17) |
| E16 | K76 | Other disorders of pancreatic internal secretion | Other diseases of liver | 64 | 2.98E-20 | 3.83 (2.86, 5.07) |
| E77 | K74 | Disorders of glycoprotein metabolism | Fibrosis and cirrhosis of liver | 64 | 6.63E-62 | 9.1 (6.94, 11.71) |
| E78 | I12 | Disorders of lipoprotein metabolism and other lipidaemias | Hypertensive renal disease | 64 | 7.20E-14 | 2.9 (2.17, 3.8) |
| H34 | H35 | Retinal vascular occlusions | Other retinal disorders | 64 | 5.54E-171 | 54.63 (40.92, 71.9) |
| H66 | H90 | Suppurative and unspecified otitis media | Conductive and sensorineural hearing loss | 64 | 1.52E-140 | 32.41 (24.53, 42.15) |
| I15 | N18 | Secondary hypertension | Chronic kidney disease | 64 | 5.52E-204 | 108.22 (79.59, 145.54) |
| I27 | J96 | Other pulmonary heart diseases | Respiratory failure, not elsewhere classified | 64 | 1.15E-209 | 108.44 (80.05, 145.07) |
| K25 | K65 | Gastric ulcer | Peritonitis | 64 | 2.01E-63 | 9.26 (7.08, 11.91) |
| K31 | K92 | Other diseases of stomach and duodenum | Other diseases of digestive system | 64 | 7.23E-51 | 7.22 (5.52, 9.27) |
| K65 | K74 | Peritonitis | Fibrosis and cirrhosis of liver | 64 | 2.02E-55 | 7.99 (6.11, 10.28) |
| M47 | N91 | Spondylosis | Absent, scanty and rare menstruation | 64 | 5.79E-38 | 6.58 (4.91, 8.72) |
| D13 | K29 | Benign neoplasm of other and ill-defined parts of digestive system | Gastritis and duodenitis | 63 | 4.26E-42 | 9.63 (6.92, 13.33) |
| E72 | G47 | Other disorders of amino-acid metabolism | Sleep disorders | 63 | 8.55E-16 | 2.9 (2.22, 3.73) |
| E87 | I51 | Other disorders of fluid, electrolyte and acid-base balance | Complications and ill-defined descriptions of heart disease | 63 | 1.22E-06 | 1.87 (1.44, 2.39) |
| N18 | N28 | Chronic kidney disease | Other disorders of kidney and ureter, not elsewhere classified | 63 | 4.60E-45 | 6.62 (5.04, 8.53) |
| D27 | N83 | Benign neoplasm of ovary | Noninflammatory disorders of ovary, fallopian tube and broad ligament | 62 | 5.95E-44 | 7.24 (5.44, 9.5) |
| E78 | G43 | Disorders of lipoprotein metabolism and other lipidaemias | Migraine | 62 | 2.20E-11 | 2.57 (1.93, 3.36) |
| E87 | J43 | Other disorders of fluid, electrolyte and acid-base balance | Emphysema | 62 | 2.59E-12 | 2.53 (1.93, 3.24) |
| G63 | H26 | Polyneuropathy in diseases classified elsewhere | Other cataract | 62 | 8.73E-24 | 3.83 (2.92, 4.94) |
| N73 | N88 | Other female pelvic inflammatory diseases | Other noninflammatory disorders of cervix uteri | 62 | 3.84E-12 | 2.6 (1.97, 3.37) |
| B17 | K76 | Other acute viral hepatitis | Other diseases of liver | 61 | 2.91E-08 | 2.17 (1.63, 2.82) |
| D25 | D70 | Leiomyoma of uterus | Agranulocytosis | 61 | 1.57E-07 | 2.06 (1.56, 2.68) |
| G44 | I65 | Other headache syndromes | Occlusion and stenosis of precerebral arteries, not resulting in cerebral infarction | 61 | 2.00E-23 | 3.82 (2.91, 4.93) |
| H25 | H43 | Senile cataract | Disorders of vitreous body | 61 | 8.21E-135 | 37.2 (27.7, 49.17) |
| H26 | H40 | Other cataract | Glaucoma | 61 | 6.11E-141 | 39.53 (29.48, 52.18) |
| H35 | H43 | Other retinal disorders | Disorders of vitreous body | 61 | 3.11E-138 | 35.52 (26.62, 46.61) |
| J37 | J38 | Chronic laryngitis and laryngotracheitis | Diseases of vocal cords and larynx, not elsewhere classified | 61 | 5.96E-193 | 154.94 (110.56, 215.72) |
| J43 | J84 | Emphysema | Other interstitial pulmonary diseases | 61 | 4.99E-91 | 18.18 (13.61, 23.88) |
| D64 | E03 | Other anaemias | Other hypothyroidism | 60 | 4.84E-11 | 2.45 (1.86, 3.17) |
| E87 | I47 | Other disorders of fluid, electrolyte and acid-base balance | Paroxysmal tachycardia | 60 | 9.13E-22 | 3.64 (2.77, 4.7) |
| J18 | J45 | Pneumonia, organism unspecified | Asthma | 60 | 1.25E-16 | 3.04 (2.31, 3.92) |
| K20 | K76 | Oesophagitis | Other diseases of liver | 60 | 1.48E-10 | 2.5 (1.87, 3.29) |
| K72 | K74 | Hepatic failure, not elsewhere classified | Fibrosis and cirrhosis of liver | 60 | 1.10E-122 | 34.65 (25.6, 46.22) |
| N47 | N48 | Redundant prepuce, phimosis and paraphimosis | Other disorders of penis | 60 | 1.70E-226 | 489.17 (334.81, 713.44) |
| N70 | N93 | Salpingitis and oophoritis | Other abnormal uterine and vaginal bleeding | 60 | 3.58E-18 | 3.3 (2.5, 4.27) |
| C34 | C79 | Malignant neoplasm of bronchus and lung | Secondary malignant neoplasm of other and unspecified sites | 59 | 1.03E-197 | 163.08 (116.37, 226.36) |
| D26 | D64 | Other benign neoplasms of uterus | Other anaemias | 59 | 3.67E-23 | 4.2 (3.14, 5.51) |
| D27 | N73 | Benign neoplasm of ovary | Other female pelvic inflammatory diseases | 59 | 3.58E-22 | 4.07 (3.04, 5.36) |
| D69 | E87 | Purpura and other haemorrhagic conditions | Other disorders of fluid, electrolyte and acid-base balance | 59 | 3.13E-28 | 4.53 (3.42, 5.87) |
| H36 | N40 | Retinal disorders in diseases classified elsewhere | Hyperplasia of prostate | 59 | 2.59E-09 | 2.31 (1.74, 2.99) |
| H91 | H93 | Other hearing loss | Other disorders of ear, not elsewhere classified | 59 | 9.42E-155 | 49.25 (36.6, 65.2) |
| I51 | I65 | Complications and ill-defined descriptions of heart disease | Occlusion and stenosis of precerebral arteries, not resulting in cerebral infarction | 59 | 3.34E-08 | 2.1 (1.6, 2.71) |
| K40 | N43 | Inguinal hernia | Hydrocele and spermatocele | 59 | 1.74E-66 | 11.91 (8.9, 15.66) |
| K63 | N40 | Other diseases of intestine | Hyperplasia of prostate | 59 | 1.58E-10 | 2.42 (1.83, 3.14) |
| D69 | D70 | Purpura and other haemorrhagic conditions | Agranulocytosis | 58 | 9.06E-112 | 25.24 (18.86, 33.16) |
| E78 | K20 | Disorders of lipoprotein metabolism and other lipidaemias | Oesophagitis | 58 | 2.28E-07 | 2.12 (1.58, 2.79) |
| H26 | H36 | Other cataract | Retinal disorders in diseases classified elsewhere | 58 | 1.36E-52 | 8.41 (6.33, 10.95) |
| H35 | I70 | Other retinal disorders | Atherosclerosis | 58 | 1.24E-12 | 2.69 (2.03, 3.51) |
| J30 | J31 | Vasomotor and allergic rhinitis | Chronic rhinitis, nasopharyngitis and pharyngitis | 58 | 1.29E-104 | 25.96 (19.19, 34.58) |
| K31 | N28 | Other diseases of stomach and duodenum | Other disorders of kidney and ureter, not elsewhere classified | 58 | 2.49E-12 | 2.59 (1.96, 3.34) |
| M23 | M25 | Internal derangement of knee | Other joint disorders, not elsewhere classified | 58 | 3.51E-159 | 52.74 (39.14, 69.86) |
| D64 | D69 | Other anaemias | Purpura and other haemorrhagic conditions | 57 | 2.80E-25 | 4.41 (3.31, 5.78) |
| D64 | K26 | Other anaemias | Duodenal ulcer | 57 | 1.05E-12 | 2.68 (2.02, 3.48) |
| E05 | I43 | Thyrotoxicosis [hyperthyroidism] | Cardiomyopathy in diseases classified elsewhere | 57 | 2.07E-184 | 231.09 (159.61, 333.88) |
| A16 | K71 | Respiratory tuberculosis, not confirmed bacteriologically or histologically | Toxic liver disease | 56 | 8.16E-122 | 37.79 (27.68, 50.78) |
| B37 | N73 | Candidiasis | Other female pelvic inflammatory diseases | 56 | 2.27E-57 | 19 (13.2, 27.23) |
| D64 | J94 | Other anaemias | Other pleural conditions | 56 | 1.20E-10 | 2.45 (1.84, 3.18) |
| D64 | N08 | Other anaemias | Glomerular disorders in diseases classified elsewhere | 56 | 5.19E-08 | 2.16 (1.62, 2.8) |
| E79 | G45 | Disorders of purine and pyrimidine metabolism | Transient cerebral ischaemic attacks and related syndromes | 56 | 6.83E-08 | 2.11 (1.59, 2.73) |
| H52 | I70 | Disorders of refraction and accommodation | Atherosclerosis | 56 | 7.88E-13 | 2.77 (2.08, 3.62) |
| I27 | J43 | Other pulmonary heart diseases | Emphysema | 56 | 5.79E-105 | 29.5 (21.58, 39.69) |
| I65 | I69 | Occlusion and stenosis of precerebral arteries, not resulting in cerebral infarction | Sequelae of cerebrovascular disease | 56 | 1.87E-08 | 2.18 (1.64, 2.83) |
| K29 | K57 | Gastritis and duodenitis | Diverticular disease of intestine | 56 | 1.02E-41 | 12.83 (8.85, 18.58) |
| K51 | M07 | Ulcerative colitis | Psoriatic and enteropathic arthropathies | 56 | 1.49E-258 | 1528.91 (1007.21, 2329.78) |
| M43 | M51 | Other deforming dorsopathies | Other intervertebral disc disorders | 56 | 1.26E-42 | 9.88 (7.08, 13.67) |
| N80 | N84 | Endometriosis | Polyp of female genital tract | 56 | 4.67E-20 | 3.69 (2.76, 4.83) |
| A16 | D64 | Respiratory tuberculosis, not confirmed bacteriologically or histologically | Other anaemias | 55 | 5.69E-12 | 2.67 (2, 3.48) |
| E03 | I49 | Other hypothyroidism | Other cardiac arrhythmias | 55 | 9.86E-11 | 2.47 (1.86, 3.22) |
| E11 | I12 | Type 2 diabetes mellitus | Hypertensive renal disease | 55 | 8.33E-07 | 2.11 (1.55, 2.82) |
| G43 | M47 | Migraine | Spondylosis | 55 | 1.59E-15 | 3.24 (2.4, 4.29) |
| G44 | I67 | Other headache syndromes | Other cerebrovascular diseases | 55 | 8.40E-12 | 2.59 (1.95, 3.37) |
| H90 | J31 | Conductive and sensorineural hearing loss | Chronic rhinitis, nasopharyngitis and pharyngitis | 55 | 5.69E-52 | 8.72 (6.52, 11.42) |
| I25 | I97 | Chronic ischaemic heart disease | Postprocedural disorders of circulatory system, not elsewhere classified | 55 | 1.02E-35 | 16.04 (10.43, 24.98) |
| M50 | M75 | Cervical disc disorders | Shoulder lesions | 55 | 7.03E-39 | 6.59 (4.91, 8.67) |
| N84 | N85 | Polyp of female genital tract | Other noninflammatory disorders of uterus, except cervix | 55 | 9.27E-24 | 4.26 (3.18, 5.59) |
| N85 | N93 | Other noninflammatory disorders of uterus, except cervix | Other abnormal uterine and vaginal bleeding | 55 | 2.08E-15 | 3.16 (2.36, 4.14) |
| A16 | D70 | Respiratory tuberculosis, not confirmed bacteriologically or histologically | Agranulocytosis | 54 | 3.57E-75 | 14.65 (10.88, 19.33) |
| D18 | N40 | Haemangioma and lymphangioma, any site | Hyperplasia of prostate | 54 | 1.34E-16 | 3.33 (2.48, 4.38) |
| E03 | E06 | Other hypothyroidism | Thyroiditis | 54 | 1.17E-76 | 15.46 (11.45, 20.46) |
| E11 | E88 | Type 2 diabetes mellitus | Other metabolic disorders | 54 | 1.95E-34 | 14.36 (9.41, 22.13) |
| E55 | E78 | Vitamin D deficiency | Disorders of lipoprotein metabolism and other lipidaemias | 54 | 1.32E-26 | 6.43 (4.55, 9.02) |
| I47 | I70 | Paroxysmal tachycardia | Atherosclerosis | 54 | 2.88E-09 | 2.33 (1.74, 3.06) |
| K31 | K82 | Other diseases of stomach and duodenum | Other diseases of gallbladder | 54 | 6.69E-09 | 2.24 (1.68, 2.92) |
| N80 | N85 | Endometriosis | Other noninflammatory disorders of uterus, except cervix | 54 | 4.28E-28 | 5.02 (3.72, 6.62) |
| E87 | I21 | Other disorders of fluid, electrolyte and acid-base balance | Acute myocardial infarction | 53 | 1.68E-08 | 2.23 (1.67, 2.92) |
| H26 | H35 | Other cataract | Other retinal disorders | 53 | 5.98E-72 | 13.92 (10.32, 18.37) |
| I31 | I50 | Other diseases of pericardium | Heart failure | 53 | 3.16E-40 | 9.12 (6.53, 12.56) |
| I65 | I66 | Occlusion and stenosis of precerebral arteries, not resulting in cerebral infarction | Occlusion and stenosis of cerebral arteries, not resulting in cerebral infarction | 53 | 1.08E-53 | 10.84 (7.93, 14.55) |
| J96 | J98 | Respiratory failure, not elsewhere classified | Other respiratory disorders | 53 | 1.20E-45 | 8.14 (6.03, 10.78) |
| K21 | K22 | Gastro-oesophageal reflux disease | Other diseases of oesophagus | 53 | 4.50E-80 | 17.11 (12.62, 22.74) |
| K65 | K81 | Peritonitis | Cholecystitis | 53 | 3.22E-18 | 3.45 (2.58, 4.51) |
| K65 | K85 | Peritonitis | Acute pancreatitis | 53 | 1.31E-41 | 7.03 (5.24, 9.24) |
| K72 | K76 | Hepatic failure, not elsewhere classified | Other diseases of liver | 53 | 4.55E-12 | 2.91 (2.13, 3.9) |
| K83 | K85 | Other diseases of biliary tract | Acute pancreatitis | 53 | 7.28E-84 | 18.07 (13.34, 23.97) |
| M06 | M81 | Other rheumatoid arthritis | Osteoporosis without pathological fracture | 53 | 8.67E-81 | 17.54 (12.93, 23.31) |
| E55 | I70 | Vitamin D deficiency | Atherosclerosis | 52 | 1.53E-58 | 18.89 (13.16, 26.87) |
| G47 | J32 | Sleep disorders | Chronic sinusitis | 52 | 6.47E-26 | 4.57 (3.4, 6.01) |
| I35 | I50 | Nonrheumatic aortic valve disorders | Heart failure | 52 | 6.81E-50 | 18.7 (12.68, 27.51) |
| I48 | I70 | Atrial fibrillation and flutter | Atherosclerosis | 52 | 5.47E-11 | 2.64 (1.95, 3.49) |
| I60 | I61 | Subarachnoid haemorrhage | Intracerebral haemorrhage | 52 | 1.32E-62 | 12.07 (8.91, 16) |
| K35 | K56 | Acute appendicitis | Paralytic ileus and intestinal obstruction without hernia | 52 | 8.94E-31 | 5.31 (3.95, 6.98) |
| K82 | N08 | Other diseases of gallbladder | Glomerular disorders in diseases classified elsewhere | 52 | 3.11E-07 | 2.07 (1.54, 2.71) |
| N76 | N93 | Other inflammation of vagina and vulva | Other abnormal uterine and vaginal bleeding | 52 | 5.99E-07 | 2.07 (1.54, 2.72) |
| N83 | N88 | Noninflammatory disorders of ovary, fallopian tube and broad ligament | Other noninflammatory disorders of cervix uteri | 52 | 1.22E-17 | 3.55 (2.62, 4.69) |
| A16 | E87 | Respiratory tuberculosis, not confirmed bacteriologically or histologically | Other disorders of fluid, electrolyte and acid-base balance | 51 | 6.06E-07 | 2.05 (1.53, 2.69) |
| D50 | K74 | Iron deficiency anaemia | Fibrosis and cirrhosis of liver | 51 | 7.15E-33 | 5.93 (4.39, 7.84) |
| E53 | I63 | Deficiency of other B group vitamins | Cerebral infarction | 51 | 1.17E-34 | 11.76 (7.92, 17.42) |
| E79 | G63 | Disorders of purine and pyrimidine metabolism | Polyneuropathy in diseases classified elsewhere | 51 | 9.53E-07 | 2.03 (1.51, 2.67) |
| G63 | M81 | Polyneuropathy in diseases classified elsewhere | Osteoporosis without pathological fracture | 51 | 3.00E-24 | 4.57 (3.38, 6.04) |
| G93 | I60 | Other disorders of brain | Subarachnoid haemorrhage | 51 | 2.41E-101 | 26.18 (19.19, 34.98) |
| M46 | M51 | Other inflammatory spondylopathies | Other intervertebral disc disorders | 51 | 1.36E-34 | 8.02 (5.71, 11.12) |
| E78 | G58 | Disorders of lipoprotein metabolism and other lipidaemias | Other mononeuropathies | 50 | 1.03E-16 | 3.98 (2.84, 5.47) |
| G47 | H81 | Sleep disorders | Disorders of vestibular function | 50 | 1.10E-06 | 2.04 (1.51, 2.68) |
| I85 | K29 | Oesophageal varices | Gastritis and duodenitis | 50 | 6.30E-32 | 8.45 (5.88, 12.01) |
| J43 | J93 | Emphysema | Pneumothorax | 50 | 2.47E-99 | 32.99 (23.64, 45.22) |
| J47 | J98 | Bronchiectasis | Other respiratory disorders | 50 | 8.36E-29 | 5.23 (3.86, 6.93) |
| M17 | M65 | Gonarthrosis [arthrosis of knee] | Synovitis and tenosynovitis | 50 | 6.09E-67 | 13.9 (10.2, 18.52) |
| M19 | M65 | Other arthrosis | Synovitis and tenosynovitis | 50 | 2.21E-31 | 5.65 (4.17, 7.48) |
| A16 | B18 | Respiratory tuberculosis, not confirmed bacteriologically or histologically | Chronic viral hepatitis | 49 | 1.96E-18 | 3.66 (2.7, 4.84) |
| B18 | N08 | Chronic viral hepatitis | Glomerular disorders in diseases classified elsewhere | 49 | 2.16E-11 | 2.69 (1.99, 3.54) |
| D64 | I15 | Other anaemias | Secondary hypertension | 49 | 4.95E-54 | 13.71 (9.75, 18.95) |
| D64 | N71 | Other anaemias | Inflammatory disease of uterus, except cervix | 49 | 4.73E-17 | 3.75 (2.73, 5.04) |
| E11 | H28 | Type 2 diabetes mellitus | Cataract and other disorders of lens in diseases classified elsewhere | 49 | 2.54E-27 | 53.55 (27.36, 117.6) |
| E78 | E83 | Disorders of lipoprotein metabolism and other lipidaemias | Disorders of mineral metabolism | 49 | 4.62E-07 | 2.21 (1.6, 2.98) |
| E79 | N08 | Disorders of purine and pyrimidine metabolism | Glomerular disorders in diseases classified elsewhere | 49 | 1.40E-14 | 3.13 (2.31, 4.13) |
| G63 | H52 | Polyneuropathy in diseases classified elsewhere | Disorders of refraction and accommodation | 49 | 5.22E-22 | 4.27 (3.14, 5.66) |
| H65 | J31 | Nonsuppurative otitis media | Chronic rhinitis, nasopharyngitis and pharyngitis | 49 | 8.67E-69 | 15.3 (11.14, 20.54) |
| D25 | N77 | Leiomyoma of uterus | Vulvovaginal ulceration and inflammation in diseases classified elsewhere | 48 | 1.68E-18 | 4.43 (3.15, 6.12) |
| D64 | I12 | Other anaemias | Hypertensive renal disease | 48 | 1.88E-56 | 13.71 (9.81, 18.76) |
| D70 | N72 | Agranulocytosis | Inflammatory disease of cervix uteri | 48 | 3.15E-08 | 2.34 (1.71, 3.13) |
| D86 | K76 | Sarcoidosis | Other diseases of liver | 48 | 1.41E-11 | 2.99 (2.15, 4.08) |
| E03 | E72 | Other hypothyroidism | Other disorders of amino-acid metabolism | 48 | 1.83E-12 | 2.88 (2.12, 3.83) |
| E11 | H10 | Type 2 diabetes mellitus | Conjunctivitis | 48 | 6.28E-08 | 2.42 (1.74, 3.3) |
| E87 | K56 | Other disorders of fluid, electrolyte and acid-base balance | Paralytic ileus and intestinal obstruction without hernia | 48 | 5.15E-10 | 2.52 (1.86, 3.34) |
| F41 | G45 | Other anxiety disorders | Transient cerebral ischaemic attacks and related syndromes | 48 | 3.66E-21 | 4.2 (3.08, 5.59) |
| H60 | H66 | Otitis externa | Suppurative and unspecified otitis media | 48 | 2.20E-160 | 110.09 (77.58, 153.79) |
| H81 | H90 | Disorders of vestibular function | Conductive and sensorineural hearing loss | 48 | 1.54E-11 | 2.77 (2.03, 3.68) |
| I12 | I50 | Hypertensive renal disease | Heart failure | 48 | 6.84E-18 | 4.01 (2.89, 5.44) |
| K26 | K82 | Duodenal ulcer | Other diseases of gallbladder | 48 | 1.30E-09 | 2.46 (1.81, 3.25) |
| K63 | K82 | Other diseases of intestine | Other diseases of gallbladder | 48 | 9.11E-12 | 2.75 (2.03, 3.64) |
| N72 | N77 | Inflammatory disease of cervix uteri | Vulvovaginal ulceration and inflammation in diseases classified elsewhere | 48 | 3.11E-28 | 6.52 (4.63, 9.03) |
| B18 | K92 | Chronic viral hepatitis | Other diseases of digestive system | 47 | 8.54E-22 | 4.27 (3.13, 5.69) |
| C34 | J94 | Malignant neoplasm of bronchus and lung | Other pleural conditions | 47 | 8.74E-47 | 9.33 (6.79, 12.51) |
| E70 | I10 | Disorders of aromatic amino-acid metabolism | Essential (primary) hypertension | 47 | 4.87E-12 | 4.69 (3.03, 7.31) |
| G40 | I69 | Epilepsy | Sequelae of cerebrovascular disease | 47 | 9.04E-50 | 10.04 (7.31, 13.47) |
| G93 | J69 | Other disorders of brain | Pneumonitis due to solids and liquids | 47 | 2.06E-142 | 80.22 (56.65, 111.6) |
| H34 | H43 | Retinal vascular occlusions | Disorders of vitreous body | 47 | 1.16E-136 | 60.61 (43.4, 82.92) |
| K20 | K31 | Oesophagitis | Other diseases of stomach and duodenum | 47 | 8.13E-70 | 16.79 (12.14, 22.72) |
| N73 | N86 | Other female pelvic inflammatory diseases | Erosion and ectropion of cervix uteri | 47 | 2.03E-32 | 7.99 (5.62, 11.18) |
| D50 | K25 | Iron deficiency anaemia | Gastric ulcer | 46 | 6.55E-25 | 4.91 (3.58, 6.57) |
| D70 | E87 | Agranulocytosis | Other disorders of fluid, electrolyte and acid-base balance | 46 | 2.09E-15 | 3.37 (2.46, 4.51) |
| E02 | I50 | Subclinical iodine-deficiency hypothyroidism | Heart failure | 46 | 1.14E-08 | 2.47 (1.79, 3.34) |
| E05 | E06 | Thyrotoxicosis [hyperthyroidism] | Thyroiditis | 46 | 5.69E-76 | 18.73 (13.55, 25.29) |
| E53 | I10 | Deficiency of other B group vitamins | Essential (primary) hypertension | 46 | 2.21E-07 | 2.8 (1.89, 4.12) |
| E77 | K92 | Disorders of glycoprotein metabolism | Other diseases of digestive system | 46 | 7.06E-44 | 8.63 (6.28, 11.55) |
| E79 | N28 | Disorders of purine and pyrimidine metabolism | Other disorders of kidney and ureter, not elsewhere classified | 46 | 3.06E-07 | 2.18 (1.59, 2.9) |
| H36 | H43 | Retinal disorders in diseases classified elsewhere | Disorders of vitreous body | 46 | 6.04E-62 | 14.04 (10.15, 18.97) |
| H36 | N28 | Retinal disorders in diseases classified elsewhere | Other disorders of kidney and ureter, not elsewhere classified | 46 | 5.06E-09 | 2.43 (1.78, 3.24) |
| I09 | I48 | Other rheumatic heart diseases | Atrial fibrillation and flutter | 46 | 9.03E-157 | 152.63 (104.74, 219.48) |
| I20 | I45 | Angina pectoris | Other conduction disorders | 46 | 1.34E-15 | 3.48 (2.53, 4.67) |
| I20 | I48 | Angina pectoris | Atrial fibrillation and flutter | 46 | 6.91E-13 | 3.07 (2.23, 4.11) |
| J43 | J94 | Emphysema | Other pleural conditions | 46 | 7.13E-20 | 4.06 (2.96, 5.42) |
| J43 | J96 | Emphysema | Respiratory failure, not elsewhere classified | 46 | 2.69E-60 | 13.93 (10.04, 18.88) |
| J43 | N28 | Emphysema | Other disorders of kidney and ureter, not elsewhere classified | 46 | 2.22E-08 | 2.35 (1.72, 3.13) |
| J94 | K74 | Other pleural conditions | Fibrosis and cirrhosis of liver | 46 | 7.89E-17 | 3.56 (2.6, 4.75) |
| K21 | K82 | Gastro-oesophageal reflux disease | Other diseases of gallbladder | 46 | 2.11E-07 | 2.19 (1.6, 2.91) |
| N41 | N43 | Inflammatory diseases of prostate | Hydrocele and spermatocele | 46 | 8.36E-87 | 25.24 (18.11, 34.42) |
| N60 | N62 | Benign mammary dysplasia | Hypertrophy of breast | 46 | 2.63E-50 | 10.98 (7.91, 14.87) |
| A49 | K31 | Bacterial infection of unspecified site | Other diseases of stomach and duodenum | 45 | 1.87E-39 | 7.85 (5.69, 10.55) |
| D27 | N70 | Benign neoplasm of ovary | Salpingitis and oophoritis | 45 | 2.01E-46 | 10.39 (7.46, 14.14) |
| E02 | I70 | Subclinical iodine-deficiency hypothyroidism | Atherosclerosis | 45 | 1.90E-18 | 4.12 (2.97, 5.59) |
| E04 | I79 | Other nontoxic goitre | Disorders of arteries, arterioles and capillaries in diseases classified elsewhere | 45 | 3.68E-22 | 4.54 (3.3, 6.08) |
| E11 | K05 | Type 2 diabetes mellitus | Gingivitis and periodontal diseases | 45 | 1.16E-06 | 2.26 (1.61, 3.12) |
| E27 | I10 | Other disorders of adrenal gland | Essential (primary) hypertension | 45 | 8.63E-14 | 5.27 (3.41, 8.18) |
| E55 | N08 | Vitamin D deficiency | Glomerular disorders in diseases classified elsewhere | 45 | 1.33E-93 | 45.36 (31.26, 64.88) |
| E72 | G40 | Other disorders of amino-acid metabolism | Epilepsy | 45 | 4.27E-13 | 3.1 (2.25, 4.15) |
| G43 | I63 | Migraine | Cerebral infarction | 45 | 2.25E-07 | 2.34 (1.68, 3.2) |
| H65 | H90 | Nonsuppurative otitis media | Conductive and sensorineural hearing loss | 45 | 1.41E-114 | 42.22 (30.21, 57.67) |
| J03 | J20 | Acute tonsillitis | Acute bronchitis | 45 | 6.31E-22 | 4.51 (3.27, 6.07) |
| K59 | K62 | Other functional intestinal disorders | Other diseases of anus and rectum | 45 | 4.07E-57 | 13.09 (9.42, 17.76) |
| B49 | K29 | Unspecified mycosis | Gastritis and duodenitis | 44 | 4.68E-11 | 3.01 (2.14, 4.14) |
| C34 | C78 | Malignant neoplasm of bronchus and lung | Secondary malignant neoplasm of respiratory and digestive organs | 44 | 5.17E-129 | 73.53 (51.37, 103.21) |
| E55 | K76 | Vitamin D deficiency | Other diseases of liver | 44 | 2.72E-20 | 5.43 (3.76, 7.72) |
| E87 | G40 | Other disorders of fluid, electrolyte and acid-base balance | Epilepsy | 44 | 1.97E-14 | 3.32 (2.4, 4.46) |
| G45 | G47 | Transient cerebral ischaemic attacks and related syndromes | Sleep disorders | 44 | 2.71E-08 | 2.37 (1.72, 3.17) |
| G93 | J98 | Other disorders of brain | Other respiratory disorders | 44 | 5.67E-21 | 4.38 (3.17, 5.89) |
| G95 | M51 | Other diseases of spinal cord | Other intervertebral disc disorders | 44 | 9.71E-30 | 7.8 (5.42, 11.05) |
| H65 | J32 | Nonsuppurative otitis media | Chronic sinusitis | 44 | 5.90E-55 | 12.74 (9.14, 17.34) |
| H90 | H91 | Conductive and sensorineural hearing loss | Other hearing loss | 44 | 1.78E-71 | 17.59 (12.67, 23.81) |
| I50 | I97 | Heart failure | Postprocedural disorders of circulatory system, not elsewhere classified | 44 | 1.81E-39 | 17.39 (11.35, 26.62) |
| I60 | I67 | Subarachnoid haemorrhage | Other cerebrovascular diseases | 44 | 2.49E-21 | 4.5 (3.25, 6.07) |
| K29 | K51 | Gastritis and duodenitis | Ulcerative colitis | 44 | 9.34E-16 | 3.99 (2.81, 5.55) |
| N71 | N84 | Inflammatory disease of uterus, except cervix | Polyp of female genital tract | 44 | 8.52E-39 | 8.52 (6.09, 11.63) |
| A15 | A16 | Respiratory tuberculosis, bacteriologically and histologically confirmed | Respiratory tuberculosis, not confirmed bacteriologically or histologically | 43 | 7.41E-95 | 39.94 (27.85, 56.15) |
| A16 | E79 | Respiratory tuberculosis, not confirmed bacteriologically or histologically | Disorders of purine and pyrimidine metabolism | 43 | 1.17E-19 | 4.22 (3.05, 5.68) |
| D06 | D25 | Carcinoma in situ of cervix uteri | Leiomyoma of uterus | 43 | 1.72E-12 | 3.4 (2.39, 4.72) |
| D26 | N83 | Other benign neoplasms of uterus | Noninflammatory disorders of ovary, fallopian tube and broad ligament | 43 | 1.32E-21 | 4.83 (3.45, 6.59) |
| D64 | N03 | Other anaemias | Chronic nephritic syndrome | 43 | 4.60E-13 | 3.23 (2.32, 4.37) |
| E02 | I79 | Subclinical iodine-deficiency hypothyroidism | Disorders of arteries, arterioles and capillaries in diseases classified elsewhere | 43 | 2.11E-50 | 11.94 (8.52, 16.32) |
| E06 | J06 | Thyroiditis | Acute upper respiratory infections of multiple and unspecified sites | 43 | 8.80E-18 | 3.93 (2.83, 5.3) |
| E77 | E83 | Disorders of glycoprotein metabolism | Disorders of mineral metabolism | 43 | 4.73E-97 | 34.58 (24.5, 47.68) |
| G81 | I10 | Hemiplegia | Essential (primary) hypertension | 43 | 2.63E-15 | 11.86 (6.59, 22.68) |
| H26 | I79 | Other cataract | Disorders of arteries, arterioles and capillaries in diseases classified elsewhere | 43 | 5.18E-18 | 3.95 (2.85, 5.32) |
| H33 | H43 | Retinal detachments and breaks | Disorders of vitreous body | 43 | 2.55E-150 | 110.72 (76.95, 156.23) |
| I42 | J98 | Cardiomyopathy | Other respiratory disorders | 43 | 1.87E-31 | 6.62 (4.75, 8.98) |
| I45 | I47 | Other conduction disorders | Paroxysmal tachycardia | 43 | 6.86E-69 | 17.44 (12.5, 23.71) |
| K21 | K92 | Gastro-oesophageal reflux disease | Other diseases of digestive system | 43 | 1.18E-22 | 4.72 (3.41, 6.35) |
| K29 | K58 | Gastritis and duodenitis | Irritable bowel syndrome | 43 | 3.27E-21 | 5.63 (3.9, 8) |
| K59 | K63 | Other functional intestinal disorders | Other diseases of intestine | 43 | 1.74E-76 | 21.91 (15.6, 30.03) |
| M06 | M19 | Other rheumatoid arthritis | Other arthrosis | 43 | 3.91E-22 | 4.68 (3.37, 6.32) |
| M23 | M65 | Internal derangement of knee | Synovitis and tenosynovitis | 43 | 4.46E-87 | 26.2 (18.71, 35.78) |
| A16 | E14 | Respiratory tuberculosis, not confirmed bacteriologically or histologically | Unspecified diabetes mellitus | 42 | 3.66E-18 | 4.01 (2.89, 5.42) |
| B16 | C22 | Acute hepatitis B | Malignant neoplasm of liver and intrahepatic bile ducts | 42 | 3.63E-87 | 28.94 (20.46, 39.93) |
| C34 | J98 | Malignant neoplasm of bronchus and lung | Other respiratory disorders | 42 | 4.12E-21 | 4.61 (3.31, 6.26) |
| D50 | E77 | Iron deficiency anaemia | Disorders of glycoprotein metabolism | 42 | 1.54E-30 | 6.57 (4.71, 8.93) |
| D69 | N08 | Purpura and other haemorrhagic conditions | Glomerular disorders in diseases classified elsewhere | 42 | 3.74E-31 | 6.56 (4.7, 8.89) |
| E03 | I65 | Other hypothyroidism | Occlusion and stenosis of precerebral arteries, not resulting in cerebral infarction | 42 | 8.10E-12 | 2.99 (2.15, 4.04) |
| E87 | N19 | Other disorders of fluid, electrolyte and acid-base balance | Unspecified kidney failure | 42 | 8.27E-42 | 10.13 (7.15, 14.01) |
| H36 | K82 | Retinal disorders in diseases classified elsewhere | Other diseases of gallbladder | 42 | 1.91E-08 | 2.44 (1.76, 3.28) |
| I45 | I70 | Other conduction disorders | Atherosclerosis | 42 | 5.50E-08 | 2.43 (1.74, 3.3) |
| J30 | J45 | Vasomotor and allergic rhinitis | Asthma | 42 | 8.37E-98 | 36.42 (25.7, 50.39) |
| K21 | K52 | Gastro-oesophageal reflux disease | Other noninfective gastroenteritis and colitis | 42 | 9.38E-08 | 2.32 (1.68, 3.12) |
| K27 | K92 | Peptic ulcer, site unspecified | Other diseases of digestive system | 42 | 3.05E-70 | 19.6 (13.91, 26.93) |
| M17 | M25 | Gonarthrosis [arthrosis of knee] | Other joint disorders, not elsewhere classified | 42 | 9.84E-68 | 17.97 (12.8, 24.57) |
| N30 | N40 | Cystitis | Hyperplasia of prostate | 42 | 1.43E-39 | 10.65 (7.42, 14.98) |
| A16 | J43 | Respiratory tuberculosis, not confirmed bacteriologically or histologically | Emphysema | 41 | 7.42E-23 | 4.97 (3.55, 6.74) |
| A18 | K67 | Tuberculosis of other organs | Disorders of peritoneum in infectious diseases classified elsewhere | 41 | 3.41E-71 | 5122.97 (2198.84, 14949.61) |
| A49 | K26 | Bacterial infection of unspecified site | Duodenal ulcer | 41 | 3.44E-44 | 9.99 (7.12, 13.63) |
| E05 | I49 | Thyrotoxicosis [hyperthyroidism] | Other cardiac arrhythmias | 41 | 6.26E-09 | 2.55 (1.83, 3.46) |
| E78 | G62 | Disorders of lipoprotein metabolism and other lipidaemias | Other polyneuropathies | 41 | 2.08E-12 | 3.57 (2.47, 5.04) |
| H65 | J34 | Nonsuppurative otitis media | Other disorders of nose and nasal sinuses | 41 | 8.70E-60 | 15.91 (11.25, 21.91) |
| H66 | J32 | Suppurative and unspecified otitis media | Chronic sinusitis | 41 | 1.44E-27 | 5.89 (4.21, 8) |
| I05 | I48 | Rheumatic mitral valve diseases | Atrial fibrillation and flutter | 41 | 1.00E-140 | 260.59 (168.74, 399.89) |
| I60 | I62 | Subarachnoid haemorrhage | Other nontraumatic intracranial haemorrhage | 41 | 2.71E-124 | 73.21 (50.74, 103.34) |
| I61 | J94 | Intracerebral haemorrhage | Other pleural conditions | 41 | 1.38E-12 | 3.13 (2.24, 4.23) |
| J31 | K21 | Chronic rhinitis, nasopharyngitis and pharyngitis | Gastro-oesophageal reflux disease | 41 | 6.68E-10 | 2.69 (1.93, 3.63) |
| N76 | N80 | Other inflammation of vagina and vulva | Endometriosis | 41 | 3.59E-09 | 2.63 (1.88, 3.58) |
| B17 | K74 | Other acute viral hepatitis | Fibrosis and cirrhosis of liver | 40 | 9.39E-54 | 13.71 (9.69, 18.87) |
| B19 | K76 | Unspecified viral hepatitis | Other diseases of liver | 40 | 1.53E-10 | 3.14 (2.18, 4.41) |
| D69 | J06 | Purpura and other haemorrhagic conditions | Acute upper respiratory infections of multiple and unspecified sites | 40 | 1.43E-12 | 3.18 (2.27, 4.33) |
| D73 | K92 | Diseases of spleen | Other diseases of digestive system | 40 | 7.17E-82 | 29.19 (20.39, 40.74) |
| E87 | G93 | Other disorders of fluid, electrolyte and acid-base balance | Other disorders of brain | 40 | 4.03E-11 | 2.95 (2.1, 4.01) |
| G47 | I65 | Sleep disorders | Occlusion and stenosis of precerebral arteries, not resulting in cerebral infarction | 40 | 5.39E-09 | 2.62 (1.87, 3.56) |
| H25 | H35 | Senile cataract | Other retinal disorders | 40 | 1.04E-56 | 14.76 (10.44, 20.31) |
| I05 | I09 | Rheumatic mitral valve diseases | Other rheumatic heart diseases | 40 | 1.39E-204 | 1094.82 (695.67, 1712.49) |
| I31 | J94 | Other diseases of pericardium | Other pleural conditions | 40 | 4.37E-82 | 33.09 (22.87, 46.82) |
| N04 | N05 | Nephrotic syndrome | Unspecified nephritic syndrome | 40 | 2.27E-177 | 351.45 (232.73, 523.82) |
| A09 | K52 | Other gastroenteritis and colitis of infectious and unspecified origin | Other noninfective gastroenteritis and colitis | 39 | 1.67E-66 | 23.21 (16.02, 32.84) |
| D26 | N73 | Other benign neoplasms of uterus | Other female pelvic inflammatory diseases | 39 | 3.63E-08 | 2.59 (1.82, 3.58) |
| E03 | E04 | Other hypothyroidism | Other nontoxic goitre | 39 | 1.94E-21 | 4.88 (3.46, 6.68) |
| E04 | E05 | Other nontoxic goitre | Thyrotoxicosis [hyperthyroidism] | 39 | 3.20E-34 | 7.79 (5.51, 10.68) |
| E06 | I70 | Thyroiditis | Atherosclerosis | 39 | 3.64E-11 | 3.06 (2.16, 4.2) |
| F41 | G47 | Other anxiety disorders | Sleep disorders | 39 | 1.10E-57 | 15.26 (10.76, 21.01) |
| F45 | I70 | Somatoform disorders | Atherosclerosis | 39 | 3.63E-13 | 3.45 (2.44, 4.75) |
| H11 | H52 | Other disorders of conjunctiva | Disorders of refraction and accommodation | 39 | 4.91E-50 | 12.24 (8.66, 16.79) |
| H26 | H33 | Other cataract | Retinal detachments and breaks | 39 | 7.34E-97 | 45.9 (31.65, 65.01) |
| H40 | H47 | Glaucoma | Other disorders of optic [2nd] nerve and visual pathways | 39 | 2.48E-143 | 111.54 (76.66, 158.53) |
| I11 | I12 | Hypertensive heart disease | Hypertensive renal disease | 39 | 3.48E-72 | 23.87 (16.65, 33.32) |
| I43 | I50 | Cardiomyopathy in diseases classified elsewhere | Heart failure | 39 | 2.68E-33 | 11.2 (7.49, 16.47) |
| M19 | M81 | Other arthrosis | Osteoporosis without pathological fracture | 39 | 2.60E-17 | 4.11 (2.91, 5.62) |
| N76 | N85 | Other inflammation of vagina and vulva | Other noninflammatory disorders of uterus, except cervix | 39 | 1.31E-10 | 2.93 (2.08, 4.01) |
| B37 | N72 | Candidiasis | Inflammatory disease of cervix uteri | 38 | 3.62E-26 | 8.15 (5.48, 11.9) |
| E02 | G63 | Subclinical iodine-deficiency hypothyroidism | Polyneuropathy in diseases classified elsewhere | 38 | 1.98E-27 | 6.6 (4.63, 9.14) |
| E23 | E78 | Hypofunction and other disorders of pituitary gland | Disorders of lipoprotein metabolism and other lipidaemias | 38 | 1.03E-14 | 4.37 (2.97, 6.29) |
| E79 | I11 | Disorders of purine and pyrimidine metabolism | Hypertensive heart disease | 38 | 5.01E-17 | 4.1 (2.9, 5.62) |
| E79 | I79 | Disorders of purine and pyrimidine metabolism | Disorders of arteries, arterioles and capillaries in diseases classified elsewhere | 38 | 8.20E-07 | 2.28 (1.61, 3.11) |
| E86 | K52 | Volume depletion | Other noninfective gastroenteritis and colitis | 38 | 1.96E-63 | 265.64 (142.87, 531.54) |
| G99 | I11 | Other disorders of nervous system in diseases classified elsewhere | Hypertensive heart disease | 38 | 4.61E-09 | 2.66 (1.88, 3.63) |
| H35 | H36 | Other retinal disorders | Retinal disorders in diseases classified elsewhere | 38 | 2.91E-31 | 7.22 (5.09, 9.92) |
| I10 | I72 | Essential (primary) hypertension | Other aneurysm and dissection | 38 | 1.06E-06 | 2.92 (1.89, 4.49) |
| J93 | J94 | Pneumothorax | Other pleural conditions | 38 | 2.66E-59 | 18.4 (12.76, 25.84) |
| K22 | K26 | Other diseases of oesophagus | Duodenal ulcer | 38 | 1.42E-48 | 12.68 (8.89, 17.58) |
| K76 | L50 | Other diseases of liver | Urticaria | 38 | 3.55E-08 | 2.68 (1.86, 3.76) |
| M25 | M65 | Other joint disorders, not elsewhere classified | Synovitis and tenosynovitis | 38 | 1.38E-63 | 18.21 (12.79, 25.18) |
| M48 | M54 | Other spondylopathies | Dorsalgia | 38 | 1.82E-07 | 2.37 (1.68, 3.23) |
| M51 | M53 | Other intervertebral disc disorders | Other dorsopathies, not elsewhere classified | 38 | 2.43E-15 | 4.2 (2.9, 5.93) |
| N03 | N28 | Chronic nephritic syndrome | Other disorders of kidney and ureter, not elsewhere classified | 38 | 3.89E-21 | 4.95 (3.49, 6.8) |
| N39 | N81 | Other disorders of urinary system | Female genital prolapse | 38 | 2.16E-35 | 8.71 (6.1, 12.1) |
| N72 | N75 | Inflammatory disease of cervix uteri | Diseases of Bartholin gland | 38 | 3.64E-08 | 2.62 (1.83, 3.65) |
| D27 | D64 | Benign neoplasm of ovary | Other anaemias | 37 | 9.83E-07 | 2.37 (1.65, 3.29) |
| D62 | K92 | Acute posthaemorrhagic anaemia | Other diseases of digestive system | 37 | 1.87E-99 | 65.28 (43.83, 95.19) |
| E04 | E06 | Other nontoxic goitre | Thyroiditis | 37 | 1.54E-34 | 8.4 (5.88, 11.63) |
| E87 | J69 | Other disorders of fluid, electrolyte and acid-base balance | Pneumonitis due to solids and liquids | 37 | 9.06E-39 | 10.85 (7.47, 15.34) |
| H26 | N08 | Other cataract | Glomerular disorders in diseases classified elsewhere | 37 | 3.26E-14 | 3.63 (2.56, 4.99) |
| H52 | I79 | Disorders of refraction and accommodation | Disorders of arteries, arterioles and capillaries in diseases classified elsewhere | 37 | 2.75E-20 | 4.89 (3.44, 6.74) |
| J84 | J98 | Other interstitial pulmonary diseases | Other respiratory disorders | 37 | 3.73E-21 | 5.11 (3.58, 7.07) |
| K25 | K52 | Gastric ulcer | Other noninfective gastroenteritis and colitis | 37 | 2.23E-07 | 2.39 (1.68, 3.27) |
| K52 | M07 | Other noninfective gastroenteritis and colitis | Psoriatic and enteropathic arthropathies | 37 | 3.58E-76 | 44.08 (29.22, 65.44) |
| K62 | L30 | Other diseases of anus and rectum | Other dermatitis | 37 | 3.08E-31 | 7.49 (5.24, 10.36) |
| K76 | N42 | Other diseases of liver | Other disorders of prostate | 37 | 5.96E-13 | 4.04 (2.73, 5.86) |
| M32 | N08 | Systemic lupus erythematosus | Glomerular disorders in diseases classified elsewhere | 37 | 4.21E-102 | 110.34 (71.34, 168.68) |
| N41 | N50 | Inflammatory diseases of prostate | Other disorders of male genital organs | 37 | 2.70E-80 | 33.72 (23.12, 47.91) |
| N62 | N64 | Hypertrophy of breast | Other disorders of breast | 37 | 1.31E-73 | 32.65 (22.13, 47.05) |
| N72 | N92 | Inflammatory disease of cervix uteri | Excessive, frequent and irregular menstruation | 37 | 1.37E-15 | 4.38 (3, 6.22) |
| A18 | M49 | Tuberculosis of other organs | Spondylopathies in diseases classified elsewhere | 36 | 7.32E-95 | 2564.27 (1272.36, 5739) |
| B00 | H19 | Herpesviral [herpes simplex] infections | Disorders of sclera and cornea in diseases classified elsewhere | 36 | 4.21E-185 | 2551.61 (1505.63, 4360.82) |
| D64 | E83 | Other anaemias | Disorders of mineral metabolism | 36 | 1.60E-29 | 8.07 (5.53, 11.43) |
| D64 | N19 | Other anaemias | Unspecified kidney failure | 36 | 8.61E-40 | 12.26 (8.36, 17.5) |
| I42 | I48 | Cardiomyopathy | Atrial fibrillation and flutter | 36 | 3.16E-71 | 25.18 (17.39, 35.38) |
| I84 | L29 | Haemorrhoids | Pruritus | 36 | 1.04E-39 | 18.69 (12.02, 28.79) |
| K29 | K70 | Gastritis and duodenitis | Alcoholic liver disease | 36 | 8.38E-10 | 3.12 (2.14, 4.44) |
| K52 | K56 | Other noninfective gastroenteritis and colitis | Paralytic ileus and intestinal obstruction without hernia | 36 | 1.91E-11 | 3.15 (2.21, 4.34) |
| N84 | N88 | Polyp of female genital tract | Other noninflammatory disorders of cervix uteri | 36 | 1.68E-13 | 3.62 (2.53, 5.03) |
| B18 | K26 | Chronic viral hepatitis | Duodenal ulcer | 35 | 1.40E-06 | 2.3 (1.61, 3.18) |
| C78 | C79 | Secondary malignant neoplasm of respiratory and digestive organs | Secondary malignant neoplasm of other and unspecified sites | 35 | 1.26E-156 | 227.32 (150.66, 335.15) |
| E04 | K82 | Other nontoxic goitre | Other diseases of gallbladder | 35 | 7.56E-07 | 2.35 (1.64, 3.25) |
| E04 | N40 | Other nontoxic goitre | Hyperplasia of prostate | 35 | 5.12E-12 | 3.45 (2.38, 4.84) |
| E11 | I43 | Type 2 diabetes mellitus | Cardiomyopathy in diseases classified elsewhere | 35 | 1.00E-13 | 4.64 (3.06, 6.88) |
| F41 | I67 | Other anxiety disorders | Other cerebrovascular diseases | 35 | 3.15E-11 | 3.21 (2.23, 4.45) |
| G37 | I63 | Other demyelinating diseases of central nervous system | Cerebral infarction | 35 | 2.67E-24 | 10.83 (6.82, 17.04) |
| I38 | I50 | Endocarditis, valve unspecified | Heart failure | 35 | 1.17E-29 | 13.07 (8.33, 20.32) |
| I47 | I65 | Paroxysmal tachycardia | Occlusion and stenosis of precerebral arteries, not resulting in cerebral infarction | 35 | 1.24E-08 | 2.7 (1.88, 3.75) |
| N20 | N21 | Calculus of kidney and ureter | Calculus of lower urinary tract | 35 | 3.48E-28 | 8.01 (5.45, 11.46) |
| N41 | N45 | Inflammatory diseases of prostate | Orchitis and epididymitis | 35 | 1.06E-52 | 16.2 (11.15, 22.83) |
| N45 | N50 | Orchitis and epididymitis | Other disorders of male genital organs | 35 | 6.65E-92 | 48.42 (32.82, 69.5) |
| N75 | N76 | Diseases of Bartholin gland | Other inflammation of vagina and vulva | 35 | 2.75E-28 | 7.5 (5.16, 10.57) |
| B18 | D73 | Chronic viral hepatitis | Diseases of spleen | 34 | 8.33E-45 | 13.57 (9.27, 19.24) |
| B18 | I98 | Chronic viral hepatitis | Other disorders of circulatory system in diseases classified elsewhere | 34 | 4.94E-68 | 34.61 (22.93, 51.02) |
| C50 | N62 | Malignant neoplasm of breast | Hypertrophy of breast | 34 | 2.17E-26 | 6.89 (4.74, 9.68) |
| D26 | N70 | Other benign neoplasms of uterus | Salpingitis and oophoritis | 34 | 1.01E-28 | 7.69 (5.28, 10.85) |
| E03 | G63 | Other hypothyroidism | Polyneuropathy in diseases classified elsewhere | 34 | 3.87E-07 | 2.46 (1.71, 3.42) |
| E77 | N08 | Disorders of glycoprotein metabolism | Glomerular disorders in diseases classified elsewhere | 34 | 3.09E-12 | 3.44 (2.39, 4.79) |
| E87 | I27 | Other disorders of fluid, electrolyte and acid-base balance | Other pulmonary heart diseases | 34 | 4.04E-22 | 5.87 (4.03, 8.27) |
| E88 | H28 | Other metabolic disorders | Cataract and other disorders of lens in diseases classified elsewhere | 34 | 2.63E-169 | 4324.66 (2410.54, 7898.81) |
| G31 | I70 | Other degenerative diseases of nervous system, not elsewhere classified | Atherosclerosis | 34 | 1.75E-08 | 2.87 (1.96, 4.06) |
| G45 | I66 | Transient cerebral ischaemic attacks and related syndromes | Occlusion and stenosis of cerebral arteries, not resulting in cerebral infarction | 34 | 1.14E-20 | 5.51 (3.78, 7.77) |
| G47 | J35 | Sleep disorders | Chronic diseases of tonsils and adenoids | 34 | 7.91E-66 | 24.68 (16.82, 35.1) |
| H66 | J31 | Suppurative and unspecified otitis media | Chronic rhinitis, nasopharyngitis and pharyngitis | 34 | 1.06E-19 | 5.01 (3.47, 6.99) |
| I11 | N18 | Hypertensive heart disease | Chronic kidney disease | 34 | 4.08E-30 | 7.69 (5.32, 10.75) |
| I42 | I47 | Cardiomyopathy | Paroxysmal tachycardia | 34 | 2.36E-55 | 17.42 (11.97, 24.52) |
| I44 | I51 | Atrioventricular and left bundle-branch block | Complications and ill-defined descriptions of heart disease | 34 | 4.15E-36 | 9.93 (6.82, 14) |
| I48 | I51 | Atrial fibrillation and flutter | Complications and ill-defined descriptions of heart disease | 34 | 3.69E-23 | 5.9 (4.08, 8.25) |
| J31 | J37 | Chronic rhinitis, nasopharyngitis and pharyngitis | Chronic laryngitis and laryngotracheitis | 34 | 2.08E-63 | 26.99 (18.11, 39.16) |
| K26 | K63 | Duodenal ulcer | Other diseases of intestine | 34 | 4.22E-12 | 3.4 (2.36, 4.73) |
| K56 | K63 | Paralytic ileus and intestinal obstruction without hernia | Other diseases of intestine | 34 | 4.24E-20 | 5.09 (3.53, 7.09) |
| M89 | N40 | Other disorders of bone | Hyperplasia of prostate | 34 | 2.49E-10 | 3.2 (2.2, 4.5) |
| N71 | N76 | Inflammatory disease of uterus, except cervix | Other inflammation of vagina and vulva | 34 | 6.49E-24 | 6.38 (4.37, 9) |
| B37 | D25 | Candidiasis | Leiomyoma of uterus | 33 | 2.03E-13 | 4.56 (3.01, 6.74) |
| D18 | K31 | Haemangioma and lymphangioma, any site | Other diseases of stomach and duodenum | 33 | 1.15E-08 | 2.76 (1.91, 3.85) |
| D50 | N71 | Iron deficiency anaemia | Inflammatory disease of uterus, except cervix | 33 | 1.52E-18 | 5.19 (3.54, 7.35) |
| E21 | N18 | Hyperparathyroidism and other disorders of parathyroid gland | Chronic kidney disease | 33 | 4.29E-104 | 334.08 (198.03, 569.49) |
| E55 | G63 | Vitamin D deficiency | Polyneuropathy in diseases classified elsewhere | 33 | 6.25E-45 | 17.54 (11.6, 25.85) |
| E78 | G59 | Disorders of lipoprotein metabolism and other lipidaemias | Mononeuropathy in diseases classified elsewhere | 33 | 6.19E-16 | 5.86 (3.78, 8.94) |
| E79 | N18 | Disorders of purine and pyrimidine metabolism | Chronic kidney disease | 33 | 3.42E-21 | 5.53 (3.8, 7.76) |
| I85 | K76 | Oesophageal varices | Other diseases of liver | 33 | 2.46E-12 | 4.16 (2.75, 6.13) |
| I85 | K92 | Oesophageal varices | Other diseases of digestive system | 33 | 4.02E-85 | 57.99 (38.09, 86.2) |
| K20 | K26 | Oesophagitis | Duodenal ulcer | 33 | 2.78E-44 | 13.57 (9.24, 19.26) |
| K22 | K25 | Other diseases of oesophagus | Gastric ulcer | 33 | 5.24E-41 | 11.75 (8.05, 16.58) |
| K22 | K92 | Other diseases of oesophagus | Other diseases of digestive system | 33 | 6.21E-47 | 14.33 (9.79, 20.28) |
| K40 | N50 | Inguinal hernia | Other disorders of male genital organs | 33 | 2.67E-37 | 11.3 (7.66, 16.17) |
| K56 | K65 | Paralytic ileus and intestinal obstruction without hernia | Peritonitis | 33 | 9.25E-24 | 6.12 (4.21, 8.56) |
| M43 | M48 | Other deforming dorsopathies | Other spondylopathies | 33 | 2.38E-61 | 26.39 (17.63, 38.41) |
| N28 | N83 | Other disorders of kidney and ureter, not elsewhere classified | Noninflammatory disorders of ovary, fallopian tube and broad ligament | 33 | 4.85E-08 | 2.72 (1.87, 3.81) |
| N76 | N88 | Other inflammation of vagina and vulva | Other noninflammatory disorders of cervix uteri | 33 | 3.97E-11 | 3.33 (2.29, 4.68) |
| A16 | E77 | Respiratory tuberculosis, not confirmed bacteriologically or histologically | Disorders of glycoprotein metabolism | 32 | 9.62E-19 | 5.04 (3.45, 7.08) |
| A49 | J18 | Bacterial infection of unspecified site | Pneumonia, organism unspecified | 32 | 6.80E-07 | 2.47 (1.69, 3.47) |
| B18 | E77 | Chronic viral hepatitis | Disorders of glycoprotein metabolism | 32 | 1.51E-11 | 3.4 (2.33, 4.78) |
| E06 | G94 | Thyroiditis | Other disorders of brain in diseases classified elsewhere | 32 | 8.17E-70 | 1319.06 (623.31, 3134.88) |
| E70 | I63 | Disorders of aromatic amino-acid metabolism | Cerebral infarction | 32 | 1.47E-17 | 7.39 (4.64, 11.62) |
| E77 | N28 | Disorders of glycoprotein metabolism | Other disorders of kidney and ureter, not elsewhere classified | 32 | 1.60E-06 | 2.4 (1.64, 3.36) |
| F41 | I49 | Other anxiety disorders | Other cardiac arrhythmias | 32 | 9.97E-07 | 2.44 (1.67, 3.44) |
| G43 | M50 | Migraine | Cervical disc disorders | 32 | 6.83E-19 | 5.24 (3.56, 7.43) |
| G44 | J32 | Other headache syndromes | Chronic sinusitis | 32 | 3.22E-07 | 2.51 (1.73, 3.52) |
| G57 | M51 | Mononeuropathies of lower limb | Other intervertebral disc disorders | 32 | 2.92E-25 | 9.75 (6.27, 14.88) |
| H26 | H44 | Other cataract | Disorders of globe | 32 | 1.82E-92 | 80.93 (52.4, 122.22) |
| I11 | N08 | Hypertensive heart disease | Glomerular disorders in diseases classified elsewhere | 32 | 1.36E-07 | 2.6 (1.78, 3.65) |
| I40 | I49 | Acute myocarditis | Other cardiac arrhythmias | 32 | 1.75E-29 | 8.87 (5.97, 12.78) |
| I47 | I51 | Paroxysmal tachycardia | Complications and ill-defined descriptions of heart disease | 32 | 4.05E-15 | 4.18 (2.86, 5.87) |
| I60 | J98 | Subarachnoid haemorrhage | Other respiratory disorders | 32 | 2.20E-13 | 3.84 (2.63, 5.41) |
| I61 | K27 | Intracerebral haemorrhage | Peptic ulcer, site unspecified | 32 | 2.41E-36 | 10.66 (7.24, 15.16) |
| I63 | J69 | Cerebral infarction | Pneumonitis due to solids and liquids | 32 | 1.60E-06 | 2.59 (1.72, 3.76) |
| I66 | I67 | Occlusion and stenosis of cerebral arteries, not resulting in cerebral infarction | Other cerebrovascular diseases | 32 | 4.99E-19 | 5.34 (3.63, 7.6) |
| J45 | J96 | Asthma | Respiratory failure, not elsewhere classified | 32 | 1.51E-48 | 15.47 (10.52, 21.96) |
| J94 | K85 | Other pleural conditions | Acute pancreatitis | 32 | 1.46E-08 | 2.79 (1.92, 3.91) |
| K29 | K44 | Gastritis and duodenitis | Diaphragmatic hernia | 32 | 2.58E-25 | 14.1 (8.55, 23.31) |
| K72 | K80 | Hepatic failure, not elsewhere classified | Cholelithiasis | 32 | 6.93E-07 | 2.55 (1.73, 3.63) |
| M24 | M51 | Other specific joint derangements | Other intervertebral disc disorders | 32 | 2.28E-09 | 3.15 (2.12, 4.52) |
| N20 | N30 | Calculus of kidney and ureter | Cystitis | 32 | 2.21E-14 | 4.15 (2.82, 5.88) |
| N40 | N42 | Hyperplasia of prostate | Other disorders of prostate | 32 | 1.19E-35 | 13.92 (9.08, 20.82) |
| A49 | J20 | Bacterial infection of unspecified site | Acute bronchitis | 31 | 2.29E-07 | 2.6 (1.77, 3.67) |
| B18 | D18 | Chronic viral hepatitis | Haemangioma and lymphangioma, any site | 31 | 1.66E-07 | 2.61 (1.78, 3.67) |
| C54 | N72 | Malignant neoplasm of corpus uteri | Inflammatory disease of cervix uteri | 31 | 7.32E-21 | 7.06 (4.62, 10.49) |
| D25 | N95 | Leiomyoma of uterus | Menopausal and other perimenopausal disorders | 31 | 3.27E-07 | 2.73 (1.83, 3.95) |
| D64 | N87 | Other anaemias | Dysplasia of cervix uteri | 31 | 7.58E-08 | 2.84 (1.91, 4.07) |
| D70 | J98 | Agranulocytosis | Other respiratory disorders | 31 | 9.54E-11 | 3.31 (2.25, 4.67) |
| D86 | J18 | Sarcoidosis | Pneumonia, organism unspecified | 31 | 4.04E-22 | 6.47 (4.35, 9.29) |
| E03 | I79 | Other hypothyroidism | Disorders of arteries, arterioles and capillaries in diseases classified elsewhere | 31 | 1.66E-11 | 3.5 (2.38, 4.95) |
| E05 | I48 | Thyrotoxicosis [hyperthyroidism] | Atrial fibrillation and flutter | 31 | 4.36E-48 | 15.91 (10.75, 22.68) |
| E77 | J96 | Disorders of glycoprotein metabolism | Respiratory failure, not elsewhere classified | 31 | 1.28E-41 | 13.03 (8.81, 18.58) |
| E78 | G90 | Disorders of lipoprotein metabolism and other lipidaemias | Disorders of autonomic nervous system | 31 | 3.82E-07 | 2.77 (1.84, 4.05) |
| E87 | K83 | Other disorders of fluid, electrolyte and acid-base balance | Other diseases of biliary tract | 31 | 1.77E-10 | 3.28 (2.23, 4.64) |
| E87 | N17 | Other disorders of fluid, electrolyte and acid-base balance | Acute renal failure | 31 | 5.34E-39 | 14.86 (9.76, 21.99) |
| G63 | H35 | Polyneuropathy in diseases classified elsewhere | Other retinal disorders | 31 | 9.55E-07 | 2.49 (1.69, 3.51) |
| J94 | J96 | Other pleural conditions | Respiratory failure, not elsewhere classified | 31 | 2.40E-27 | 7.71 (5.22, 10.97) |
| M19 | M23 | Other arthrosis | Internal derangement of knee | 31 | 2.08E-22 | 6.26 (4.24, 8.89) |
| M51 | M60 | Other intervertebral disc disorders | Myositis | 31 | 2.27E-21 | 7.75 (5.01, 11.71) |
| M89 | N28 | Other disorders of bone | Other disorders of kidney and ureter, not elsewhere classified | 31 | 5.09E-10 | 3.17 (2.16, 4.48) |
| N39 | N41 | Other disorders of urinary system | Inflammatory diseases of prostate | 31 | 2.32E-25 | 7.17 (4.86, 10.19) |
| N40 | N45 | Hyperplasia of prostate | Orchitis and epididymitis | 31 | 1.16E-10 | 3.4 (2.3, 4.86) |
| N60 | N64 | Benign mammary dysplasia | Other disorders of breast | 31 | 1.13E-58 | 27.12 (17.88, 39.9) |
| A16 | J44 | Respiratory tuberculosis, not confirmed bacteriologically or histologically | Other chronic obstructive pulmonary disease | 30 | 4.14E-07 | 2.59 (1.75, 3.67) |
| C34 | C77 | Malignant neoplasm of bronchus and lung | Secondary and unspecified malignant neoplasm of lymph nodes | 30 | 7.28E-97 | 94.58 (60.81, 143.14) |
| D27 | N80 | Benign neoplasm of ovary | Endometriosis | 30 | 1.43E-19 | 5.88 (3.93, 8.46) |
| D50 | N88 | Iron deficiency anaemia | Other noninflammatory disorders of cervix uteri | 30 | 1.34E-06 | 2.54 (1.71, 3.61) |
| E04 | J31 | Other nontoxic goitre | Chronic rhinitis, nasopharyngitis and pharyngitis | 30 | 1.09E-06 | 2.49 (1.69, 3.53) |
| E87 | I48 | Other disorders of fluid, electrolyte and acid-base balance | Atrial fibrillation and flutter | 30 | 1.57E-06 | 2.47 (1.67, 3.51) |
| G47 | J96 | Sleep disorders | Respiratory failure, not elsewhere classified | 30 | 1.03E-37 | 11.88 (7.98, 17.01) |
| H20 | H40 | Iridocyclitis | Glaucoma | 30 | 7.11E-97 | 68.55 (45.28, 100.32) |
| H40 | H43 | Glaucoma | Disorders of vitreous body | 30 | 6.64E-73 | 35.19 (23.45, 50.93) |
| H40 | H52 | Glaucoma | Disorders of refraction and accommodation | 30 | 9.95E-57 | 21.82 (14.62, 31.38) |
| I50 | I71 | Heart failure | Aortic aneurysm and dissection | 30 | 8.33E-13 | 4.38 (2.87, 6.47) |
| I83 | I87 | Varicose veins of lower extremities | Other disorders of veins | 30 | 5.46E-72 | 39.77 (26.14, 58.59) |
| J43 | J45 | Emphysema | Asthma | 30 | 8.78E-19 | 5.35 (3.61, 7.62) |
| J98 | N18 | Other respiratory disorders | Chronic kidney disease | 30 | 1.40E-07 | 2.68 (1.81, 3.8) |
| M17 | M71 | Gonarthrosis [arthrosis of knee] | Other bursopathies | 30 | 3.70E-47 | 16.77 (11.21, 24.18) |
| N28 | N41 | Other disorders of kidney and ureter, not elsewhere classified | Inflammatory diseases of prostate | 30 | 3.61E-10 | 3.28 (2.21, 4.67) |
| N41 | N47 | Inflammatory diseases of prostate | Redundant prepuce, phimosis and paraphimosis | 30 | 8.87E-57 | 24.52 (16.22, 35.81) |
| N83 | N87 | Noninflammatory disorders of ovary, fallopian tube and broad ligament | Dysplasia of cervix uteri | 30 | 1.04E-14 | 4.54 (3.03, 6.55) |
| A16 | C34 | Respiratory tuberculosis, not confirmed bacteriologically or histologically | Malignant neoplasm of bronchus and lung | 29 | 1.62E-25 | 7.6 (5.08, 10.9) |
| C15 | K29 | Malignant neoplasm of oesophagus | Gastritis and duodenitis | 29 | 2.16E-07 | 2.95 (1.93, 4.35) |
| D10 | J31 | Benign neoplasm of mouth and pharynx | Chronic rhinitis, nasopharyngitis and pharyngitis | 29 | 1.43E-61 | 40.35 (25.67, 61.83) |
| D17 | K40 | Benign lipomatous neoplasm | Inguinal hernia | 29 | 1.35E-07 | 2.73 (1.84, 3.9) |
| D69 | E77 | Purpura and other haemorrhagic conditions | Disorders of glycoprotein metabolism | 29 | 7.37E-29 | 8.59 (5.76, 12.3) |
| E03 | N28 | Other hypothyroidism | Other disorders of kidney and ureter, not elsewhere classified | 29 | 1.13E-06 | 2.52 (1.7, 3.6) |
| E10 | G63 | Type 1 diabetes mellitus | Polyneuropathy in diseases classified elsewhere | 29 | 3.11E-37 | 14.81 (9.62, 22.1) |
| E53 | E78 | Deficiency of other B group vitamins | Disorders of lipoprotein metabolism and other lipidaemias | 29 | 2.37E-09 | 3.69 (2.37, 5.59) |
| E66 | G63 | Obesity | Polyneuropathy in diseases classified elsewhere | 29 | 2.61E-26 | 8.46 (5.6, 12.31) |
| E72 | F45 | Other disorders of amino-acid metabolism | Somatoform disorders | 29 | 1.24E-10 | 3.48 (2.33, 5) |
| E77 | J44 | Disorders of glycoprotein metabolism | Other chronic obstructive pulmonary disease | 29 | 4.70E-08 | 2.85 (1.91, 4.07) |
| E87 | K72 | Other disorders of fluid, electrolyte and acid-base balance | Hepatic failure, not elsewhere classified | 29 | 9.42E-19 | 5.72 (3.8, 8.27) |
| F45 | I49 | Somatoform disorders | Other cardiac arrhythmias | 29 | 2.88E-07 | 2.68 (1.8, 3.83) |
| G44 | G47 | Other headache syndromes | Sleep disorders | 29 | 1.37E-16 | 4.85 (3.26, 6.92) |
| H11 | H25 | Other disorders of conjunctiva | Senile cataract | 29 | 3.36E-30 | 9.35 (6.25, 13.45) |
| H25 | H36 | Senile cataract | Retinal disorders in diseases classified elsewhere | 29 | 2.52E-18 | 5.41 (3.62, 7.76) |
| H52 | N08 | Disorders of refraction and accommodation | Glomerular disorders in diseases classified elsewhere | 29 | 2.01E-12 | 3.86 (2.59, 5.5) |
| I00 | M19 | Rheumatic fever without mention of heart involvement | Other arthrosis | 29 | 3.26E-39 | 14.41 (9.48, 21.13) |
| I08 | I50 | Multiple valve diseases | Heart failure | 29 | 1.54E-30 | 24.05 (13.96, 41.57) |
| I49 | J96 | Other cardiac arrhythmias | Respiratory failure, not elsewhere classified | 29 | 3.42E-07 | 2.67 (1.79, 3.82) |
| J02 | J20 | Acute pharyngitis | Acute bronchitis | 29 | 2.46E-18 | 5.57 (3.71, 8.04) |
| J03 | J18 | Acute tonsillitis | Pneumonia, organism unspecified | 29 | 6.85E-07 | 2.59 (1.74, 3.7) |
| J32 | J38 | Chronic sinusitis | Diseases of vocal cords and larynx, not elsewhere classified | 29 | 3.69E-09 | 3.08 (2.07, 4.38) |
| N02 | N18 | Recurrent and persistent haematuria | Chronic kidney disease | 29 | 1.47E-80 | 54.46 (35.43, 81.01) |
| N34 | N41 | Urethritis and urethral syndrome | Inflammatory diseases of prostate | 29 | 2.33E-66 | 38.59 (25.1, 57.41) |
| A49 | K25 | Bacterial infection of unspecified site | Gastric ulcer | 28 | 6.84E-23 | 6.87 (4.57, 9.88) |
| E02 | E04 | Subclinical iodine-deficiency hypothyroidism | Other nontoxic goitre | 28 | 6.84E-28 | 8.88 (5.88, 12.88) |
| E02 | E06 | Subclinical iodine-deficiency hypothyroidism | Thyroiditis | 28 | 4.66E-49 | 19.5 (12.85, 28.41) |
| E26 | I10 | Hyperaldosteronism | Essential (primary) hypertension | 28 | 7.32E-09 | 4.95 (2.87, 8.54) |
| G31 | I67 | Other degenerative diseases of nervous system, not elsewhere classified | Other cerebrovascular diseases | 28 | 2.57E-13 | 4.37 (2.89, 6.34) |
| G40 | J98 | Epilepsy | Other respiratory disorders | 28 | 1.76E-07 | 2.75 (1.84, 3.95) |
| G47 | J44 | Sleep disorders | Other chronic obstructive pulmonary disease | 28 | 1.21E-07 | 2.81 (1.87, 4.03) |
| G72 | J06 | Other myopathies | Acute upper respiratory infections of multiple and unspecified sites | 28 | 9.71E-26 | 8.7 (5.69, 12.79) |
| H36 | H52 | Retinal disorders in diseases classified elsewhere | Disorders of refraction and accommodation | 28 | 6.25E-18 | 5.42 (3.61, 7.79) |
| H66 | H91 | Suppurative and unspecified otitis media | Other hearing loss | 28 | 1.18E-33 | 10.77 (7.16, 15.53) |
| I63 | I77 | Cerebral infarction | Other disorders of arteries and arterioles | 28 | 8.36E-07 | 2.93 (1.88, 4.42) |
| J32 | J45 | Chronic sinusitis | Asthma | 28 | 1.58E-07 | 2.75 (1.84, 3.94) |
| J44 | J84 | Other chronic obstructive pulmonary disease | Other interstitial pulmonary diseases | 28 | 1.43E-15 | 4.86 (3.22, 7.02) |
| K25 | K63 | Gastric ulcer | Other diseases of intestine | 28 | 1.27E-08 | 3 (2.01, 4.3) |
| K56 | K85 | Paralytic ileus and intestinal obstruction without hernia | Acute pancreatitis | 28 | 6.33E-13 | 4.05 (2.7, 5.8) |
| K62 | L29 | Other diseases of anus and rectum | Pruritus | 28 | 9.72E-58 | 41.73 (26.08, 65.26) |
| M13 | M65 | Other arthritis | Synovitis and tenosynovitis | 28 | 3.80E-38 | 12.96 (8.59, 18.74) |
| N02 | N03 | Recurrent and persistent haematuria | Chronic nephritic syndrome | 28 | 5.94E-83 | 60.64 (39.22, 90.58) |
| N13 | N30 | Obstructive and reflux uropathyC | Cystitis | 28 | 9.51E-25 | 7.65 (5.08, 11.07) |
| N72 | N90 | Inflammatory disease of cervix uteri | Other noninflammatory disorders of vulva and perineum | 28 | 2.84E-19 | 7.71 (4.87, 11.9) |
| N86 | N88 | Erosion and ectropion of cervix uteri | Other noninflammatory disorders of cervix uteri | 28 | 4.40E-47 | 21.41 (13.87, 31.91) |
| A09 | K31 | Other gastroenteritis and colitis of infectious and unspecified origin | Other diseases of stomach and duodenum | 27 | 5.25E-43 | 18.15 (11.76, 26.96) |
| A59 | N73 | Trichomoniasis | Other female pelvic inflammatory diseases | 27 | 1.29E-21 | 9.15 (5.72, 14.24) |
| A59 | N76 | Trichomoniasis | Other inflammation of vagina and vulva | 27 | 1.62E-42 | 22.93 (14.4, 35.47) |
| B18 | D69 | Chronic viral hepatitis | Purpura and other haemorrhagic conditions | 27 | 5.34E-12 | 3.91 (2.59, 5.65) |
| B19 | K74 | Unspecified viral hepatitis | Fibrosis and cirrhosis of liver | 27 | 4.61E-44 | 18.81 (12.19, 27.93) |
| D25 | D28 | Leiomyoma of uterus | Benign neoplasm of other and unspecified female genital organs | 27 | 5.78E-15 | 6.48 (4, 10.24) |
| D26 | N80 | Other benign neoplasms of uterus | Endometriosis | 27 | 1.21E-16 | 5.45 (3.57, 7.97) |
| D50 | D70 | Iron deficiency anaemia | Agranulocytosis | 27 | 3.71E-08 | 2.99 (1.98, 4.33) |
| D70 | E05 | Agranulocytosis | Thyrotoxicosis [hyperthyroidism] | 27 | 3.58E-26 | 8.32 (5.49, 12.08) |
| D70 | E77 | Agranulocytosis | Disorders of glycoprotein metabolism | 27 | 6.84E-27 | 8.54 (5.64, 12.39) |
| E02 | E72 | Subclinical iodine-deficiency hypothyroidism | Other disorders of amino-acid metabolism | 27 | 1.75E-10 | 3.63 (2.39, 5.28) |
| E04 | N60 | Other nontoxic goitre | Benign mammary dysplasia | 27 | 1.19E-16 | 5.28 (3.48, 7.67) |
| E66 | I50 | Obesity | Heart failure | 27 | 7.07E-07 | 2.8 (1.83, 4.11) |
| E87 | I12 | Other disorders of fluid, electrolyte and acid-base balance | Hypertensive renal disease | 27 | 1.19E-14 | 4.78 (3.14, 6.98) |
| E87 | J03 | Other disorders of fluid, electrolyte and acid-base balance | Acute tonsillitis | 27 | 1.24E-06 | 2.61 (1.73, 3.77) |
| F01 | I10 | Vascular dementia | Essential (primary) hypertension | 27 | 2.55E-09 | 8.74 (4.4, 18.58) |
| F41 | I65 | Other anxiety disorders | Occlusion and stenosis of precerebral arteries, not resulting in cerebral infarction | 27 | 2.89E-09 | 3.29 (2.17, 4.77) |
| G54 | G55 | Nerve root and plexus disorders | Nerve root and plexus compressions in diseases classified elsewhere | 27 | 1.26E-06 | 2.59 (1.72, 3.72) |
| H90 | J32 | Conductive and sensorineural hearing loss | Chronic sinusitis | 27 | 3.59E-11 | 3.7 (2.45, 5.35) |
| I86 | N41 | Varicose veins of other sites | Inflammatory diseases of prostate | 27 | 2.44E-62 | 38.79 (24.77, 58.68) |
| J30 | J33 | Vasomotor and allergic rhinitis | Nasal polyp | 27 | 3.17E-81 | 55.35 (35.88, 82.09) |
| M19 | M25 | Other arthrosis | Other joint disorders, not elsewhere classified | 27 | 1.62E-13 | 4.34 (2.87, 6.28) |
| M48 | M81 | Other spondylopathies | Osteoporosis without pathological fracture | 27 | 2.54E-10 | 3.51 (2.32, 5.07) |
| M81 | N08 | Osteoporosis without pathological fracture | Glomerular disorders in diseases classified elsewhere | 27 | 6.12E-12 | 3.96 (2.61, 5.72) |
| N71 | N83 | Inflammatory disease of uterus, except cervix | Noninflammatory disorders of ovary, fallopian tube and broad ligament | 27 | 1.43E-08 | 3.16 (2.07, 4.61) |
| N76 | N81 | Other inflammation of vagina and vulva | Female genital prolapse | 27 | 4.20E-18 | 5.86 (3.84, 8.56) |
| C22 | E77 | Malignant neoplasm of liver and intrahepatic bile ducts | Disorders of glycoprotein metabolism | 26 | 8.28E-40 | 15.61 (10.14, 23) |
| E53 | E72 | Deficiency of other B group vitamins | Other disorders of amino-acid metabolism | 26 | 6.46E-29 | 12.86 (8.06, 19.85) |
| E66 | I70 | Obesity | Atherosclerosis | 26 | 1.18E-12 | 4.53 (2.93, 6.71) |
| G62 | M47 | Other polyneuropathies | Spondylosis | 26 | 1.31E-08 | 3.38 (2.17, 5.06) |
| G93 | J96 | Other disorders of brain | Respiratory failure, not elsewhere classified | 26 | 3.77E-39 | 14.93 (9.73, 21.92) |
| H20 | H26 | Iridocyclitis | Other cataract | 26 | 6.85E-48 | 21.31 (13.79, 31.55) |
| I34 | I50 | Nonrheumatic mitral valve disorders | Heart failure | 26 | 3.62E-23 | 14.8 (8.64, 25.1) |
| I48 | J98 | Atrial fibrillation and flutter | Other respiratory disorders | 26 | 3.57E-07 | 2.79 (1.83, 4.06) |
| I98 | K92 | Other disorders of circulatory system in diseases classified elsewhere | Other diseases of digestive system | 26 | 2.49E-61 | 41.09 (25.94, 62.86) |
| J34 | J38 | Other disorders of nose and nasal sinuses | Diseases of vocal cords and larynx, not elsewhere classified | 26 | 4.60E-10 | 3.51 (2.31, 5.1) |
| J44 | J64 | Other chronic obstructive pulmonary disease | Unspecified pneumoconiosis | 26 | 3.60E-48 | 27.85 (17.5, 42.84) |
| K29 | M07 | Gastritis and duodenitis | Psoriatic and enteropathic arthropathies | 26 | 1.30E-10 | 4.27 (2.69, 6.55) |
| K58 | K63 | Irritable bowel syndrome | Other diseases of intestine | 26 | 7.98E-53 | 29.19 (18.56, 44.22) |
| N21 | N40 | Calculus of lower urinary tract | Hyperplasia of prostate | 26 | 1.16E-20 | 7.81 (4.98, 11.79) |
| N81 | N83 | Female genital prolapse | Noninflammatory disorders of ovary, fallopian tube and broad ligament | 26 | 1.56E-10 | 3.79 (2.46, 5.57) |
| D64 | E21 | Other anaemias | Hyperparathyroidism and other disorders of parathyroid gland | 25 | 1.93E-40 | 42.47 (24.4, 72.97) |
| D64 | I80 | Other anaemias | Phlebitis and thrombophlebitis | 25 | 1.48E-08 | 3.29 (2.12, 4.86) |
| E03 | G47 | Other hypothyroidism | Sleep disorders | 25 | 4.80E-17 | 5.68 (3.7, 8.33) |
| E72 | G43 | Other disorders of amino-acid metabolism | Migraine | 25 | 1.91E-12 | 4.41 (2.84, 6.52) |
| E77 | J43 | Disorders of glycoprotein metabolism | Emphysema | 25 | 7.92E-10 | 3.57 (2.32, 5.23) |
| E79 | M10 | Disorders of purine and pyrimidine metabolism | Gout | 25 | 3.50E-11 | 3.92 (2.55, 5.75) |
| E87 | K27 | Other disorders of fluid, electrolyte and acid-base balance | Peptic ulcer, site unspecified | 25 | 3.59E-09 | 3.41 (2.21, 5.01) |
| F41 | G44 | Other anxiety disorders | Other headache syndromes | 25 | 1.33E-19 | 6.51 (4.23, 9.55) |
| G44 | G54 | Other headache syndromes | Nerve root and plexus disorders | 25 | 2.33E-07 | 2.87 (1.87, 4.19) |
| G54 | M75 | Nerve root and plexus disorders | Shoulder lesions | 25 | 6.14E-21 | 7.02 (4.55, 10.31) |
| G62 | M50 | Other polyneuropathies | Cervical disc disorders | 25 | 1.92E-25 | 9.72 (6.2, 14.63) |
| G90 | M47 | Disorders of autonomic nervous system | Spondylosis | 25 | 1.74E-07 | 3.13 (1.99, 4.71) |
| H11 | H35 | Other disorders of conjunctiva | Other retinal disorders | 25 | 1.73E-22 | 7.52 (4.89, 11.03) |
| H26 | H47 | Other cataract | Other disorders of optic [2nd] nerve and visual pathways | 25 | 4.27E-46 | 21.63 (13.84, 32.35) |
| H35 | N08 | Other retinal disorders | Glomerular disorders in diseases classified elsewhere | 25 | 1.68E-08 | 3.19 (2.08, 4.67) |
| H36 | I11 | Retinal disorders in diseases classified elsewhere | Hypertensive heart disease | 25 | 3.18E-07 | 2.86 (1.86, 4.18) |
| H36 | M81 | Retinal disorders in diseases classified elsewhere | Osteoporosis without pathological fracture | 25 | 4.64E-14 | 4.81 (3.12, 7.06) |
| I07 | I50 | Rheumatic tricuspid valve diseases | Heart failure | 25 | 1.38E-27 | 24.3 (13.64, 43.31) |
| I26 | I80 | Pulmonary embolism | Phlebitis and thrombophlebitis | 25 | 1.51E-102 | 257.94 (153.59, 424.65) |
| J47 | J96 | Bronchiectasis | Respiratory failure, not elsewhere classified | 25 | 1.02E-37 | 14.9 (9.62, 22.03) |
| K63 | K92 | Other diseases of intestine | Other diseases of digestive system | 25 | 1.40E-08 | 3.2 (2.08, 4.67) |
| K70 | K74 | Alcoholic liver disease | Fibrosis and cirrhosis of liver | 25 | 7.39E-36 | 15.12 (9.65, 22.69) |
| K76 | K86 | Other diseases of liver | Other diseases of pancreas | 25 | 2.05E-09 | 4.04 (2.51, 6.29) |
| M17 | M81 | Gonarthrosis [arthrosis of knee] | Osteoporosis without pathological fracture | 25 | 1.78E-16 | 5.57 (3.61, 8.2) |
| M54 | N91 | Dorsalgia | Absent, scanty and rare menstruation | 25 | 1.70E-31 | 11.95 (7.69, 17.76) |
| N10 | N20 | Acute tubulo-interstitial nephritis | Calculus of kidney and ureter | 25 | 7.36E-07 | 2.79 (1.81, 4.09) |
| N10 | N40 | Acute tubulo-interstitial nephritis | Hyperplasia of prostate | 25 | 9.32E-17 | 6.22 (3.96, 9.36) |
| N84 | N87 | Polyp of female genital tract | Dysplasia of cervix uteri | 25 | 3.06E-16 | 5.65 (3.64, 8.39) |
| A16 | J64 | Respiratory tuberculosis, not confirmed bacteriologically or histologically | Unspecified pneumoconiosis | 24 | 3.84E-45 | 25.7 (16.02, 39.63) |
| A18 | M01 | Tuberculosis of other organs | Direct infections of joint in infectious and parasitic diseases classified elsewhere | 24 | 5.08E-43 | 4884.12 (1685.65, 20701.95) |
| B49 | N39 | Unspecified mycosis | Other disorders of urinary system | 24 | 4.48E-27 | 10.43 (6.64, 15.65) |
| C22 | C78 | Malignant neoplasm of liver and intrahepatic bile ducts | Secondary malignant neoplasm of respiratory and digestive organs | 24 | 2.38E-78 | 70.13 (43.94, 107.27) |
| D25 | K66 | Leiomyoma of uterus | Other disorders of peritoneum | 24 | 4.24E-07 | 3.2 (2, 4.91) |
| E05 | G73 | Thyrotoxicosis [hyperthyroidism] | Disorders of myoneural junction and muscle in diseases classified elsewhere | 24 | 1.27E-78 | 204.52 (116.56, 356.25) |
| E10 | I79 | Type 1 diabetes mellitus | Disorders of arteries, arterioles and capillaries in diseases classified elsewhere | 24 | 7.16E-37 | 18.09 (11.31, 27.78) |
| E72 | G31 | Other disorders of amino-acid metabolism | Other degenerative diseases of nervous system, not elsewhere classified | 24 | 2.90E-07 | 3.03 (1.94, 4.52) |
| E83 | N18 | Disorders of mineral metabolism | Chronic kidney disease | 24 | 2.77E-46 | 22.19 (14.14, 33.23) |
| E87 | K70 | Other disorders of fluid, electrolyte and acid-base balance | Alcoholic liver disease | 24 | 4.43E-18 | 6.7 (4.25, 10.09) |
| F02 | G20 | Dementia in other diseases classified elsewhere | Parkinson disease | 24 | 8.05E-28 | 86984.43 (17389.88, 1585169.73) |
| F32 | F41 | Depressive episode | Other anxiety disorders | 24 | 3.70E-45 | 21.18 (13.5, 31.67) |
| H11 | H26 | Other disorders of conjunctiva | Other cataract | 24 | 4.24E-16 | 5.54 (3.57, 8.17) |
| I09 | I49 | Other rheumatic heart diseases | Other cardiac arrhythmias | 24 | 2.43E-20 | 7.93 (4.99, 12.07) |
| I12 | N28 | Hypertensive renal disease | Other disorders of kidney and ureter, not elsewhere classified | 24 | 1.29E-16 | 5.93 (3.79, 8.85) |
| I24 | I49 | Other acute ischaemic heart diseases | Other cardiac arrhythmias | 24 | 4.53E-08 | 3.21 (2.06, 4.77) |
| I43 | I79 | Cardiomyopathy in diseases classified elsewhere | Disorders of arteries, arterioles and capillaries in diseases classified elsewhere | 24 | 9.33E-45 | 27.63 (17.05, 43.13) |
| I65 | I77 | Occlusion and stenosis of precerebral arteries, not resulting in cerebral infarction | Other disorders of arteries and arterioles | 24 | 5.28E-26 | 11.38 (7.09, 17.55) |
| K76 | N05 | Other diseases of liver | Unspecified nephritic syndrome | 24 | 2.26E-07 | 3.32 (2.06, 5.14) |
| M13 | M48 | Other arthritis | Other spondylopathies | 24 | 4.13E-13 | 4.6 (2.96, 6.79) |
| M23 | M71 | Internal derangement of knee | Other bursopathies | 24 | 4.49E-54 | 28.69 (18.29, 42.93) |
| N02 | N04 | Recurrent and persistent haematuria | Nephrotic syndrome | 24 | 2.72E-92 | 109.58 (68.23, 169.02) |
| N03 | N40 | Chronic nephritic syndrome | Hyperplasia of prostate | 24 | 7.30E-07 | 2.93 (1.87, 4.35) |
| N13 | N19 | Obstructive and reflux uropathyC | Unspecified kidney failure | 24 | 4.18E-26 | 10.09 (6.42, 15.15) |
| N20 | N34 | Calculus of kidney and ureter | Urethritis and urethral syndrome | 24 | 5.38E-10 | 3.82 (2.44, 5.7) |
| A15 | J98 | Respiratory tuberculosis, bacteriologically and histologically confirmed | Other respiratory disorders | 23 | 6.07E-23 | 9.12 (5.73, 13.85) |
| A49 | K63 | Bacterial infection of unspecified site | Other diseases of intestine | 23 | 5.97E-16 | 5.69 (3.63, 8.47) |
| A59 | D25 | Trichomoniasis | Leiomyoma of uterus | 23 | 7.92E-09 | 4.04 (2.46, 6.36) |
| D12 | K62 | Benign neoplasm of colon, rectum, anus and anal canal | Other diseases of anus and rectum | 23 | 3.46E-32 | 14.58 (9.11, 22.27) |
| D25 | D62 | Leiomyoma of uterus | Acute posthaemorrhagic anaemia | 23 | 8.39E-13 | 6.35 (3.79, 10.33) |
| D26 | D50 | Other benign neoplasms of uterus | Iron deficiency anaemia | 23 | 1.97E-07 | 3.14 (2, 4.7) |
| D62 | K26 | Acute posthaemorrhagic anaemia | Duodenal ulcer | 23 | 8.42E-44 | 25.19 (15.61, 38.92) |
| E03 | H36 | Other hypothyroidism | Retinal disorders in diseases classified elsewhere | 23 | 1.34E-09 | 3.67 (2.34, 5.45) |
| E05 | G72 | Thyrotoxicosis [hyperthyroidism] | Other myopathies | 23 | 1.77E-56 | 37.99 (23.67, 58.26) |
| E10 | N08 | Type 1 diabetes mellitus | Glomerular disorders in diseases classified elsewhere | 23 | 1.40E-35 | 17.64 (10.96, 27.17) |
| E27 | K76 | Other disorders of adrenal gland | Other diseases of liver | 23 | 2.49E-10 | 4.77 (2.88, 7.62) |
| E53 | I70 | Deficiency of other B group vitamins | Atherosclerosis | 23 | 2.72E-18 | 8.27 (5.04, 13.07) |
| E55 | H36 | Vitamin D deficiency | Retinal disorders in diseases classified elsewhere | 23 | 5.72E-44 | 25.35 (15.7, 39.22) |
| E77 | K65 | Disorders of glycoprotein metabolism | Peritonitis | 23 | 5.28E-12 | 4.37 (2.79, 6.48) |
| E78 | I73 | Disorders of lipoprotein metabolism and other lipidaemias | Other peripheral vascular diseases | 23 | 3.69E-10 | 4.98 (2.96, 8.13) |
| E88 | H36 | Other metabolic disorders | Retinal disorders in diseases classified elsewhere | 23 | 3.66E-54 | 45.88 (27.72, 73.27) |
| G40 | I61 | Epilepsy | Intracerebral haemorrhage | 23 | 5.32E-11 | 4.08 (2.6, 6.06) |
| G59 | K76 | Mononeuropathy in diseases classified elsewhere | Other diseases of liver | 23 | 8.80E-09 | 4.04 (2.46, 6.39) |
| G62 | I70 | Other polyneuropathies | Atherosclerosis | 23 | 1.02E-13 | 5.57 (3.46, 8.57) |
| H11 | H16 | Other disorders of conjunctiva | Keratitis | 23 | 2.37E-38 | 17.15 (10.86, 25.77) |
| H11 | H43 | Other disorders of conjunctiva | Disorders of vitreous body | 23 | 3.40E-28 | 10.96 (6.96, 16.37) |
| H28 | H36 | Cataract and other disorders of lens in diseases classified elsewhere | Retinal disorders in diseases classified elsewhere | 23 | 1.19E-58 | 86.17 (49.67, 146.84) |
| H34 | H52 | Retinal vascular occlusions | Disorders of refraction and accommodation | 23 | 2.06E-37 | 16.44 (10.42, 24.68) |
| I11 | I48 | Hypertensive heart disease | Atrial fibrillation and flutter | 23 | 1.72E-15 | 5.58 (3.55, 8.32) |
| I21 | I44 | Acute myocardial infarction | Atrioventricular and left bundle-branch block | 23 | 4.14E-21 | 7.96 (5.04, 11.97) |
| I27 | J47 | Other pulmonary heart diseases | Bronchiectasis | 23 | 1.60E-42 | 20.65 (13.03, 31.15) |
| I31 | J98 | Other diseases of pericardium | Other respiratory disorders | 23 | 1.96E-23 | 9.34 (5.87, 14.18) |
| J42 | J47 | Unspecified chronic bronchitis | Bronchiectasis | 23 | 5.36E-14 | 5.03 (3.21, 7.48) |
| J47 | J84 | Bronchiectasis | Other interstitial pulmonary diseases | 23 | 1.45E-30 | 12.31 (7.81, 18.45) |
| J84 | J96 | Other interstitial pulmonary diseases | Respiratory failure, not elsewhere classified | 23 | 1.07E-38 | 17.69 (11.17, 26.64) |
| K20 | K25 | Oesophagitis | Gastric ulcer | 23 | 1.87E-25 | 9.73 (6.17, 14.58) |
| K57 | K76 | Diverticular disease of intestine | Other diseases of liver | 23 | 1.79E-06 | 3.07 (1.89, 4.78) |
| K72 | K92 | Hepatic failure, not elsewhere classified | Other diseases of digestive system | 23 | 6.14E-34 | 14.72 (9.28, 22.21) |
| M13 | M23 | Other arthritis | Internal derangement of knee | 23 | 3.49E-41 | 19.04 (12.06, 28.58) |
| N15 | N20 | Other renal tubulo-interstitial diseases | Calculus of kidney and ureter | 23 | 9.53E-29 | 16.31 (9.78, 26.3) |
| N70 | N81 | Salpingitis and oophoritis | Female genital prolapse | 23 | 1.07E-17 | 6.58 (4.16, 9.88) |
| A18 | N29 | Tuberculosis of other organs | Other disorders of kidney and ureter in diseases classified elsewhere | 22 | 4.77E-41 | 4116.4 (1409.95, 17503.72) |
| A49 | N76 | Bacterial infection of unspecified site | Other inflammation of vagina and vulva | 22 | 1.97E-14 | 5.57 (3.49, 8.44) |
| A98 | E87 | Other viral haemorrhagic fevers, not elsewhere classified | Other disorders of fluid, electrolyte and acid-base balance | 22 | 2.10E-16 | 6.55 (4.07, 10.02) |
| B18 | I85 | Chronic viral hepatitis | Oesophageal varices | 22 | 2.11E-36 | 19.7 (12.08, 30.68) |
| B90 | J43 | Sequelae of tuberculosis | Emphysema | 22 | 3.12E-29 | 13 (8.09, 19.86) |
| C16 | D64 | Malignant neoplasm of stomach | Other anaemias | 22 | 1.80E-10 | 4.2 (2.62, 6.36) |
| C18 | D64 | Malignant neoplasm of colon | Other anaemias | 22 | 1.69E-23 | 10.34 (6.41, 15.96) |
| C18 | D64 | Malignant neoplasm of colon | Other anaemias | 22 | 1.69E-23 | 10.34 (6.41, 15.96) |
| C18 | K56 | Malignant neoplasm of colon | Paralytic ileus and intestinal obstruction without hernia | 22 | 7.26E-46 | 26.73 (16.54, 41.12) |
| C56 | N72 | Malignant neoplasm of ovary | Inflammatory disease of cervix uteri | 22 | 5.43E-12 | 5.26 (3.21, 8.25) |
| D53 | E87 | Other nutritional anaemias | Other disorders of fluid, electrolyte and acid-base balance | 22 | 4.07E-11 | 4.36 (2.73, 6.59) |
| D86 | J20 | Sarcoidosis | Acute bronchitis | 22 | 6.77E-13 | 5.01 (3.13, 7.59) |
| E02 | N40 | Subclinical iodine-deficiency hypothyroidism | Hyperplasia of prostate | 22 | 6.80E-12 | 4.98 (3.08, 7.67) |
| E04 | M81 | Other nontoxic goitre | Osteoporosis without pathological fracture | 22 | 1.71E-09 | 3.78 (2.39, 5.68) |
| E06 | I79 | Thyroiditis | Disorders of arteries, arterioles and capillaries in diseases classified elsewhere | 22 | 7.39E-13 | 4.87 (3.07, 7.31) |
| E06 | K82 | Thyroiditis | Other diseases of gallbladder | 22 | 9.35E-07 | 2.92 (1.84, 4.36) |
| E13 | K76 | Other specified diabetes mellitus | Other diseases of liver | 22 | 2.41E-07 | 3.52 (2.13, 5.58) |
| E24 | M06 | Cushing syndrome | Other rheumatoid arthritis | 22 | 9.52E-64 | 444.97 (221.9, 927.74) |
| E53 | G32 | Deficiency of other B group vitamins | Other degenerative disorders of nervous system in diseases classified elsewhere | 22 | 1.94E-40 | 22887.63 (6491.73, 145653.32) |
| E66 | I79 | Obesity | Disorders of arteries, arterioles and capillaries in diseases classified elsewhere | 22 | 2.04E-23 | 9.7 (6.05, 14.78) |
| E72 | I12 | Other disorders of amino-acid metabolism | Hypertensive renal disease | 22 | 7.31E-08 | 3.33 (2.08, 5.03) |
| E77 | K85 | Disorders of glycoprotein metabolism | Acute pancreatitis | 22 | 3.91E-08 | 3.31 (2.09, 4.95) |
| E87 | I31 | Other disorders of fluid, electrolyte and acid-base balance | Other diseases of pericardium | 22 | 3.36E-16 | 6.4 (3.98, 9.76) |
| G91 | I61 | Hydrocephalus | Intracerebral haemorrhage | 22 | 7.10E-33 | 15.8 (9.78, 24.3) |
| G93 | N18 | Other disorders of brain | Chronic kidney disease | 22 | 2.65E-20 | 7.63 (4.81, 11.46) |
| H02 | H11 | Other disorders of eyelid | Other disorders of conjunctiva | 22 | 6.11E-44 | 23.63 (14.72, 36.07) |
| H90 | J34 | Conductive and sensorineural hearing loss | Other disorders of nose and nasal sinuses | 22 | 2.40E-10 | 4.01 (2.53, 6) |
| I31 | J18 | Other diseases of pericardium | Pneumonia, organism unspecified | 22 | 5.94E-15 | 5.9 (3.67, 9) |
| I35 | I38 | Nonrheumatic aortic valve disorders | Endocarditis, valve unspecified | 22 | 8.76E-124 | 680.06 (389.9, 1152.73) |
| I86 | K74 | Varicose veins of other sites | Fibrosis and cirrhosis of liver | 22 | 3.20E-33 | 16.46 (10.16, 25.42) |
| J34 | J35 | Other disorders of nose and nasal sinuses | Chronic diseases of tonsils and adenoids | 22 | 1.06E-22 | 9.09 (5.69, 13.79) |
| J93 | J98 | Pneumothorax | Other respiratory disorders | 22 | 2.14E-15 | 5.95 (3.72, 9.03) |
| N73 | N95 | Other female pelvic inflammatory diseases | Menopausal and other perimenopausal disorders | 22 | 1.73E-08 | 3.74 (2.32, 5.71) |
| A18 | I32 | Tuberculosis of other organs | Pericarditis in diseases classified elsewhere | 21 | 1.15E-76 | 1154.24 (558.7, 2518.14) |
| A41 | E87 | Other sepsis | Other disorders of fluid, electrolyte and acid-base balance | 21 | 1.69E-23 | 11.37 (6.88, 17.95) |
| B18 | K72 | Chronic viral hepatitis | Hepatic failure, not elsewhere classified | 21 | 3.50E-19 | 7.76 (4.81, 11.85) |
| B90 | J47 | Sequelae of tuberculosis | Bronchiectasis | 21 | 8.45E-43 | 23.99 (14.79, 36.87) |
| B90 | J98 | Sequelae of tuberculosis | Other respiratory disorders | 21 | 4.37E-14 | 5.63 (3.48, 8.59) |
| C22 | K92 | Malignant neoplasm of liver and intrahepatic bile ducts | Other diseases of digestive system | 21 | 3.36E-24 | 10.18 (6.31, 15.54) |
| C78 | J18 | Secondary malignant neoplasm of respiratory and digestive organs | Pneumonia, organism unspecified | 21 | 2.98E-11 | 4.61 (2.85, 7.07) |
| D18 | E77 | Haemangioma and lymphangioma, any site | Disorders of glycoprotein metabolism | 21 | 2.23E-08 | 3.47 (2.17, 5.23) |
| D26 | N76 | Other benign neoplasms of uterus | Other inflammation of vagina and vulva | 21 | 9.24E-08 | 3.37 (2.09, 5.12) |
| E11 | E71 | Type 2 diabetes mellitus | Disorders of branched-chain amino-acid metabolism and fatty-acid metabolism | 21 | 2.12E-11 | 7.9 (4.28, 14.49) |
| E11 | L28 | Type 2 diabetes mellitus | Lichen simplex chronicus and prurigo | 21 | 9.08E-07 | 3.56 (2.09, 5.81) |
| E53 | I65 | Deficiency of other B group vitamins | Occlusion and stenosis of precerebral arteries, not resulting in cerebral infarction | 21 | 1.64E-25 | 13.53 (8.1, 21.61) |
| E53 | I67 | Deficiency of other B group vitamins | Other cerebrovascular diseases | 21 | 3.65E-24 | 12.04 (7.26, 19.07) |
| E66 | G47 | Obesity | Sleep disorders | 21 | 1.83E-31 | 14.83 (9.15, 22.74) |
| E66 | N08 | Obesity | Glomerular disorders in diseases classified elsewhere | 21 | 2.52E-21 | 8.93 (5.52, 13.68) |
| E87 | G72 | Other disorders of fluid, electrolyte and acid-base balance | Other myopathies | 21 | 5.23E-14 | 5.76 (3.55, 8.86) |
| G55 | M89 | Nerve root and plexus compressions in diseases classified elsewhere | Other disorders of bone | 21 | 8.28E-09 | 3.61 (2.26, 5.45) |
| H10 | H11 | Conjunctivitis | Other disorders of conjunctiva | 21 | 3.93E-38 | 19.68 (12.15, 30.18) |
| H10 | H52 | Conjunctivitis | Disorders of refraction and accommodation | 21 | 2.41E-40 | 21.48 (13.27, 32.93) |
| H11 | H18 | Other disorders of conjunctiva | Other disorders of cornea | 21 | 2.44E-67 | 200.65 (109.47, 365.87) |
| H16 | H52 | Keratitis | Disorders of refraction and accommodation | 21 | 7.99E-34 | 15.96 (9.9, 24.35) |
| H21 | H26 | Other disorders of iris and ciliary body | Other cataract | 21 | 2.06E-57 | 56.22 (33.49, 90.46) |
| H36 | N18 | Retinal disorders in diseases classified elsewhere | Chronic kidney disease | 21 | 5.84E-10 | 4 (2.5, 6.04) |
| I12 | N40 | Hypertensive renal disease | Hyperplasia of prostate | 21 | 5.70E-09 | 3.89 (2.39, 6) |
| I15 | N03 | Secondary hypertension | Chronic nephritic syndrome | 21 | 7.37E-48 | 29.41 (18.11, 45.28) |
| I21 | I47 | Acute myocardial infarction | Paroxysmal tachycardia | 21 | 3.03E-09 | 3.78 (2.36, 5.71) |
| I43 | N18 | Cardiomyopathy in diseases classified elsewhere | Chronic kidney disease | 21 | 7.14E-61 | 56.04 (33.81, 88.64) |
| K21 | K27 | Gastro-oesophageal reflux disease | Peptic ulcer, site unspecified | 21 | 1.61E-16 | 6.49 (4.03, 9.86) |
| K22 | K63 | Other diseases of oesophagus | Other diseases of intestine | 21 | 2.37E-18 | 7.22 (4.49, 10.97) |
| K35 | K66 | Acute appendicitis | Other disorders of peritoneum | 21 | 7.06E-20 | 8.1 (5.01, 12.37) |
| K83 | N28 | Other diseases of biliary tract | Other disorders of kidney and ureter, not elsewhere classified | 21 | 6.40E-08 | 3.37 (2.1, 5.1) |
| N19 | N28 | Unspecified kidney failure | Other disorders of kidney and ureter, not elsewhere classified | 21 | 8.04E-16 | 6.45 (3.98, 9.89) |
| N34 | N40 | Urethritis and urethral syndrome | Hyperplasia of prostate | 21 | 1.52E-13 | 6.15 (3.75, 9.59) |
| N40 | N50 | Hyperplasia of prostate | Other disorders of male genital organs | 21 | 1.25E-09 | 4.12 (2.53, 6.35) |
| A18 | K93 | Tuberculosis of other organs | Disorders of other digestive organs in diseases classified elsewhere | 20 | 7.13E-20 | 11932.87 (2459.98, 214966.71) |
| A59 | N72 | Trichomoniasis | Inflammatory disease of cervix uteri | 20 | 3.51E-10 | 4.95 (2.93, 7.98) |
| B02 | H62 | Zoster [herpes zoster] | Disorders of external ear in diseases classified elsewhere | 20 | 9.91E-49 | 492.38 (223.08, 1196.56) |
| B16 | K92 | Acute hepatitis B | Other diseases of digestive system | 20 | 2.25E-08 | 3.59 (2.22, 5.47) |
| C34 | C80 | Malignant neoplasm of bronchus and lung | Malignant neoplasm, without specification of site | 20 | 1.71E-54 | 50.61 (30.06, 81.11) |
| C67 | N40 | Malignant neoplasm of bladder | Hyperplasia of prostate | 20 | 3.50E-17 | 9.09 (5.32, 14.88) |
| D24 | N64 | Benign neoplasm of breast | Other disorders of breast | 20 | 4.11E-22 | 10.48 (6.32, 16.46) |
| D25 | D39 | Leiomyoma of uterus | Neoplasm of uncertain or unknown behaviour of female genital organs | 20 | 2.80E-07 | 3.68 (2.18, 5.93) |
| D69 | K74 | Purpura and other haemorrhagic conditions | Fibrosis and cirrhosis of liver | 20 | 8.65E-09 | 3.73 (2.3, 5.69) |
| D86 | J43 | Sarcoidosis | Emphysema | 20 | 6.41E-26 | 12.42 (7.54, 19.34) |
| E16 | E66 | Other disorders of pancreatic internal secretion | Obesity | 20 | 4.16E-65 | 64.12 (38.61, 100.95) |
| E72 | F32 | Other disorders of amino-acid metabolism | Depressive episode | 20 | 1.53E-07 | 3.39 (2.08, 5.21) |
| E77 | K56 | Disorders of glycoprotein metabolism | Paralytic ileus and intestinal obstruction without hernia | 20 | 1.22E-09 | 4.01 (2.48, 6.1) |
| E77 | N18 | Disorders of glycoprotein metabolism | Chronic kidney disease | 20 | 9.74E-13 | 5.13 (3.17, 7.82) |
| E78 | L28 | Disorders of lipoprotein metabolism and other lipidaemias | Lichen simplex chronicus and prurigo | 20 | 9.46E-07 | 3.55 (2.09, 5.8) |
| E79 | I48 | Disorders of purine and pyrimidine metabolism | Atrial fibrillation and flutter | 20 | 3.91E-09 | 3.88 (2.39, 5.92) |
| G43 | I65 | Migraine | Occlusion and stenosis of precerebral arteries, not resulting in cerebral infarction | 20 | 1.37E-10 | 4.51 (2.76, 6.93) |
| G55 | M81 | Nerve root and plexus compressions in diseases classified elsewhere | Osteoporosis without pathological fracture | 20 | 5.58E-08 | 3.47 (2.14, 5.29) |
| G81 | I61 | Hemiplegia | Intracerebral haemorrhage | 20 | 2.08E-46 | 57.14 (32.27, 98.34) |
| G93 | I62 | Other disorders of brain | Other nontraumatic intracranial haemorrhage | 20 | 6.58E-41 | 24.64 (14.96, 38.4) |
| H21 | H43 | Other disorders of iris and ciliary body | Disorders of vitreous body | 20 | 3.27E-73 | 108.33 (63.62, 176.45) |
| H35 | H40 | Other retinal disorders | Glaucoma | 20 | 1.78E-29 | 13.85 (8.49, 21.28) |
| H52 | H53 | Disorders of refraction and accommodation | Visual disturbances | 20 | 6.75E-63 | 78.61 (46.04, 128.65) |
| I12 | N03 | Hypertensive renal disease | Chronic nephritic syndrome | 20 | 1.96E-38 | 21.26 (12.98, 32.88) |
| I60 | J94 | Subarachnoid haemorrhage | Other pleural conditions | 20 | 1.86E-09 | 3.97 (2.45, 6.06) |
| I86 | N50 | Varicose veins of other sites | Other disorders of male genital organs | 20 | 2.93E-67 | 78.16 (46.47, 125.15) |
| J98 | K72 | Other respiratory disorders | Hepatic failure, not elsewhere classified | 20 | 3.07E-12 | 5.1 (3.12, 7.85) |
| K82 | K83 | Other diseases of gallbladder | Other diseases of biliary tract | 20 | 1.07E-06 | 3.05 (1.88, 4.66) |
| M10 | M65 | Gout | Synovitis and tenosynovitis | 20 | 2.42E-23 | 10.19 (6.25, 15.67) |
| M41 | M51 | Scoliosis | Other intervertebral disc disorders | 20 | 2.55E-14 | 7.72 (4.46, 12.87) |
| N28 | N30 | Other disorders of kidney and ureter, not elsewhere classified | Cystitis | 20 | 2.52E-09 | 3.96 (2.44, 6.07) |
| A49 | K21 | Bacterial infection of unspecified site | Gastro-oesophageal reflux disease | 19 | 6.17E-09 | 3.91 (2.38, 6.02) |
| A54 | N76 | Gonococcal infection | Other inflammation of vagina and vulva | 19 | 1.45E-37 | 93.23 (46.88, 187.51) |
| A98 | I40 | Other viral haemorrhagic fevers, not elsewhere classified | Acute myocarditis | 19 | 6.30E-74 | 118.45 (68.94, 193.57) |
| B37 | N76 | Candidiasis | Other inflammation of vagina and vulva | 19 | 2.35E-20 | 10.3 (6.1, 16.46) |
| C77 | C78 | Secondary and unspecified malignant neoplasm of lymph nodes | Secondary malignant neoplasm of respiratory and digestive organs | 19 | 5.93E-83 | 150.16 (87.74, 244.17) |
| E11 | N26 | Type 2 diabetes mellitus | Unspecified contracted kidney | 19 | 8.37E-09 | 5.32 (2.96, 9.26) |
| E66 | E79 | Obesity | Disorders of purine and pyrimidine metabolism | 19 | 2.39E-21 | 9.99 (6.02, 15.61) |
| E72 | E83 | Other disorders of amino-acid metabolism | Disorders of mineral metabolism | 19 | 1.26E-06 | 3.19 (1.93, 4.96) |
| H20 | H52 | Iridocyclitis | Disorders of refraction and accommodation | 19 | 9.93E-33 | 17.72 (10.68, 27.64) |
| H35 | N18 | Other retinal disorders | Chronic kidney disease | 19 | 1.70E-14 | 6.09 (3.71, 9.38) |
| H50 | H52 | Other strabismus | Disorders of refraction and accommodation | 19 | 1.43E-63 | 112.26 (63.43, 191.71) |
| H65 | H91 | Nonsuppurative otitis media | Other hearing loss | 19 | 5.85E-28 | 13.67 (8.28, 21.21) |
| I20 | I97 | Angina pectoris | Postprocedural disorders of circulatory system, not elsewhere classified | 19 | 2.08E-17 | 9.42 (5.48, 15.45) |
| I27 | J98 | Other pulmonary heart diseases | Other respiratory disorders | 19 | 3.37E-09 | 4.09 (2.48, 6.35) |
| I40 | N08 | Acute myocarditis | Glomerular disorders in diseases classified elsewhere | 19 | 1.96E-25 | 12.86 (7.71, 20.23) |
| I42 | I44 | Cardiomyopathy | Atrioventricular and left bundle-branch block | 19 | 9.03E-35 | 19.76 (11.89, 30.9) |
| I42 | I51 | Cardiomyopathy | Complications and ill-defined descriptions of heart disease | 19 | 4.25E-10 | 4.37 (2.66, 6.75) |
| I42 | J94 | Cardiomyopathy | Other pleural conditions | 19 | 1.04E-10 | 4.6 (2.8, 7.11) |
| I45 | I51 | Other conduction disorders | Complications and ill-defined descriptions of heart disease | 19 | 2.29E-07 | 3.37 (2.05, 5.19) |
| I48 | J94 | Atrial fibrillation and flutter | Other pleural conditions | 19 | 7.04E-08 | 3.55 (2.16, 5.47) |
| J02 | J18 | Acute pharyngitis | Pneumonia, organism unspecified | 19 | 8.89E-07 | 3.22 (1.95, 4.99) |
| J03 | J31 | Acute tonsillitis | Chronic rhinitis, nasopharyngitis and pharyngitis | 19 | 3.66E-07 | 3.3 (2.01, 5.07) |
| J04 | J38 | Acute laryngitis and tracheitis | Diseases of vocal cords and larynx, not elsewhere classified | 19 | 2.79E-29 | 14.83 (8.96, 23.08) |
| J45 | J47 | Asthma | Bronchiectasis | 19 | 2.62E-13 | 5.58 (3.4, 8.6) |
| K60 | L30 | Fissure and fistula of anal and rectal regions | Other dermatitis | 19 | 4.83E-08 | 3.66 (2.22, 5.65) |
| N21 | N39 | Calculus of lower urinary tract | Other disorders of urinary system | 19 | 5.02E-25 | 12.43 (7.46, 19.52) |
| N81 | N84 | Female genital prolapse | Polyp of female genital tract | 19 | 9.94E-09 | 3.94 (2.38, 6.11) |

**Table S6. The odds ratios (ORs) and frequency of the statistically significant (P value, Bonferroni correction) and common (prevalence >1/10000) comorbidity patterns included in the complete multimorbidity network in the UK overall inpatients (467 patterns).**

| Source | Target | SourceName | TargetName | Frequency | P | OR(95%CI) |
| --- | --- | --- | --- | --- | --- | --- |
| B96 | K21 | Other specified bacterial agents as the cause of diseases classified to other chapters | Gastro-oesophageal reflux disease | 28 | 5.30E-12 | 3.92 (2.6, 5.68) |
| B96 | K26 | Other specified bacterial agents as the cause of diseases classified to other chapters | Duodenal ulcer | 34 | 2.60E-66 | 24.56 (16.77, 34.86) |
| B96 | K29 | Other specified bacterial agents as the cause of diseases classified to other chapters | Gastritis and duodenitis | 139 | 2.70E-157 | 19.98 (16.01, 24.86) |
| B96 | K44 | Other specified bacterial agents as the cause of diseases classified to other chapters | Diaphragmatic hernia | 47 | 4.67E-25 | 5.13 (3.72, 6.92) |
| B96 | N39 | Other specified bacterial agents as the cause of diseases classified to other chapters | Other disorders of urinary system | 61 | 2.78E-63 | 11.48 (8.56, 15.15) |
| C44 | M80 | Other malignant neoplasms of skin | Osteoporosis with pathological fracture | 25 | 2.47E-66 | 45.48 (28.83, 68.85) |
| C50 | C77 | Malignant neoplasm of breast | Secondary and unspecified malignant neoplasm of lymph nodes | 183 | 0 | 40.5 (33.69, 48.53) |
| C50 | D05 | Malignant neoplasm of breast | Carcinoma in situ of breast | 45 | 2.38E-51 | 11.37 (8.18, 15.42) |
| C77 | C80 | Secondary and unspecified malignant neoplasm of lymph nodes | Malignant neoplasm, without specification of site | 23 | 5.41E-68 | 85.51 (50.93, 139.14) |
| C77 | D05 | Secondary and unspecified malignant neoplasm of lymph nodes | Carcinoma in situ of breast | 38 | 7.87E-58 | 16.65 (11.62, 23.16) |
| C78 | C79 | Secondary malignant neoplasm of respiratory and digestive organs | Secondary malignant neoplasm of other and unspecified sites | 29 | 9.63E-150 | 464.07 (288.68, 729.02) |
| C78 | C80 | Secondary malignant neoplasm of respiratory and digestive organs | Malignant neoplasm, without specification of site | 21 | 8.84E-111 | 396.09 (229.79, 658.48) |
| D05 | N60 | Carcinoma in situ of breast | Benign mammary dysplasia | 26 | 5.99E-30 | 10.51 (6.84, 15.45) |
| D12 | I84 | Benign neoplasm of colon, rectum, anus and anal canal | Haemorrhoids | 76 | 1.49E-15 | 2.61 (2.04, 3.28) |
| D12 | K57 | Benign neoplasm of colon, rectum, anus and anal canal | Diverticular disease of intestine | 134 | 3.88E-86 | 6.43 (5.32, 7.71) |
| D12 | K62 | Benign neoplasm of colon, rectum, anus and anal canal | Other diseases of anus and rectum | 143 | 1.78E-60 | 4.45 (3.71, 5.3) |
| D12 | K63 | Benign neoplasm of colon, rectum, anus and anal canal | Other diseases of intestine | 44 | 4.50E-20 | 4.21 (3.05, 5.65) |
| D13 | K21 | Benign neoplasm of other and ill-defined parts of digestive system | Gastro-oesophageal reflux disease | 20 | 4.92E-15 | 6.61 (4, 10.35) |
| D13 | K29 | Benign neoplasm of other and ill-defined parts of digestive system | Gastritis and duodenitis | 47 | 8.57E-47 | 12.8 (8.96, 18.01) |
| D13 | K44 | Benign neoplasm of other and ill-defined parts of digestive system | Diaphragmatic hernia | 32 | 2.19E-26 | 8.43 (5.6, 12.33) |
| D17 | K40 | Benign lipomatous neoplasm | Inguinal hernia | 83 | 9.96E-29 | 3.84 (3.01, 4.84) |
| D22 | D23 | Melanocytic naevi | Other benign neoplasms of skin | 23 | 5.06E-23 | 8.42 (5.36, 12.54) |
| D22 | L82 | Melanocytic naevi | Seborrhoeic keratosis | 21 | 1.24E-19 | 7.68 (4.79, 11.63) |
| D24 | N60 | Benign neoplasm of breast | Benign mammary dysplasia | 22 | 7.13E-31 | 13.96 (8.69, 21.32) |
| D25 | D27 | Leiomyoma of uterus | Benign neoplasm of ovary | 42 | 2.08E-52 | 14.95 (10.44, 20.97) |
| D25 | D50 | Leiomyoma of uterus | Iron deficiency anaemia | 38 | 3.07E-13 | 3.51 (2.47, 4.83) |
| D25 | D64 | Leiomyoma of uterus | Other anaemias | 36 | 2.53E-07 | 2.46 (1.72, 3.4) |
| D25 | M88 | Leiomyoma of uterus | Paget disease of bone [osteitis deformans] | 34 | 1.54E-49 | 24.59 (15.96, 37.23) |
| D25 | N72 | Leiomyoma of uterus | Inflammatory disease of cervix uteri | 55 | 8.15E-37 | 6.4 (4.75, 8.45) |
| D25 | N73 | Leiomyoma of uterus | Other female pelvic inflammatory diseases | 54 | 2.83E-21 | 4.02 (2.99, 5.29) |
| D25 | N80 | Leiomyoma of uterus | Endometriosis | 196 | 1.99E-148 | 8.77 (7.43, 10.29) |
| D25 | N81 | Leiomyoma of uterus | Female genital prolapse | 122 | 3.50E-19 | 2.36 (1.94, 2.83) |
| D25 | N83 | Leiomyoma of uterus | Noninflammatory disorders of ovary, fallopian tube and broad ligament | 230 | 5.81E-166 | 8.07 (6.94, 9.35) |
| D25 | N84 | Leiomyoma of uterus | Polyp of female genital tract | 204 | 6.74E-35 | 2.54 (2.19, 2.94) |
| D25 | N85 | Leiomyoma of uterus | Other noninflammatory disorders of uterus, except cervix | 88 | 2.11E-18 | 2.67 (2.13, 3.3) |
| D25 | N88 | Leiomyoma of uterus | Other noninflammatory disorders of cervix uteri | 34 | 1.81E-08 | 2.72 (1.88, 3.79) |
| D25 | N92 | Leiomyoma of uterus | Excessive, frequent and irregular menstruation | 443 | 1.00E-95 | 3.37 (3.01, 3.77) |
| D25 | N94 | Leiomyoma of uterus | Pain and other conditions associated with female genital organs and menstrual cycle | 60 | 9.39E-08 | 2.12 (1.61, 2.75) |
| D25 | N95 | Leiomyoma of uterus | Menopausal and other perimenopausal disorders | 122 | 6.36E-10 | 1.83 (1.51, 2.2) |
| D27 | N80 | Benign neoplasm of ovary | Endometriosis | 29 | 4.95E-41 | 17.17 (11.15, 25.57) |
| D27 | N83 | Benign neoplasm of ovary | Noninflammatory disorders of ovary, fallopian tube and broad ligament | 35 | 6.95E-45 | 14.87 (10.06, 21.38) |
| D50 | K25 | Iron deficiency anaemia | Gastric ulcer | 20 | 2.57E-17 | 7.07 (4.35, 10.8) |
| D50 | K29 | Iron deficiency anaemia | Gastritis and duodenitis | 82 | 9.47E-30 | 3.83 (3.01, 4.8) |
| D50 | K31 | Iron deficiency anaemia | Other diseases of stomach and duodenum | 24 | 4.92E-23 | 8.08 (5.2, 11.95) |
| D50 | K44 | Iron deficiency anaemia | Diaphragmatic hernia | 47 | 3.34E-08 | 2.31 (1.7, 3.08) |
| D50 | N92 | Iron deficiency anaemia | Excessive, frequent and irregular menstruation | 114 | 3.65E-23 | 3.05 (2.45, 3.77) |
| D64 | K25 | Other anaemias | Gastric ulcer | 20 | 1.89E-11 | 4.66 (2.88, 7.1) |
| D64 | K29 | Other anaemias | Gastritis and duodenitis | 117 | 2.66E-39 | 3.67 (3.01, 4.44) |
| D64 | K44 | Other anaemias | Diaphragmatic hernia | 72 | 8.29E-13 | 2.41 (1.88, 3.05) |
| D64 | K92 | Other anaemias | Other diseases of digestive system | 39 | 4.74E-24 | 5.37 (3.82, 7.34) |
| E03 | E11 | Other hypothyroidism | Type 2 diabetes mellitus | 46 | 8.51E-23 | 4.55 (3.32, 6.09) |
| E03 | E66 | Other hypothyroidism | Obesity | 29 | 4.52E-17 | 5.05 (3.38, 7.22) |
| E03 | E78 | Other hypothyroidism | Disorders of lipoprotein metabolism and other lipidaemias | 62 | 9.74E-34 | 5.16 (3.92, 6.66) |
| E03 | F32 | Other hypothyroidism | Depressive episode | 34 | 6.78E-16 | 4.21 (2.91, 5.86) |
| E03 | I10 | Other hypothyroidism | Essential (primary) hypertension | 207 | 1.87E-59 | 3.61 (3.09, 4.21) |
| E03 | J45 | Other hypothyroidism | Asthma | 103 | 3.64E-23 | 2.83 (2.29, 3.45) |
| E03 | M13 | Other hypothyroidism | Other arthritis | 20 | 1.35E-06 | 3.04 (1.87, 4.63) |
| E05 | H06 | Thyrotoxicosis [hyperthyroidism] | Disorders of lacrimal system and orbit in diseases classified elsewhere | 21 | 8.45E-71 | 1641.09 (749.11, 3892.86) |
| E05 | I10 | Thyrotoxicosis [hyperthyroidism] | Essential (primary) hypertension | 39 | 1.32E-10 | 3.13 (2.18, 4.38) |
| E10 | E16 | Type 1 diabetes mellitus | Other disorders of pancreatic internal secretion | 27 | 1.76E-96 | 143.87 (88.91, 227.22) |
| E10 | E78 | Type 1 diabetes mellitus | Disorders of lipoprotein metabolism and other lipidaemias | 40 | 2.22E-21 | 4.88 (3.47, 6.69) |
| E10 | H36 | Type 1 diabetes mellitus | Retinal disorders in diseases classified elsewhere | 54 | 2.54E-169 | 85.8 (62.11, 116.67) |
| E10 | I10 | Type 1 diabetes mellitus | Essential (primary) hypertension | 109 | 3.40E-33 | 3.69 (2.97, 4.55) |
| E10 | I20 | Type 1 diabetes mellitus | Angina pectoris | 29 | 2.79E-10 | 3.38 (2.26, 4.85) |
| E11 | E66 | Type 2 diabetes mellitus | Obesity | 92 | 7.09E-88 | 9.53 (7.58, 11.84) |
| E11 | E78 | Type 2 diabetes mellitus | Disorders of lipoprotein metabolism and other lipidaemias | 331 | 2.49E-288 | 10.39 (9.14, 11.77) |
| E11 | F17 | Type 2 diabetes mellitus | Mental and behavioural disorders due to use of tobacco | 30 | 2.11E-06 | 2.45 (1.66, 3.48) |
| E11 | F32 | Type 2 diabetes mellitus | Depressive episode | 49 | 1.39E-16 | 3.41 (2.52, 4.51) |
| E11 | H36 | Type 2 diabetes mellitus | Retinal disorders in diseases classified elsewhere | 82 | 3.94E-143 | 34.12 (25.9, 44.59) |
| E11 | I10 | Type 2 diabetes mellitus | Essential (primary) hypertension | 818 | 0 | 8.3 (7.57, 9.09) |
| E11 | I20 | Type 2 diabetes mellitus | Angina pectoris | 130 | 3.58E-35 | 3.19 (2.64, 3.82) |
| E11 | I21 | Type 2 diabetes mellitus | Acute myocardial infarction | 71 | 4.76E-08 | 1.97 (1.53, 2.49) |
| E11 | I25 | Type 2 diabetes mellitus | Chronic ischaemic heart disease | 144 | 4.05E-21 | 2.32 (1.94, 2.76) |
| E11 | J18 | Type 2 diabetes mellitus | Pneumonia, organism unspecified | 30 | 2.14E-07 | 2.65 (1.8, 3.77) |
| E11 | J45 | Type 2 diabetes mellitus | Asthma | 134 | 2.56E-14 | 1.99 (1.66, 2.37) |
| E11 | L03 | Type 2 diabetes mellitus | Cellulitis | 44 | 5.52E-08 | 2.34 (1.7, 3.13) |
| E14 | E78 | Unspecified diabetes mellitus | Disorders of lipoprotein metabolism and other lipidaemias | 21 | 3.24E-08 | 3.53 (2.19, 5.39) |
| E14 | H36 | Unspecified diabetes mellitus | Retinal disorders in diseases classified elsewhere | 66 | 1.01E-248 | 196.47 (143.71, 265.9) |
| E14 | I10 | Unspecified diabetes mellitus | Essential (primary) hypertension | 51 | 3.42E-08 | 2.34 (1.71, 3.14) |
| E16 | I10 | Other disorders of pancreatic internal secretion | Essential (primary) hypertension | 24 | 1.35E-12 | 5.78 (3.5, 9.26) |
| E66 | E78 | Obesity | Disorders of lipoprotein metabolism and other lipidaemias | 110 | 5.44E-99 | 9.3 (7.52, 11.38) |
| E66 | F17 | Obesity | Mental and behavioural disorders due to use of tobacco | 39 | 2.01E-41 | 9.64 (6.82, 13.21) |
| E66 | F32 | Obesity | Depressive episode | 30 | 1.29E-16 | 4.72 (3.2, 6.7) |
| E66 | G47 | Obesity | Sleep disorders | 64 | 1.14E-89 | 15.24 (11.58, 19.73) |
| E66 | I10 | Obesity | Essential (primary) hypertension | 301 | 1.62E-170 | 7.57 (6.55, 8.72) |
| E66 | I20 | Obesity | Angina pectoris | 38 | 2.00E-09 | 2.74 (1.94, 3.76) |
| E66 | I25 | Obesity | Chronic ischaemic heart disease | 55 | 1.82E-14 | 3 (2.24, 3.93) |
| E66 | J45 | Obesity | Asthma | 78 | 1.11E-16 | 2.68 (2.1, 3.36) |
| E66 | M13 | Obesity | Other arthritis | 24 | 2.65E-15 | 5.26 (3.39, 7.75) |
| E66 | M17 | Obesity | Gonarthrosis [arthrosis of knee] | 57 | 5.93E-21 | 3.66 (2.76, 4.76) |
| E66 | M19 | Obesity | Other arthrosis | 26 | 1.76E-08 | 3.1 (2.04, 4.5) |
| E78 | F17 | Disorders of lipoprotein metabolism and other lipidaemias | Mental and behavioural disorders due to use of tobacco | 78 | 4.00E-43 | 5.36 (4.19, 6.76) |
| E78 | F32 | Disorders of lipoprotein metabolism and other lipidaemias | Depressive episode | 50 | 5.76E-12 | 2.76 (2.04, 3.64) |
| E78 | F41 | Disorders of lipoprotein metabolism and other lipidaemias | Other anxiety disorders | 28 | 1.62E-08 | 3.04 (2.02, 4.38) |
| E78 | G45 | Disorders of lipoprotein metabolism and other lipidaemias | Transient cerebral ischaemic attacks and related syndromes | 25 | 2.04E-12 | 4.48 (2.88, 6.64) |
| E78 | H36 | Disorders of lipoprotein metabolism and other lipidaemias | Retinal disorders in diseases classified elsewhere | 19 | 4.44E-09 | 4.13 (2.49, 6.44) |
| E78 | I10 | Disorders of lipoprotein metabolism and other lipidaemias | Essential (primary) hypertension | 1312 | 0 | 13.1 (12.1, 14.18) |
| E78 | I20 | Disorders of lipoprotein metabolism and other lipidaemias | Angina pectoris | 459 | 0 | 11.6 (10.37, 12.95) |
| E78 | I21 | Disorders of lipoprotein metabolism and other lipidaemias | Acute myocardial infarction | 313 | 1.30E-229 | 8.5 (7.46, 9.67) |
| E78 | I25 | Disorders of lipoprotein metabolism and other lipidaemias | Chronic ischaemic heart disease | 617 | 0 | 11.33 (10.25, 12.52) |
| E78 | I44 | Disorders of lipoprotein metabolism and other lipidaemias | Atrioventricular and left bundle-branch block | 24 | 1.78E-11 | 4.28 (2.73, 6.4) |
| E78 | I48 | Disorders of lipoprotein metabolism and other lipidaemias | Atrial fibrillation and flutter | 72 | 6.53E-10 | 2.14 (1.67, 2.71) |
| E78 | I50 | Disorders of lipoprotein metabolism and other lipidaemias | Heart failure | 39 | 3.71E-18 | 4.42 (3.11, 6.09) |
| E78 | I51 | Disorders of lipoprotein metabolism and other lipidaemias | Complications and ill-defined descriptions of heart disease | 20 | 2.27E-07 | 3.35 (2.05, 5.17) |
| E78 | I63 | Disorders of lipoprotein metabolism and other lipidaemias | Cerebral infarction | 55 | 2.00E-45 | 8.21 (6.07, 10.89) |
| E78 | I73 | Disorders of lipoprotein metabolism and other lipidaemias | Other peripheral vascular diseases | 37 | 1.95E-23 | 5.92 (4.12, 8.25) |
| E78 | J45 | Disorders of lipoprotein metabolism and other lipidaemias | Asthma | 151 | 1.56E-11 | 1.78 (1.5, 2.09) |
| E78 | M10 | Disorders of lipoprotein metabolism and other lipidaemias | Gout | 32 | 1.28E-11 | 3.55 (2.41, 5.03) |
| E87 | I10 | Other disorders of fluid, electrolyte and acid-base balance | Essential (primary) hypertension | 65 | 3.48E-29 | 5.36 (3.97, 7.15) |
| E87 | J18 | Other disorders of fluid, electrolyte and acid-base balance | Pneumonia, organism unspecified | 20 | 4.42E-35 | 18.77 (11.43, 29.12) |
| E87 | N17 | Other disorders of fluid, electrolyte and acid-base balance | Acute renal failure | 31 | 2.54E-119 | 131.24 (85.64, 195.38) |
| F10 | F17 | Mental and behavioural disorders due to use of alcohol | Mental and behavioural disorders due to use of tobacco | 41 | 4.37E-41 | 9.24 (6.58, 12.62) |
| F10 | F32 | Mental and behavioural disorders due to use of alcohol | Depressive episode | 63 | 1.45E-74 | 11.97 (9.09, 15.49) |
| F10 | I10 | Mental and behavioural disorders due to use of alcohol | Essential (primary) hypertension | 110 | 8.09E-12 | 2.03 (1.65, 2.48) |
| F10 | I48 | Mental and behavioural disorders due to use of alcohol | Atrial fibrillation and flutter | 28 | 7.41E-08 | 2.86 (1.91, 4.11) |
| F10 | K70 | Mental and behavioural disorders due to use of alcohol | Alcoholic liver disease | 49 | 3.19E-124 | 90.72 (62.19, 131.18) |
| F10 | K92 | Mental and behavioural disorders due to use of alcohol | Other diseases of digestive system | 44 | 6.50E-28 | 5.68 (4.1, 7.64) |
| F17 | F32 | Mental and behavioural disorders due to use of tobacco | Depressive episode | 52 | 6.31E-54 | 9.67 (7.17, 12.76) |
| F17 | F41 | Mental and behavioural disorders due to use of tobacco | Other anxiety disorders | 23 | 1.93E-22 | 8.24 (5.24, 12.29) |
| F17 | I10 | Mental and behavioural disorders due to use of tobacco | Essential (primary) hypertension | 161 | 2.09E-34 | 2.99 (2.5, 3.55) |
| F17 | I21 | Mental and behavioural disorders due to use of tobacco | Acute myocardial infarction | 84 | 3.57E-59 | 6.99 (5.49, 8.79) |
| F17 | I25 | Mental and behavioural disorders due to use of tobacco | Chronic ischaemic heart disease | 79 | 6.30E-25 | 3.54 (2.77, 4.47) |
| F17 | J18 | Mental and behavioural disorders due to use of tobacco | Pneumonia, organism unspecified | 22 | 1.51E-13 | 5.06 (3.19, 7.59) |
| F17 | J44 | Mental and behavioural disorders due to use of tobacco | Other chronic obstructive pulmonary disease | 31 | 1.51E-39 | 12.41 (8.38, 17.73) |
| F32 | F41 | Depressive episode | Other anxiety disorders | 101 | 2.38E-197 | 29.36 (23.42, 36.46) |
| F32 | I10 | Depressive episode | Essential (primary) hypertension | 191 | 4.65E-35 | 2.7 (2.3, 3.15) |
| F32 | J45 | Depressive episode | Asthma | 100 | 1.36E-17 | 2.45 (1.98, 2.99) |
| F32 | M13 | Depressive episode | Other arthritis | 19 | 2.81E-06 | 2.99 (1.82, 4.58) |
| F41 | I10 | Other anxiety disorders | Essential (primary) hypertension | 115 | 1.03E-29 | 3.31 (2.68, 4.05) |
| F41 | J45 | Other anxiety disorders | Asthma | 45 | 1.00E-07 | 2.28 (1.66, 3.06) |
| G45 | I10 | Transient cerebral ischaemic attacks and related syndromes | Essential (primary) hypertension | 75 | 2.10E-24 | 3.99 (3.04, 5.18) |
| G47 | I10 | Sleep disorders | Essential (primary) hypertension | 109 | 1.53E-06 | 1.65 (1.34, 2.01) |
| G55 | M47 | Nerve root and plexus compressions in diseases classified elsewhere | Spondylosis | 55 | 1.74E-120 | 32.48 (24.01, 43.13) |
| G55 | M48 | Nerve root and plexus compressions in diseases classified elsewhere | Other spondylopathies | 48 | 3.10E-144 | 64.61 (46.44, 88.09) |
| G55 | M50 | Nerve root and plexus compressions in diseases classified elsewhere | Cervical disc disorders | 45 | 1.18E-149 | 91.01 (64.15, 126.55) |
| G55 | M51 | Nerve root and plexus compressions in diseases classified elsewhere | Other intervertebral disc disorders | 353 | 0 | 560.26 (445.57, 710.74) |
| G56 | M65 | Mononeuropathies of upper limb | Synovitis and tenosynovitis | 34 | 6.70E-10 | 2.97 (2.06, 4.12) |
| G57 | M20 | Mononeuropathies of lower limb | Acquired deformities of fingers and toes | 19 | 6.56E-13 | 5.8 (3.48, 9.12) |
| G81 | I10 | Hemiplegia | Essential (primary) hypertension | 39 | 4.85E-14 | 4.05 (2.78, 5.76) |
| G81 | I63 | Hemiplegia | Cerebral infarction | 32 | 6.69E-109 | 97.46 (64.05, 144.26) |
| G81 | I69 | Hemiplegia | Sequelae of cerebrovascular disease | 29 | 9.84E-149 | 920.52 (546.62, 1537.13) |
| G91 | I60 | Hydrocephalus | Subarachnoid haemorrhage | 21 | 1.40E-106 | 487.46 (276.09, 839.57) |
| G99 | M47 | Other disorders of nervous system in diseases classified elsewhere | Spondylosis | 31 | 1.61E-95 | 90.54 (58.35, 137.19) |
| G99 | M50 | Other disorders of nervous system in diseases classified elsewhere | Cervical disc disorders | 42 | 2.79E-188 | 488.17 (320.74, 735.99) |
| G99 | M51 | Other disorders of nervous system in diseases classified elsewhere | Other intervertebral disc disorders | 37 | 3.26E-87 | 58.53 (38.75, 86.95) |
| H00 | H01 | Hordeolum and chalazion | Other inflammation of eyelid | 28 | 1.02E-109 | 158.6 (99.98, 244.79) |
| H25 | H40 | Senile cataract | Glaucoma | 26 | 2.05E-36 | 13.62 (8.86, 20.03) |
| H25 | H52 | Senile cataract | Disorders of refraction and accommodation | 40 | 4.33E-96 | 39.03 (27.26, 54.48) |
| H26 | H40 | Other cataract | Glaucoma | 33 | 1.35E-26 | 7 (4.81, 9.85) |
| H26 | H52 | Other cataract | Disorders of refraction and accommodation | 41 | 1.47E-58 | 15.35 (10.86, 21.13) |
| H33 | H35 | Retinal detachments and breaks | Other retinal disorders | 68 | 5.33E-164 | 45.41 (34.29, 59.33) |
| H33 | H43 | Retinal detachments and breaks | Disorders of vitreous body | 101 | 6.38E-297 | 182.86 (138.41, 241.1) |
| H33 | H44 | Retinal detachments and breaks | Disorders of globe | 22 | 6.81E-64 | 77.1 (45.68, 125.72) |
| H33 | H52 | Retinal detachments and breaks | Disorders of refraction and accommodation | 99 | 1.05E-279 | 89.49 (69.69, 114.11) |
| H35 | H36 | Other retinal disorders | Retinal disorders in diseases classified elsewhere | 21 | 1.33E-54 | 38.61 (23.69, 59.71) |
| H35 | H43 | Other retinal disorders | Disorders of vitreous body | 36 | 1.01E-118 | 82.94 (56.29, 119.04) |
| H35 | H52 | Other retinal disorders | Disorders of refraction and accommodation | 42 | 4.69E-124 | 60.37 (42.46, 83.82) |
| H36 | I10 | Retinal disorders in diseases classified elsewhere | Essential (primary) hypertension | 56 | 4.73E-17 | 3.63 (2.67, 4.88) |
| H40 | H52 | Glaucoma | Disorders of refraction and accommodation | 23 | 9.98E-42 | 19.8 (12.51, 29.82) |
| H43 | H52 | Disorders of vitreous body | Disorders of refraction and accommodation | 51 | 4.82E-190 | 150.27 (106.72, 208.38) |
| H50 | H53 | Other strabismus | Visual disturbances | 20 | 1.03E-61 | 55.99 (33.78, 87.95) |
| I08 | I10 | Multiple valve diseases | Essential (primary) hypertension | 26 | 1.46E-12 | 5.46 (3.36, 8.64) |
| I08 | I25 | Multiple valve diseases | Chronic ischaemic heart disease | 19 | 1.07E-17 | 10.57 (6.02, 17.79) |
| I10 | I20 | Essential (primary) hypertension | Angina pectoris | 703 | 4.80E-224 | 4.29 (3.92, 4.69) |
| I10 | I21 | Essential (primary) hypertension | Acute myocardial infarction | 424 | 7.49E-81 | 2.91 (2.6, 3.25) |
| I10 | I25 | Essential (primary) hypertension | Chronic ischaemic heart disease | 909 | 1.41E-240 | 3.81 (3.52, 4.12) |
| I10 | I35 | Essential (primary) hypertension | Nonrheumatic aortic valve disorders | 51 | 2.47E-11 | 2.88 (2.09, 3.89) |
| I10 | I42 | Essential (primary) hypertension | Cardiomyopathy | 31 | 1.57E-08 | 3.18 (2.09, 4.68) |
| I10 | I44 | Essential (primary) hypertension | Atrioventricular and left bundle-branch block | 55 | 2.50E-11 | 2.75 (2.02, 3.68) |
| I10 | I48 | Essential (primary) hypertension | Atrial fibrillation and flutter | 249 | 6.79E-27 | 2.13 (1.85, 2.44) |
| I10 | I50 | Essential (primary) hypertension | Heart failure | 96 | 5.38E-24 | 3.29 (2.6, 4.12) |
| I10 | I51 | Essential (primary) hypertension | Complications and ill-defined descriptions of heart disease | 64 | 1.53E-16 | 3.26 (2.44, 4.29) |
| I10 | I61 | Essential (primary) hypertension | Intracerebral haemorrhage | 40 | 1.53E-20 | 6.25 (4.21, 9.13) |
| I10 | I63 | Essential (primary) hypertension | Cerebral infarction | 112 | 2.34E-46 | 5.11 (4.07, 6.38) |
| I10 | I64 | Essential (primary) hypertension | Stroke, not specified as haemorrhage or infarction | 65 | 5.10E-29 | 5.49 (4.05, 7.37) |
| I10 | I65 | Essential (primary) hypertension | Occlusion and stenosis of precerebral arteries, not resulting in cerebral infarction | 26 | 2.29E-10 | 4.43 (2.75, 6.93) |
| I10 | I67 | Essential (primary) hypertension | Other cerebrovascular diseases | 35 | 5.71E-14 | 4.52 (3.01, 6.62) |
| I10 | I69 | Essential (primary) hypertension | Sequelae of cerebrovascular disease | 21 | 9.94E-12 | 6.23 (3.61, 10.41) |
| I10 | I73 | Essential (primary) hypertension | Other peripheral vascular diseases | 61 | 9.30E-11 | 2.53 (1.9, 3.32) |
| I10 | J44 | Essential (primary) hypertension | Other chronic obstructive pulmonary disease | 77 | 1.73E-10 | 2.24 (1.74, 2.86) |
| I10 | J45 | Essential (primary) hypertension | Asthma | 800 | 4.60E-114 | 2.47 (2.28, 2.67) |
| I10 | J96 | Essential (primary) hypertension | Respiratory failure, not elsewhere classified | 23 | 1.53E-07 | 3.52 (2.15, 5.54) |
| I10 | K76 | Essential (primary) hypertension | Other diseases of liver | 50 | 7.54E-09 | 2.46 (1.79, 3.32) |
| I10 | L40 | Essential (primary) hypertension | Psoriasis | 70 | 6.59E-09 | 2.13 (1.63, 2.73) |
| I10 | M10 | Essential (primary) hypertension | Gout | 112 | 7.76E-39 | 4.39 (3.5, 5.46) |
| I10 | M13 | Essential (primary) hypertension | Other arthritis | 150 | 8.79E-29 | 2.75 (2.3, 3.28) |
| I10 | M15 | Essential (primary) hypertension | Polyarthrosis | 41 | 2.46E-09 | 2.84 (1.99, 3.95) |
| I10 | N17 | Essential (primary) hypertension | Acute renal failure | 44 | 7.33E-11 | 3.06 (2.16, 4.24) |
| I10 | N18 | Essential (primary) hypertension | Chronic kidney disease | 50 | 1.00E-19 | 4.54 (3.24, 6.24) |
| I10 | N19 | Essential (primary) hypertension | Unspecified kidney failure | 25 | 1.80E-09 | 4.03 (2.51, 6.24) |
| I20 | I21 | Angina pectoris | Acute myocardial infarction | 122 | 5.14E-23 | 2.59 (2.13, 3.11) |
| I20 | I25 | Angina pectoris | Chronic ischaemic heart disease | 1046 | 0 | 28.99 (26.5, 31.72) |
| I20 | I44 | Angina pectoris | Atrioventricular and left bundle-branch block | 19 | 1.13E-06 | 3.21 (1.94, 5) |
| I20 | I50 | Angina pectoris | Heart failure | 37 | 1.32E-15 | 4.04 (2.82, 5.6) |
| I20 | I51 | Angina pectoris | Complications and ill-defined descriptions of heart disease | 22 | 9.41E-09 | 3.62 (2.27, 5.49) |
| I20 | I73 | Angina pectoris | Other peripheral vascular diseases | 23 | 3.19E-08 | 3.39 (2.15, 5.08) |
| I20 | J45 | Angina pectoris | Asthma | 136 | 6.16E-07 | 1.56 (1.31, 1.86) |
| I21 | I25 | Acute myocardial infarction | Chronic ischaemic heart disease | 524 | 0 | 10.22 (9.17, 11.37) |
| I21 | I44 | Acute myocardial infarction | Atrioventricular and left bundle-branch block | 27 | 3.88E-17 | 5.7 (3.72, 8.4) |
| I21 | I46 | Acute myocardial infarction | Cardiac arrest | 49 | 6.68E-80 | 53.11 (35.16, 80.19) |
| I21 | I47 | Acute myocardial infarction | Paroxysmal tachycardia | 37 | 2.26E-23 | 5.77 (4.02, 8.04) |
| I21 | I49 | Acute myocardial infarction | Other cardiac arrhythmias | 36 | 4.44E-30 | 8.05 (5.54, 11.37) |
| I21 | I50 | Acute myocardial infarction | Heart failure | 83 | 6.58E-87 | 12.34 (9.56, 15.75) |
| I21 | I51 | Acute myocardial infarction | Complications and ill-defined descriptions of heart disease | 34 | 4.07E-25 | 6.93 (4.73, 9.86) |
| I22 | I25 | Subsequent myocardial infarction | Chronic ischaemic heart disease | 21 | 2.59E-18 | 9.97 (5.83, 16.43) |
| I24 | I25 | Other acute ischaemic heart diseases | Chronic ischaemic heart disease | 34 | 1.30E-38 | 22.04 (13.78, 35) |
| I25 | I34 | Chronic ischaemic heart disease | Nonrheumatic mitral valve disorders | 27 | 4.95E-11 | 3.96 (2.57, 5.86) |
| I25 | I35 | Chronic ischaemic heart disease | Nonrheumatic aortic valve disorders | 46 | 6.41E-29 | 6.57 (4.67, 9.05) |
| I25 | I44 | Chronic ischaemic heart disease | Atrioventricular and left bundle-branch block | 42 | 8.54E-22 | 5.24 (3.69, 7.27) |
| I25 | I45 | Chronic ischaemic heart disease | Other conduction disorders | 22 | 1.42E-07 | 3.34 (2.07, 5.12) |
| I25 | I46 | Chronic ischaemic heart disease | Cardiac arrest | 31 | 2.36E-30 | 12.94 (8.25, 19.88) |
| I25 | I47 | Chronic ischaemic heart disease | Paroxysmal tachycardia | 43 | 4.71E-16 | 3.79 (2.71, 5.17) |
| I25 | I49 | Chronic ischaemic heart disease | Other cardiac arrhythmias | 33 | 1.89E-13 | 4.04 (2.74, 5.76) |
| I25 | I50 | Chronic ischaemic heart disease | Heart failure | 122 | 4.11E-106 | 11.71 (9.36, 14.55) |
| I25 | I51 | Chronic ischaemic heart disease | Complications and ill-defined descriptions of heart disease | 61 | 8.29E-44 | 8.02 (5.94, 10.68) |
| I25 | I73 | Chronic ischaemic heart disease | Other peripheral vascular diseases | 34 | 1.98E-11 | 3.53 (2.41, 5) |
| I26 | I80 | Pulmonary embolism | Phlebitis and thrombophlebitis | 65 | 1.05E-172 | 53.45 (40.16, 70.13) |
| I26 | J18 | Pulmonary embolism | Pneumonia, organism unspecified | 25 | 8.93E-34 | 12.69 (8.2, 18.73) |
| I34 | I48 | Nonrheumatic mitral valve disorders | Atrial fibrillation and flutter | 56 | 1.49E-92 | 25.51 (18.55, 34.57) |
| I34 | I50 | Nonrheumatic mitral valve disorders | Heart failure | 34 | 4.83E-91 | 52.77 (35.41, 76.45) |
| I34 | I51 | Nonrheumatic mitral valve disorders | Complications and ill-defined descriptions of heart disease | 25 | 1.04E-72 | 55.44 (35.07, 84.13) |
| I35 | I51 | Nonrheumatic aortic valve disorders | Complications and ill-defined descriptions of heart disease | 19 | 8.71E-47 | 35.63 (21.21, 56.52) |
| I42 | I48 | Cardiomyopathy | Atrial fibrillation and flutter | 46 | 4.75E-84 | 35.77 (24.75, 50.97) |
| I42 | I50 | Cardiomyopathy | Heart failure | 36 | 6.86E-112 | 95.35 (63.36, 140.47) |
| I44 | I48 | Atrioventricular and left bundle-branch block | Atrial fibrillation and flutter | 28 | 1.53E-25 | 8.38 (5.5, 12.26) |
| I47 | I48 | Paroxysmal tachycardia | Atrial fibrillation and flutter | 32 | 1.29E-24 | 6.78 (4.61, 9.63) |
| I48 | I49 | Atrial fibrillation and flutter | Other cardiac arrhythmias | 22 | 1.00E-16 | 6.51 (4.07, 9.89) |
| I48 | I50 | Atrial fibrillation and flutter | Heart failure | 77 | 2.21E-101 | 16.48 (12.67, 21.19) |
| I48 | I51 | Atrial fibrillation and flutter | Complications and ill-defined descriptions of heart disease | 41 | 2.84E-48 | 12.58 (8.84, 17.46) |
| I48 | I63 | Atrial fibrillation and flutter | Cerebral infarction | 21 | 3.36E-11 | 4.52 (2.8, 6.88) |
| I48 | J45 | Atrial fibrillation and flutter | Asthma | 84 | 3.13E-06 | 1.7 (1.35, 2.11) |
| I50 | I51 | Heart failure | Complications and ill-defined descriptions of heart disease | 19 | 9.26E-34 | 19.47 (11.67, 30.63) |
| I73 | I77 | Other peripheral vascular diseases | Other disorders of arteries and arterioles | 20 | 1.33E-65 | 72.35 (43.13, 115.41) |
| I84 | K57 | Haemorrhoids | Diverticular disease of intestine | 379 | 3.79E-182 | 5.2 (4.64, 5.81) |
| I84 | K58 | Haemorrhoids | Irritable bowel syndrome | 48 | 1.33E-06 | 2.05 (1.51, 2.72) |
| I84 | K59 | Haemorrhoids | Other functional intestinal disorders | 69 | 7.45E-08 | 1.95 (1.51, 2.47) |
| I84 | K60 | Haemorrhoids | Fissure and fistula of anal and rectal regions | 185 | 8.63E-167 | 10.51 (8.87, 12.39) |
| I84 | K62 | Haemorrhoids | Other diseases of anus and rectum | 822 | 0 | 7.34 (6.77, 7.95) |
| I84 | K63 | Haemorrhoids | Other diseases of intestine | 108 | 3.37E-25 | 2.86 (2.33, 3.47) |
| I84 | K92 | Haemorrhoids | Other diseases of digestive system | 94 | 3.55E-17 | 2.48 (1.99, 3.05) |
| I84 | L29 | Haemorrhoids | Pruritus | 20 | 5.70E-14 | 6.15 (3.72, 9.65) |
| J18 | J45 | Pneumonia, organism unspecified | Asthma | 84 | 1.70E-23 | 3.17 (2.51, 3.95) |
| J18 | J90 | Pneumonia, organism unspecified | Pleural effusion, not elsewhere classified | 81 | 8.05E-211 | 58.84 (45.23, 75.76) |
| J18 | J96 | Pneumonia, organism unspecified | Respiratory failure, not elsewhere classified | 26 | 5.44E-72 | 62.9 (39.32, 97.46) |
| J18 | J98 | Pneumonia, organism unspecified | Other respiratory disorders | 23 | 1.82E-40 | 19.3 (12.16, 29.18) |
| J18 | N17 | Pneumonia, organism unspecified | Acute renal failure | 23 | 7.31E-43 | 21.81 (13.69, 33.12) |
| J22 | J45 | Unspecified acute lower respiratory infection | Asthma | 148 | 5.02E-96 | 6.78 (5.64, 8.1) |
| J30 | J34 | Vasomotor and allergic rhinitis | Other disorders of nose and nasal sinuses | 31 | 1.34E-32 | 10.12 (6.78, 14.58) |
| J30 | J45 | Vasomotor and allergic rhinitis | Asthma | 25 | 1.26E-07 | 3.04 (1.96, 4.5) |
| J31 | J32 | Chronic rhinitis, nasopharyngitis and pharyngitis | Chronic sinusitis | 50 | 5.55E-154 | 81.71 (58.42, 112.38) |
| J31 | J33 | Chronic rhinitis, nasopharyngitis and pharyngitis | Nasal polyp | 30 | 7.76E-60 | 26.97 (17.83, 39.44) |
| J31 | J34 | Chronic rhinitis, nasopharyngitis and pharyngitis | Other disorders of nose and nasal sinuses | 86 | 1.00E-162 | 51.19 (38.46, 67.82) |
| J32 | J33 | Chronic sinusitis | Nasal polyp | 159 | 0 | 64.34 (52.9, 77.9) |
| J32 | J34 | Chronic sinusitis | Other disorders of nose and nasal sinuses | 189 | 0 | 31.46 (26.34, 37.43) |
| J33 | J34 | Nasal polyp | Other disorders of nose and nasal sinuses | 238 | 0 | 24.21 (20.7, 28.22) |
| J33 | J45 | Nasal polyp | Asthma | 121 | 3.75E-48 | 4.18 (3.43, 5.05) |
| J34 | J45 | Other disorders of nose and nasal sinuses | Asthma | 125 | 1.48E-08 | 1.69 (1.4, 2.03) |
| J44 | J45 | Other chronic obstructive pulmonary disease | Asthma | 39 | 4.43E-09 | 2.68 (1.9, 3.68) |
| J45 | J47 | Asthma | Bronchiectasis | 24 | 3.59E-13 | 5 (3.16, 7.57) |
| J45 | L30 | Asthma | Other dermatitis | 55 | 1.52E-35 | 6.42 (4.74, 8.54) |
| J45 | M13 | Asthma | Other arthritis | 70 | 8.58E-13 | 2.45 (1.9, 3.1) |
| J45 | M19 | Asthma | Other arthrosis | 96 | 1.08E-08 | 1.83 (1.48, 2.24) |
| J90 | J98 | Pleural effusion, not elsewhere classified | Other respiratory disorders | 39 | 2.92E-130 | 87.25 (60.05, 123.77) |
| K01 | K02 | Embedded and impacted teeth | Dental caries | 42 | 1.15E-81 | 24.76 (17.58, 33.98) |
| K01 | K04 | Embedded and impacted teeth | Diseases of pulp and periapical tissues | 38 | 1.94E-66 | 20.23 (14.15, 28.1) |
| K01 | K05 | Embedded and impacted teeth | Gingivitis and periodontal diseases | 23 | 2.53E-64 | 49.54 (30.79, 76.19) |
| K01 | K09 | Embedded and impacted teeth | Cysts of oral region, not elsewhere classified | 28 | 6.45E-98 | 108.73 (69.07, 166.04) |
| K02 | K04 | Dental caries | Diseases of pulp and periapical tissues | 24 | 4.18E-26 | 9.37 (6.02, 13.88) |
| K02 | K05 | Dental caries | Gingivitis and periodontal diseases | 22 | 2.47E-53 | 34.76 (21.53, 53.43) |
| K02 | K08 | Dental caries | Other disorders of teeth and supporting structures | 46 | 1.22E-86 | 22.94 (16.59, 30.96) |
| K04 | K08 | Diseases of pulp and periapical tissues | Other disorders of teeth and supporting structures | 36 | 4.78E-57 | 16.62 (11.56, 23.15) |
| K20 | K22 | Oesophagitis | Other diseases of oesophagus | 169 | 1.30E-161 | 10.03 (8.46, 11.82) |
| K20 | K25 | Oesophagitis | Gastric ulcer | 86 | 6.11E-94 | 11.05 (8.73, 13.81) |
| K20 | K26 | Oesophagitis | Duodenal ulcer | 62 | 1.39E-47 | 7.04 (5.35, 9.09) |
| K20 | K29 | Oesophagitis | Gastritis and duodenitis | 580 | 0 | 12.65 (11.42, 13.98) |
| K20 | K31 | Oesophagitis | Other diseases of stomach and duodenum | 53 | 8.88E-38 | 6.37 (4.75, 8.37) |
| K20 | K44 | Oesophagitis | Diaphragmatic hernia | 724 | 0 | 21.2 (19.24, 23.35) |
| K21 | K22 | Gastro-oesophageal reflux disease | Other diseases of oesophagus | 421 | 0 | 15.12 (13.45, 16.95) |
| K21 | K25 | Gastro-oesophageal reflux disease | Gastric ulcer | 66 | 4.45E-25 | 3.82 (2.93, 4.88) |
| K21 | K26 | Gastro-oesophageal reflux disease | Duodenal ulcer | 74 | 3.25E-31 | 4.19 (3.26, 5.29) |
| K21 | K29 | Gastro-oesophageal reflux disease | Gastritis and duodenitis | 886 | 0 | 8.81 (8.13, 9.53) |
| K21 | K30 | Gastro-oesophageal reflux disease | Functional dyspepsia | 137 | 3.16E-19 | 2.22 (1.85, 2.63) |
| K21 | K31 | Gastro-oesophageal reflux disease | Other diseases of stomach and duodenum | 97 | 1.80E-58 | 5.84 (4.68, 7.2) |
| K21 | K44 | Gastro-oesophageal reflux disease | Diaphragmatic hernia | 1236 | 0 | 17.42 (16.18, 18.75) |
| K22 | K25 | Other diseases of oesophagus | Gastric ulcer | 47 | 2.19E-32 | 6.15 (4.49, 8.2) |
| K22 | K26 | Other diseases of oesophagus | Duodenal ulcer | 29 | 5.87E-10 | 3.27 (2.19, 4.66) |
| K22 | K29 | Other diseases of oesophagus | Gastritis and duodenitis | 411 | 1.61E-305 | 8.88 (7.91, 9.95) |
| K22 | K31 | Other diseases of oesophagus | Other diseases of stomach and duodenum | 65 | 2.01E-61 | 8.96 (6.85, 11.52) |
| K22 | K44 | Other diseases of oesophagus | Diaphragmatic hernia | 641 | 0 | 20.64 (18.62, 22.87) |
| K22 | K92 | Other diseases of oesophagus | Other diseases of digestive system | 35 | 1.66E-06 | 2.29 (1.6, 3.17) |
| K25 | K26 | Gastric ulcer | Duodenal ulcer | 68 | 7.89E-116 | 20.98 (16.03, 27.03) |
| K25 | K29 | Gastric ulcer | Gastritis and duodenitis | 320 | 0 | 18.98 (16.42, 21.91) |
| K25 | K31 | Gastric ulcer | Other diseases of stomach and duodenum | 23 | 8.16E-18 | 6.34 (4.04, 9.42) |
| K25 | K44 | Gastric ulcer | Diaphragmatic hernia | 137 | 2.01E-85 | 6.43 (5.31, 7.71) |
| K25 | K92 | Gastric ulcer | Other diseases of digestive system | 43 | 2.67E-33 | 6.81 (4.91, 9.19) |
| K26 | K29 | Duodenal ulcer | Gastritis and duodenitis | 319 | 0 | 17.84 (15.44, 20.58) |
| K26 | K31 | Duodenal ulcer | Other diseases of stomach and duodenum | 22 | 2.14E-16 | 6.07 (3.83, 9.11) |
| K26 | K44 | Duodenal ulcer | Diaphragmatic hernia | 126 | 5.02E-70 | 5.68 (4.66, 6.86) |
| K26 | K92 | Duodenal ulcer | Other diseases of digestive system | 39 | 1.09E-24 | 5.54 (3.93, 7.57) |
| K29 | K30 | Gastritis and duodenitis | Functional dyspepsia | 226 | 1.34E-36 | 2.44 (2.11, 2.79) |
| K29 | K31 | Gastritis and duodenitis | Other diseases of stomach and duodenum | 266 | 1.08E-261 | 13.82 (11.89, 16.02) |
| K29 | K44 | Gastritis and duodenitis | Diaphragmatic hernia | 1524 | 0 | 13.6 (12.73, 14.52) |
| K29 | K90 | Gastritis and duodenitis | Intestinal malabsorption | 35 | 4.95E-08 | 2.63 (1.82, 3.66) |
| K29 | K92 | Gastritis and duodenitis | Other diseases of digestive system | 104 | 1.00E-12 | 2.08 (1.69, 2.53) |
| K30 | K31 | Functional dyspepsia | Other diseases of stomach and duodenum | 37 | 2.48E-10 | 2.92 (2.06, 4.01) |
| K30 | K44 | Functional dyspepsia | Diaphragmatic hernia | 199 | 1.64E-31 | 2.39 (2.06, 2.76) |
| K31 | K44 | Other diseases of stomach and duodenum | Diaphragmatic hernia | 201 | 1.58E-176 | 10.38 (8.81, 12.18) |
| K42 | K43 | Umbilical hernia | Ventral hernia | 19 | 1.25E-20 | 9.26 (5.6, 14.38) |
| K44 | K90 | Diaphragmatic hernia | Intestinal malabsorption | 33 | 1.76E-08 | 2.79 (1.91, 3.92) |
| K50 | K56 | Crohn disease [regional enteritis] | Paralytic ileus and intestinal obstruction without hernia | 20 | 4.94E-42 | 24.47 (14.95, 37.79) |
| K52 | K57 | Other noninfective gastroenteritis and colitis | Diverticular disease of intestine | 125 | 4.18E-24 | 2.58 (2.13, 3.08) |
| K52 | K59 | Other noninfective gastroenteritis and colitis | Other functional intestinal disorders | 195 | 1.22E-185 | 10.07 (8.59, 11.74) |
| K52 | K62 | Other noninfective gastroenteritis and colitis | Other diseases of anus and rectum | 316 | 2.19E-115 | 4.05 (3.58, 4.56) |
| K52 | K63 | Other noninfective gastroenteritis and colitis | Other diseases of intestine | 69 | 5.15E-20 | 3.14 (2.44, 3.98) |
| K52 | K92 | Other noninfective gastroenteritis and colitis | Other diseases of digestive system | 54 | 6.47E-11 | 2.5 (1.87, 3.25) |
| K56 | K57 | Paralytic ileus and intestinal obstruction without hernia | Diverticular disease of intestine | 23 | 1.78E-07 | 3.1 (1.97, 4.63) |
| K57 | K58 | Diverticular disease of intestine | Irritable bowel syndrome | 36 | 7.04E-07 | 2.34 (1.64, 3.23) |
| K57 | K59 | Diverticular disease of intestine | Other functional intestinal disorders | 61 | 9.24E-12 | 2.47 (1.88, 3.17) |
| K57 | K62 | Diverticular disease of intestine | Other diseases of anus and rectum | 410 | 2.27E-171 | 4.65 (4.17, 5.18) |
| K57 | K63 | Diverticular disease of intestine | Other diseases of intestine | 189 | 3.13E-129 | 7.03 (5.99, 8.22) |
| K57 | K64 | Diverticular disease of intestine | Haemorrhoids and perianal venous thrombosis | 58 | 1.62E-38 | 6.42 (4.81, 8.42) |
| K59 | K62 | Other functional intestinal disorders | Other diseases of anus and rectum | 138 | 4.50E-42 | 3.42 (2.85, 4.07) |
| K59 | K63 | Other functional intestinal disorders | Other diseases of intestine | 31 | 2.18E-08 | 2.8 (1.91, 3.94) |
| K59 | K92 | Other functional intestinal disorders | Other diseases of digestive system | 33 | 1.28E-10 | 3.15 (2.17, 4.39) |
| K60 | K62 | Fissure and fistula of anal and rectal regions | Other diseases of anus and rectum | 98 | 4.48E-38 | 4.07 (3.27, 5.02) |
| K62 | K63 | Other diseases of anus and rectum | Other diseases of intestine | 213 | 3.15E-105 | 5.2 (4.47, 6.01) |
| K62 | K64 | Other diseases of anus and rectum | Haemorrhoids and perianal venous thrombosis | 45 | 5.29E-15 | 3.47 (2.51, 4.68) |
| K63 | K64 | Other diseases of intestine | Haemorrhoids and perianal venous thrombosis | 38 | 8.46E-38 | 9.21 (6.46, 12.75) |
| K63 | K92 | Other diseases of intestine | Other diseases of digestive system | 36 | 1.84E-09 | 2.8 (1.97, 3.86) |
| K64 | K92 | Haemorrhoids and perianal venous thrombosis | Other diseases of digestive system | 32 | 5.69E-31 | 8.74 (5.94, 12.39) |
| K66 | K80 | Other disorders of peritoneum | Cholelithiasis | 51 | 1.57E-56 | 11.78 (8.59, 15.85) |
| K66 | N83 | Other disorders of peritoneum | Noninflammatory disorders of ovary, fallopian tube and broad ligament | 30 | 4.23E-27 | 8.77 (5.82, 12.78) |
| K76 | K80 | Other diseases of liver | Cholelithiasis | 29 | 1.30E-22 | 6.89 (4.58, 9.95) |
| K80 | K82 | Cholelithiasis | Other diseases of gallbladder | 136 | 6.17E-238 | 74.56 (57.72, 96.49) |
| K80 | K83 | Cholelithiasis | Other diseases of biliary tract | 58 | 4.52E-100 | 32.01 (23.1, 43.87) |
| K80 | K85 | Cholelithiasis | Acute pancreatitis | 148 | 9.93E-274 | 49.19 (39.59, 60.98) |
| K81 | K82 | Cholecystitis | Other diseases of gallbladder | 27 | 3.59E-78 | 52.14 (33.75, 77.5) |
| L02 | L03 | Cutaneous abscess, furuncle and carbuncle | Cellulitis | 41 | 2.42E-38 | 8.48 (6.04, 11.56) |
| L03 | L30 | Cellulitis | Other dermatitis | 19 | 1.83E-18 | 8.16 (4.93, 12.69) |
| L03 | M70 | Cellulitis | Soft tissue disorders related to use, overuse and pressure | 42 | 4.33E-61 | 16.23 (11.5, 22.34) |
| L08 | L72 | Other local infections of skin and subcutaneous tissue | Follicular cysts of skin and subcutaneous tissue | 25 | 1.39E-24 | 8.96 (5.75, 13.37) |
| L40 | M07 | Psoriasis | Psoriatic and enteropathic arthropathies | 149 | 1.39E-282 | 2923.17 (1925.01, 4612.08) |
| L84 | M20 | Corns and callosities | Acquired deformities of fingers and toes | 34 | 2.64E-59 | 69.99 (41.98, 117.27) |
| M06 | M65 | Other rheumatoid arthritis | Synovitis and tenosynovitis | 24 | 1.69E-13 | 4.72 (3.04, 6.96) |
| M11 | M17 | Other crystal arthropathies | Gonarthrosis [arthrosis of knee] | 31 | 5.47E-49 | 34.84 (21.58, 55.69) |
| M11 | M23 | Other crystal arthropathies | Internal derangement of knee | 38 | 1.83E-51 | 38.5 (24.03, 61.95) |
| M17 | M21 | Gonarthrosis [arthrosis of knee] | Other acquired deformities of limbs | 30 | 7.12E-27 | 8.41 (5.59, 12.19) |
| M17 | M22 | Gonarthrosis [arthrosis of knee] | Disorders of patella | 56 | 4.67E-82 | 20.28 (14.8, 27.39) |
| M17 | M23 | Gonarthrosis [arthrosis of knee] | Internal derangement of knee | 1275 | 0 | 38.67 (35.62, 41.97) |
| M17 | M24 | Gonarthrosis [arthrosis of knee] | Other specific joint derangements | 44 | 2.27E-25 | 5.3 (3.82, 7.16) |
| M17 | M25 | Gonarthrosis [arthrosis of knee] | Other joint disorders, not elsewhere classified | 114 | 3.07E-25 | 2.77 (2.27, 3.34) |
| M17 | M65 | Gonarthrosis [arthrosis of knee] | Synovitis and tenosynovitis | 105 | 6.31E-67 | 6.15 (4.98, 7.52) |
| M17 | M67 | Gonarthrosis [arthrosis of knee] | Other disorders of synovium and tendon | 58 | 9.85E-21 | 3.6 (2.72, 4.66) |
| M19 | M20 | Other arthrosis | Acquired deformities of fingers and toes | 138 | 1.10E-85 | 6.3 (5.22, 7.53) |
| M19 | M24 | Other arthrosis | Other specific joint derangements | 37 | 1.16E-38 | 9.66 (6.76, 13.4) |
| M19 | M25 | Other arthrosis | Other joint disorders, not elsewhere classified | 96 | 4.08E-49 | 4.89 (3.94, 6.01) |
| M19 | M47 | Other arthrosis | Spondylosis | 23 | 2.35E-06 | 2.74 (1.75, 4.06) |
| M19 | M65 | Other arthrosis | Synovitis and tenosynovitis | 29 | 1.70E-08 | 2.93 (1.97, 4.18) |
| M19 | M75 | Other arthrosis | Shoulder lesions | 123 | 4.62E-103 | 8.19 (6.73, 9.87) |
| M20 | M21 | Acquired deformities of fingers and toes | Other acquired deformities of limbs | 29 | 1.42E-31 | 11.36 (7.44, 16.74) |
| M20 | M24 | Acquired deformities of fingers and toes | Other specific joint derangements | 20 | 2.46E-08 | 3.74 (2.3, 5.73) |
| M20 | M25 | Acquired deformities of fingers and toes | Other joint disorders, not elsewhere classified | 86 | 1.63E-22 | 3.04 (2.42, 3.77) |
| M20 | M77 | Acquired deformities of fingers and toes | Other enthesopathies | 39 | 2.47E-29 | 6.94 (4.88, 9.58) |
| M20 | M89 | Acquired deformities of fingers and toes | Other disorders of bone | 20 | 9.83E-13 | 5.49 (3.35, 8.49) |
| M22 | M23 | Disorders of patella | Internal derangement of knee | 95 | 3.98E-131 | 28.78 (21.91, 37.65) |
| M23 | M24 | Internal derangement of knee | Other specific joint derangements | 80 | 1.48E-53 | 6.81 (5.3, 8.64) |
| M23 | M25 | Internal derangement of knee | Other joint disorders, not elsewhere classified | 109 | 6.36E-09 | 1.78 (1.46, 2.16) |
| M23 | M65 | Internal derangement of knee | Synovitis and tenosynovitis | 128 | 2.16E-75 | 5.93 (4.88, 7.14) |
| M23 | M67 | Internal derangement of knee | Other disorders of synovium and tendon | 70 | 6.89E-20 | 3.16 (2.45, 4.01) |
| M23 | M93 | Internal derangement of knee | Other osteochondropathies | 23 | 1.41E-26 | 14.91 (8.91, 24.15) |
| M23 | M94 | Internal derangement of knee | Other disorders of cartilage | 55 | 1.05E-74 | 24.07 (17.02, 33.7) |
| M24 | M25 | Other specific joint derangements | Other joint disorders, not elsewhere classified | 45 | 8.76E-34 | 6.77 (4.9, 9.11) |
| M24 | M65 | Other specific joint derangements | Synovitis and tenosynovitis | 27 | 1.45E-30 | 10.05 (6.63, 14.6) |
| M24 | M75 | Other specific joint derangements | Shoulder lesions | 24 | 1.67E-14 | 5.03 (3.24, 7.43) |
| M25 | M65 | Other joint disorders, not elsewhere classified | Synovitis and tenosynovitis | 51 | 3.09E-20 | 3.81 (2.83, 5.01) |
| M43 | M47 | Other deforming dorsopathies | Spondylosis | 28 | 9.58E-70 | 40.93 (26.59, 60.79) |
| M43 | M48 | Other deforming dorsopathies | Other spondylopathies | 30 | 3.81E-109 | 102.13 (66.74, 151.46) |
| M43 | M51 | Other deforming dorsopathies | Other intervertebral disc disorders | 43 | 9.26E-94 | 39.51 (27.54, 55.6) |
| M47 | M48 | Spondylosis | Other spondylopathies | 57 | 2.47E-130 | 36.95 (27.37, 49.03) |
| M47 | M50 | Spondylosis | Cervical disc disorders | 27 | 4.76E-58 | 28.38 (18.47, 41.9) |
| M47 | M51 | Spondylosis | Other intervertebral disc disorders | 109 | 4.77E-173 | 19.98 (16.13, 24.53) |
| M47 | M54 | Spondylosis | Dorsalgia | 39 | 2.04E-11 | 3.02 (2.15, 4.12) |
| M48 | M51 | Other spondylopathies | Other intervertebral disc disorders | 117 | 1.64E-273 | 59.07 (46.98, 73.86) |
| M51 | M54 | Other intervertebral disc disorders | Dorsalgia | 76 | 3.05E-21 | 3.09 (2.42, 3.88) |
| M54 | M79 | Dorsalgia | Other soft tissue disorders, not elsewhere classified | 125 | 2.38E-47 | 3.9 (3.22, 4.67) |
| M65 | M75 | Synovitis and tenosynovitis | Shoulder lesions | 34 | 1.86E-11 | 3.26 (2.26, 4.53) |
| M75 | M77 | Shoulder lesions | Other enthesopathies | 21 | 4.01E-12 | 4.75 (2.96, 7.18) |
| M88 | N92 | Paget disease of bone [osteitis deformans] | Excessive, frequent and irregular menstruation | 25 | 2.97E-07 | 3.55 (2.17, 5.62) |
| N12 | N39 | Tubulo-interstitial nephritis, not specified as acute or chronic | Other disorders of urinary system | 23 | 4.61E-17 | 6.8 (4.24, 10.43) |
| N13 | N20 | Obstructive and reflux uropathyC | Calculus of kidney and ureter | 30 | 2.85E-34 | 10.8 (7.22, 15.55) |
| N13 | N32 | Obstructive and reflux uropathyC | Other disorders of bladder | 20 | 2.32E-15 | 6.33 (3.88, 9.73) |
| N20 | N23 | Calculus of kidney and ureter | Unspecified renal colic | 99 | 1.98E-141 | 18.03 (14.34, 22.45) |
| N20 | N28 | Calculus of kidney and ureter | Other disorders of kidney and ureter, not elsewhere classified | 26 | 3.28E-33 | 12.56 (8.12, 18.62) |
| N28 | N39 | Other disorders of kidney and ureter, not elsewhere classified | Other disorders of urinary system | 21 | 7.11E-11 | 4.47 (2.76, 6.85) |
| N30 | N32 | Cystitis | Other disorders of bladder | 42 | 4.98E-69 | 19.24 (13.65, 26.44) |
| N30 | N35 | Cystitis | Urethral stricture | 36 | 1.39E-86 | 36.05 (24.85, 50.79) |
| N31 | N32 | Neuromuscular dysfunction of bladder, not elsewhere classified | Other disorders of bladder | 31 | 6.82E-47 | 16.66 (11.15, 24.05) |
| N31 | N39 | Neuromuscular dysfunction of bladder, not elsewhere classified | Other disorders of urinary system | 33 | 1.02E-22 | 6.35 (4.31, 9.06) |
| N32 | N35 | Other disorders of bladder | Urethral stricture | 64 | 4.13E-82 | 13.74 (10.42, 17.81) |
| N32 | N39 | Other disorders of bladder | Other disorders of urinary system | 125 | 1.39E-75 | 5.93 (4.88, 7.14) |
| N32 | N40 | Other disorders of bladder | Hyperplasia of prostate | 199 | 4.96E-232 | 16.95 (14.27, 20.05) |
| N32 | N81 | Other disorders of bladder | Female genital prolapse | 49 | 8.23E-20 | 4.1 (3, 5.47) |
| N35 | N39 | Urethral stricture | Other disorders of urinary system | 34 | 7.84E-11 | 3.19 (2.21, 4.46) |
| N35 | N40 | Urethral stricture | Hyperplasia of prostate | 24 | 7.59E-13 | 4.71 (3.01, 7.03) |
| N39 | N40 | Other disorders of urinary system | Hyperplasia of prostate | 51 | 9.32E-20 | 3.89 (2.87, 5.14) |
| N39 | N81 | Other disorders of urinary system | Female genital prolapse | 442 | 2.52E-244 | 6.36 (5.7, 7.08) |
| N39 | N99 | Other disorders of urinary system | Postprocedural disorders of genitourinary system, not elsewhere classified | 21 | 5.24E-16 | 7.01 (4.26, 11) |
| N40 | N41 | Hyperplasia of prostate | Inflammatory diseases of prostate | 29 | 1.67E-24 | 8.05 (5.3, 11.81) |
| N43 | N50 | Hydrocele and spermatocele | Other disorders of male genital organs | 31 | 3.42E-29 | 8.5 (5.74, 12.16) |
| N47 | N48 | Redundant prepuce, phimosis and paraphimosis | Other disorders of penis | 86 | 1.24E-156 | 29.03 (22.54, 37.01) |
| N60 | N62 | Benign mammary dysplasia | Hypertrophy of breast | 32 | 6.29E-55 | 20.7 (13.91, 29.82) |
| N60 | N64 | Benign mammary dysplasia | Other disorders of breast | 24 | 1.92E-28 | 10.85 (6.93, 16.19) |
| N70 | N73 | Salpingitis and oophoritis | Other female pelvic inflammatory diseases | 30 | 3.57E-57 | 29.39 (19.1, 43.89) |
| N70 | N80 | Salpingitis and oophoritis | Endometriosis | 31 | 4.84E-38 | 14.76 (9.66, 21.92) |
| N70 | N83 | Salpingitis and oophoritis | Noninflammatory disorders of ovary, fallopian tube and broad ligament | 33 | 1.89E-37 | 12.83 (8.54, 18.73) |
| N72 | N73 | Inflammatory disease of cervix uteri | Other female pelvic inflammatory diseases | 19 | 3.18E-13 | 5.79 (3.5, 9.01) |
| N72 | N80 | Inflammatory disease of cervix uteri | Endometriosis | 52 | 2.35E-45 | 8.86 (6.49, 11.86) |
| N72 | N81 | Inflammatory disease of cervix uteri | Female genital prolapse | 50 | 1.97E-22 | 4.38 (3.21, 5.84) |
| N72 | N83 | Inflammatory disease of cervix uteri | Noninflammatory disorders of ovary, fallopian tube and broad ligament | 52 | 1.83E-37 | 6.91 (5.09, 9.2) |
| N72 | N84 | Inflammatory disease of cervix uteri | Polyp of female genital tract | 61 | 3.80E-18 | 3.34 (2.52, 4.34) |
| N72 | N85 | Inflammatory disease of cervix uteri | Other noninflammatory disorders of uterus, except cervix | 29 | 6.31E-12 | 3.79 (2.54, 5.43) |
| N72 | N87 | Inflammatory disease of cervix uteri | Dysplasia of cervix uteri | 48 | 1.29E-68 | 16.18 (11.71, 21.86) |
| N72 | N88 | Inflammatory disease of cervix uteri | Other noninflammatory disorders of cervix uteri | 32 | 2.10E-39 | 11.82 (8.02, 16.82) |
| N73 | N80 | Other female pelvic inflammatory diseases | Endometriosis | 146 | 5.45E-183 | 19.28 (15.73, 23.51) |
| N73 | N83 | Other female pelvic inflammatory diseases | Noninflammatory disorders of ovary, fallopian tube and broad ligament | 149 | 2.96E-171 | 15.98 (13.12, 19.36) |
| N73 | N85 | Other female pelvic inflammatory diseases | Other noninflammatory disorders of uterus, except cervix | 39 | 5.27E-15 | 3.75 (2.66, 5.14) |
| N73 | N94 | Other female pelvic inflammatory diseases | Pain and other conditions associated with female genital organs and menstrual cycle | 51 | 5.21E-24 | 4.67 (3.42, 6.23) |
| N80 | N81 | Endometriosis | Female genital prolapse | 107 | 1.74E-37 | 3.82 (3.1, 4.67) |
| N80 | N83 | Endometriosis | Noninflammatory disorders of ovary, fallopian tube and broad ligament | 249 | 5.65E-247 | 13.63 (11.69, 15.85) |
| N80 | N84 | Endometriosis | Polyp of female genital tract | 114 | 3.32E-16 | 2.3 (1.88, 2.78) |
| N80 | N85 | Endometriosis | Other noninflammatory disorders of uterus, except cervix | 64 | 7.75E-18 | 3.15 (2.41, 4.04) |
| N80 | N88 | Endometriosis | Other noninflammatory disorders of cervix uteri | 31 | 2.32E-13 | 3.98 (2.69, 5.66) |
| N80 | N94 | Endometriosis | Pain and other conditions associated with female genital organs and menstrual cycle | 65 | 1.79E-14 | 2.78 (2.12, 3.58) |
| N80 | N97 | Endometriosis | Female infertility | 28 | 7.58E-15 | 5.53 (3.58, 8.24) |
| N81 | N84 | Female genital prolapse | Polyp of female genital tract | 232 | 3.21E-15 | 1.74 (1.51, 1.99) |
| N81 | N85 | Female genital prolapse | Other noninflammatory disorders of uterus, except cervix | 171 | 3.47E-52 | 3.52 (2.98, 4.13) |
| N81 | N88 | Female genital prolapse | Other noninflammatory disorders of cervix uteri | 48 | 5.45E-10 | 2.55 (1.87, 3.4) |
| N83 | N85 | Noninflammatory disorders of ovary, fallopian tube and broad ligament | Other noninflammatory disorders of uterus, except cervix | 69 | 5.77E-13 | 2.49 (1.92, 3.16) |
| N83 | N88 | Noninflammatory disorders of ovary, fallopian tube and broad ligament | Other noninflammatory disorders of cervix uteri | 29 | 1.37E-07 | 2.75 (1.84, 3.94) |
| N83 | N94 | Noninflammatory disorders of ovary, fallopian tube and broad ligament | Pain and other conditions associated with female genital organs and menstrual cycle | 55 | 9.05E-07 | 2.03 (1.52, 2.65) |
| N84 | N85 | Polyp of female genital tract | Other noninflammatory disorders of uterus, except cervix | 329 | 1.95E-142 | 4.95 (4.37, 5.59) |
| N84 | N86 | Polyp of female genital tract | Erosion and ectropion of cervix uteri | 81 | 1.08E-41 | 5.55 (4.31, 7.06) |
| N84 | N88 | Polyp of female genital tract | Other noninflammatory disorders of cervix uteri | 101 | 9.64E-35 | 3.82 (3.07, 4.71) |
| N84 | N92 | Polyp of female genital tract | Excessive, frequent and irregular menstruation | 817 | 1.23E-181 | 3.47 (3.19, 3.77) |
| N84 | N93 | Polyp of female genital tract | Other abnormal uterine and vaginal bleeding | 217 | 6.38E-52 | 3.1 (2.67, 3.58) |
| N84 | N95 | Polyp of female genital tract | Menopausal and other perimenopausal disorders | 807 | 0 | 5.6 (5.15, 6.1) |
| N85 | N86 | Other noninflammatory disorders of uterus, except cervix | Erosion and ectropion of cervix uteri | 46 | 6.52E-35 | 7.22 (5.22, 9.76) |
| N85 | N88 | Other noninflammatory disorders of uterus, except cervix | Other noninflammatory disorders of cervix uteri | 105 | 2.15E-106 | 10.93 (8.78, 13.47) |
| N85 | N89 | Other noninflammatory disorders of uterus, except cervix | Other noninflammatory disorders of vagina | 31 | 1.86E-15 | 4.47 (3.03, 6.35) |
| N85 | N92 | Other noninflammatory disorders of uterus, except cervix | Excessive, frequent and irregular menstruation | 409 | 2.43E-129 | 4.46 (3.95, 5.02) |
| N85 | N93 | Other noninflammatory disorders of uterus, except cervix | Other abnormal uterine and vaginal bleeding | 89 | 9.35E-23 | 3.01 (2.4, 3.73) |
| N85 | N94 | Other noninflammatory disorders of uterus, except cervix | Pain and other conditions associated with female genital organs and menstrual cycle | 48 | 1.82E-08 | 2.38 (1.74, 3.16) |
| N85 | N95 | Other noninflammatory disorders of uterus, except cervix | Menopausal and other perimenopausal disorders | 451 | 6.01E-295 | 8.78 (7.81, 9.84) |
| N86 | N88 | Erosion and ectropion of cervix uteri | Other noninflammatory disorders of cervix uteri | 22 | 8.00E-22 | 8.64 (5.4, 13.1) |
| N86 | N92 | Erosion and ectropion of cervix uteri | Excessive, frequent and irregular menstruation | 126 | 8.35E-31 | 3.71 (2.97, 4.6) |
| N86 | N93 | Erosion and ectropion of cervix uteri | Other abnormal uterine and vaginal bleeding | 44 | 5.54E-23 | 5.05 (3.62, 6.87) |
| N86 | N95 | Erosion and ectropion of cervix uteri | Menopausal and other perimenopausal disorders | 35 | 1.50E-11 | 3.55 (2.44, 5.01) |
| N87 | N88 | Dysplasia of cervix uteri | Other noninflammatory disorders of cervix uteri | 21 | 2.89E-12 | 4.83 (3.01, 7.33) |
| N88 | N89 | Other noninflammatory disorders of cervix uteri | Other noninflammatory disorders of vagina | 24 | 9.92E-26 | 9.44 (6.04, 14.05) |
| N88 | N92 | Other noninflammatory disorders of cervix uteri | Excessive, frequent and irregular menstruation | 104 | 6.45E-16 | 2.48 (1.98, 3.07) |
| N88 | N93 | Other noninflammatory disorders of cervix uteri | Other abnormal uterine and vaginal bleeding | 44 | 3.07E-18 | 3.98 (2.88, 5.37) |
| N88 | N94 | Other noninflammatory disorders of cervix uteri | Pain and other conditions associated with female genital organs and menstrual cycle | 22 | 2.99E-06 | 2.81 (1.77, 4.24) |
| N88 | N95 | Other noninflammatory disorders of cervix uteri | Menopausal and other perimenopausal disorders | 148 | 5.93E-89 | 7.1 (5.85, 8.58) |
| N89 | N94 | Other noninflammatory disorders of vagina | Pain and other conditions associated with female genital organs and menstrual cycle | 35 | 1.88E-24 | 6.6 (4.53, 9.34) |
| N89 | N95 | Other noninflammatory disorders of vagina | Menopausal and other perimenopausal disorders | 36 | 8.07E-07 | 2.42 (1.68, 3.38) |
| N90 | N95 | Other noninflammatory disorders of vulva and perineum | Menopausal and other perimenopausal disorders | 69 | 2.10E-21 | 3.54 (2.71, 4.57) |
| N92 | N94 | Excessive, frequent and irregular menstruation | Pain and other conditions associated with female genital organs and menstrual cycle | 559 | 7.55E-233 | 7.57 (6.71, 8.54) |

**Table S7. The most common comorbidity patterns in the overall, male and female inpatients among Chinese and UK inpatients (27 patterns).**

|  |  |  | Overall | | | | Male | | | | Female | | | |
| --- | --- | --- | --- | --- | --- | --- | --- | --- | --- | --- | --- | --- | --- | --- |
| Patterns | Disease1 | Disease2 | Fre  (China) | Fre (UK) | OR  (China) | OR  (UK) | Fre  (China) | Fre  (UK) | OR  (China) | OR  (UK) | Fre  (China) | Fre  (UK) | OR  (China) | OR  (UK) |
| I10, I63 | Essential hypertension | Cerebral infarction | 6595  (R1) |  | 5.16  (4.97,5.37) |  | 4598  (R1) |  | 5.15  (4.91, 5.40) |  | 1997  (R3) |  | 5.12  (4.78,5.48) |  |
| E78, I10 | Dyslipidemia | Essential hypertension | 6445  (R2)* | 1312  (R2)* | 3.49  (3.37,3.62) | 13.11  (12.11,14.19) | 4375  (R2)* | 900  (R1)* | 3.49  (3.34, 3.64) | 11.89  (10.80,13.09) | 2070  (R2) |  | 3.37  (3.19, 3.60) |  |
| I25, I50 | Chronic ischaemic heart disease | Heart failure | 6174  (R3) |  | 61.33 (57.96,64.92) |  | 3953  (R3) |  | 55.99  (52.20, 60.09) |  | 2221  (R1) |  | 71.16  (64.70, 78.34) |  |
| I10, I25 | Essential hypertension | Chronic ischaemic heart disease | 5753  (R4)* | 909  (R6)* | 2.80  (2.70,2.90) | 3.81  (3.52,4.13) | 3868  (R5)* | 699  (R5)* | 2.87  (2.74, 3.00) | 3.32  (3.03,3.63) | 1885  (R4) |  | 2.63  (2.47,2.79) |  |
| E11, I10 | Type 2 diabetes mellitus | Essential hypertension | 5454  (R5)* | 818  (R9)* | 2.08  (2.00,2.15) | 8.30  (7.58,9.09) | 3907  (R4)* | 528  (R7)* | 1.92  (1.84, 2.01) | 6.99  (6.24,7.82) | 1547  (R5) |  | 2.48  (2.32,2.65) |  |
| I10, K76 | Essential hypertension | Other diseases of liver | 4377  (R6) |  | 2.10  (2.02,2 .18) |  | 3133  (R6) |  | 2.06  (1.97, 2.16) |  | 1244  (R8) |  | 2.12  (1.97,2.27) |  |
| E11, E78 | Type 2 diabetes mellitus | Dyslipidemia | 3841  (R7) |  | 4.03  (3.86,4.20) |  | 2802  (R7) |  | 3.92  (3.73, 4.12) |  |  |  |  |  |
| I10, I50 | Essential hypertension | Heart failure | 3828  (R8) |  | 3.61  (3.45,3.79) |  | 2595  (R9) |  | 3.62  (3.42, 3.84) |  | 1233  (R9) |  | 3.54  (3.27,3.83) |  |
| E78, K76 | Dyslipidemia | Other diseases of liver | 3818  (R9) |  | 5.38  (5.16,5.61) |  | 2744  (R8) |  | 5.35  (5.08, 5.63) |  | 1074  (R10) |  | 5.20  (4.82,5.61) |  |
| E11, K76 | Type 2 diabetes mellitus | Other diseases of liver | 3361  (R10) |  | 3.81  (3.65,3.98) |  | 2525  (R10) |  | 3.63  (3.45, 3.82) |  |  |  |  |  |
| K29, K44 | Gastritis and duodenitis | Diaphragmatic hernia |  | 1524  (R1) |  | 13.60  (12.73,14.53) |  | 798  (R4) |  | 13.63  (12.42,14.95) |  | 726  (R3) |  | 13.42  (12.21,14.74) |
| M17, M23 | Gonarthrosis [arthrosis of knee] | Internal derangement of knee |  | 1275  (R3) |  | 38.67  (35.63,41.97) |  | 806  (R3) |  | 32.54 (29.30,36.15) |  | 469  (R7) |  | 48.99 (43.05,55.71) |
| K21, K44 | Gastroesophageal reflux disease | Diaphragmatic hernia |  | 1236  (R4) |  | 17.43  (16.19,18.75) |  | 631  (R6) |  | 17.36 (15.63,19.25) |  | 605  (R4) |  | 17.26  (15.55,19.15) |
| I20, I25 | Angina pectoris | Chronic ischaemic heart disease |  | 1046  (R5) |  | 29.00  (26.51,31.72) |  | 856  (R2) |  | 25.71  (23.25,24.83) |  |  |  |  |
| K21, K29 | Gastro-oesophageal reflux disease | Gastritis and duodenitis |  | 886  (R7) |  | 8.81  (8.14,9.54) |  | 518  (R8) |  | 9.89  (8.88, 11.00) |  |  |  |  |
| I84, K62 | Haemorrhoids | Other diseases of anus and rectum |  | 822  (R8) |  | 7.34  (6.77,7.96) |  |  |  |  |  |  |  |  |
| N84, N92 | Polyp of female genital tract | Excessive, frequent and irregular menstruation |  | 817  (R10) |  | 3.48  (3.19,3.78) |  |  |  |  |  | 817  (R1) |  | 3.48 (3.19,3.78) |
| E78, I25 | Dyslipidemia | Chronic ischaemic heart disease |  |  |  |  |  | 491  (R9) |  | 9.66  (8.63, 10.79) |  |  |  |  |
| I10, I20 | Essential hypertension | Angina pectoris |  |  |  |  |  | 454  (R10) |  | 3.48  (3.12,3.88) |  |  |  |  |
| D25, N72 | Leiomyoma of uterus | Inflammatory disease of cervix uteri |  |  |  |  |  |  |  |  | 1394  (R6) |  | 12.28  (11.38,13.26) |  |
| G99, M47 | Other disorders of nervous system in diseases classified elsewhere | Spondylosis |  |  |  |  |  |  |  |  | 1280  (R7) |  | 471.54 (363.70,624.67) |  |
| N84, N95 | Polyp of female genital tract | Menopausal and other perimenopausal disorders |  |  |  |  |  |  |  |  |  | 807  (R2) |  | 5.61 (5.15,6.10) |
| N92, N94 | Excessive, frequent and irregular menstruation | Pain and other conditions associated with female genital organs and menstrual cycle |  |  |  |  |  |  |  |  |  | 559  (R5) |  | 7.58  (6.72,8.54) |
| I10, J45 | Essential hypertension | Asthma |  |  |  |  |  |  |  |  |  | 473  (R6) |  | 3.19  (2.88,3.53) |
| N85, N95 | Other noninflammatory disorders of uterus, except cervix | Menopausal and other perimenopausal disorders |  |  |  |  |  |  |  |  |  | 451  (R8) |  | 8.78  (7.82,9.84) |
| D25, N92 | Leiomyoma of uterus | Excessive, frequent and irregular menstruation |  |  |  |  |  |  |  |  |  | 443  (R9) |  | 3.38 (3.01,3.78) |
| N39, N81 | Other disorders of urinary system | Female genital prolapse |  |  |  |  |  |  |  |  |  | 442  (R10) |  | 6.38 (5.72,7.10) |

#Fre: Frequency; OR: odds ratio;

R+Number: this means the rank of the diseases according to degree in the corresponding complete multimorbidity networks.

*: this disease is overlapped hub diseases between China and UK in the certain group of people.

**Table S8. The hub diseases in the overall, male and female inpatients among Chinese and UK inpatients (24 diseases).**

|  |  |  | Overall | | Male | | Female | |
| --- | --- | --- | --- | --- | --- | --- | --- | --- |
| ICD-10 | Diseases | Chapter | Degree  (China) | Degree  (UK) | Degree  (China) | Degree  (UK) | Degree  (China) | Degree  (UK) |
| K76 | Other diseases of liver | C11 | 75(R1) |  | 59(R1) |  | 57(R1) |  |
| E78 | Dyslipidemia | C4 | 66(R2)* | 22(R2)* | 54(R2)* | 23(R2)* | 49(R2)* | 12(R9)* |
| E87 | Other disorders of fluid, electrolyte and acid-base balance | C4 | 62(R3) |  | 52(R3) |  | 37(R5) |  |
| I10 | Essential hypertension | C9 | 59(R4)* | 45(R1)* | 51(R4)* | 36(R1)* | 43(R3)* | 32(R1)* |
| K29 | Gastritis and duodenitis | C11 | 48(R5)* | 14(R6)* | 39(R7)* | 14(R7)* | 36(R6)* | 13(R7)* |
| I70 | Atherosclerosis | C9 | 46(R6) |  | 41(R5) |  | 32(R7) |  |
| I50 | Heart failure | C9 | 44(R7) |  | 40(R6)* | 21(R3)* | 37(R4) |  |
| E11 | Type 2 diabetes mellitus | C4 | 42(R8)* | 13(R10)* | 37(R9)* | 15(R6)* | 31(R8) |  |
| E72 | Other disorders of amino-acid metabolism | C4 | 36(R9) |  | 34(R10) |  |  |  |
| D64 | Other anemias | C3 | 36(R10) |  |  |  |  |  |
| I25 | Chronic ischemic heart disease | C9 |  | 20(R3) |  | 18(R4) |  |  |
| J45 | Asthma | C10 |  | 19(R4) |  |  |  | 16(R3) |
| D25 | Leiomyoma of uterus | C2 |  | 15(R5) |  |  | 28(R9)* | 17(R2)* |
| N85 | Other noninflammatory disorders of cervix uteri | C14 |  | 14(R7) |  |  |  | 14(R6) |
| N88 | Other noninflammatory disorders of uterus, except cervix | C14 |  | 14(R8) |  |  |  | 15(R4) |
| K44 | Diaphragmatic hernia | C11 |  | 13 (R9) |  |  |  |  |
| N40 | Hyperplasia of prostate | C14 |  |  | 38(R8) |  |  |  |
| I21 | Acute myocardial infarction | C9 |  |  |  | 16(R5) |  |  |
| I48 | Atrial fibrillation and flutter | C9 |  |  |  | 13(R8) |  |  |
| E66 | Obesity | C4 |  |  |  | 13(R9) |  | 13(R8) |
| I51 | Complications and ill-defined descriptions of heart disease | C9 |  |  |  | 13(R10) |  |  |
| N80 | Endometriosis | C14 |  |  |  |  |  | 14(R5) |
| N84 | Polyp of female genital tract | C14 |  |  |  |  |  | 11(R10) |
| I63 | Cerebral infarction | C9 |  |  |  |  | 28(R10) |  |

R + Number: this means the rank of the diseases according to degree in the corresponding complete multimorbidity networks.

*: this disease is overlapped hub diseases between China and UK in the certain group of people.

**Table S9.** **The most common diseases (top10) in the overall, male and female inpatients among Chinese and UK inpatients (24 diseases).**

|  |  |  | Overall | | Male | | Female | |
| --- | --- | --- | --- | --- | --- | --- | --- | --- |
| ICD-10 | Diseases | Chapter | Proportion%  (China) | Proportion % (UK) | Proportion % (China) | Proportion % (UK) | Proportion % (China) | Proportion % (UK) |
| I10 | Essential (primary) hypertension | C9 | 18.85 (R1) | 6.30 (R1) | 21.50 (R1) | 7.90(R1) | 15.46(R1) | 5.03(R2) |
| E11 | Type 2 diabetes mellitus | C4 | 8.75 (R2) |  | 11.15 (R2) |  | 5.69(R9) |  |
| E78 | Disorders of lipoprotein metabolism and other lipidaemias | C4 | 8.21 (R3) |  | 9.44 (R3) |  | 6.64(R3) |  |
| I25 | Chronic ischaemic heart disease | C9 | 7.76 (R4) |  | 8.65 (R5) | 3.87(R4) | 6.62(R4) |  |
| K76 | Other diseases of liver | C11 | 7.25 (R5) |  | 8.82 (R4) |  | 5.24(R10) |  |
| K29 | Gastritis and duodenitis | C11 | 7.12 (R6) | 3.46 (R3) | 6.96 (R7) | 3.98(R3) | 7.32(R2) | 3.05(R6) |
| I63 | Cerebral infarction | C9 | 6.56 (R7) |  | 7.87(R6) |  |  |  |
| D25 | Leiomyoma of uterus | C2 | 6.21 (R8) |  |  |  | 6.21(R5) |  |
| M51 | Other intervertebral disc disorders | C13 | 5.30 (R9) |  | 4.96 (R9) |  | 5.73(R8) |  |
| K80 | Cholelithiasis | C11 | 5.19 (R10) |  | 4.58 (R10) |  | 5.97(R7) |  |
| N92 | Excessive, frequent and irregular menstruation | C14 |  | 4.06 (R2) |  |  |  | 7.26(R1) |
| J45 | Asthma | C10 |  | 3.34(R4) |  | 3.18(R8) |  | 3.47(R5) |
| K62 | Other diseases of anus and rectum | C11 |  | 3.12(R5) |  | 3.60(R6) |  | 2.73(R10) |
| K44 | Diaphragmatic hernia | C11 |  | 3.06(R6) |  | 3.32(R7) |  | 2.85(R9) |
| I84 | Haemorrhoids | C9 |  | 2.62(R7) |  | 3.18(R9) |  |  |
| N84 | Polyp of female genital tract | C14 |  | 2.55(R8) |  |  |  | 4.57(R3) |
| M23 | Internal derangement of knee | C13 |  | 2.42(R9) |  | 3.61(R5) |  |  |
| N95 | Menopausal and other perimenopausal disorders | C14 |  | 2.42(R10) |  |  |  | 4.33(R4) |
| I50 | Heart failure | C9 |  |  | 5.03 (R8) |  |  |  |
| K40 | Inguinal hernia | C11 |  |  |  | 4.85(R2) |  |  |
| K21 | Gastro-oesophageal reflux disease | C11 |  |  |  | 2.51(R10) |  |  |
| M47 | Spondylosis | C13 |  |  |  |  | 6.17(R6) |  |
| N81 | Female genital prolapse | C14 |  |  |  |  |  | 3.02(R7) |
| N39 | Other disorders of urinary system | C14 |  |  |  |  |  | 3.01(R8) |

R+Number: this means the rank of the diseases according to degree in the corresponding complete multimorbidity networks.
