# Supplementary material for: Exploring multimorbidity profiles in middle-aged inpatients: a network-based comparative study of China and the United Kingdom
Source: BMC Med. 2023 Dec 13;21:495. doi: 10.1186/s12916-023-03204-y (PMC10720230; doi:10.1186/s12916-023-03204-y)
Supplement: Supplementary file 2 — Additional file 2: Figure S1. The records selection flowchart of the Shaanxi, China dataset. Figure S2. The records selection flowchart of the UK-Biobank dataset. Figure S3. The selection flowchart of the multimorbidity networks for the overall inpatients among Chinese inpatients. Figure S4. The selection flowchart of the multimorbidity networks for male and female among Chinese inpatients. Figure S5. The selection flowchart of the multimorbidity networks for the overall among UK inpatients. Figure S6. The selection flowchart of the multimorbidity networks for male and female among UK inpatients. Figure S7.1. The number of unique comorbidity patterns related to each chapter (ICD-10, 1–14 chapters) in overall, male and female inpatients in China and the United-Kingdom (UK). Figure S7.2. The total frequency of comorbidities related to each chapter (ICD-10, 1–14 chapters) in overall, male and female inpatients in China and the United-Kingdom (UK). Figure S8. Property distribution for all nodes and hub nodes of the over, male and female inpatients among China and UK. Figure S9. The proportion trend of ICD-10 1–14 chapters by age. [file 12916_2023_3204_MOESM2_ESM.docx]

**Figure S1. The records selection flowchart of the Shannxi, China dataset.**


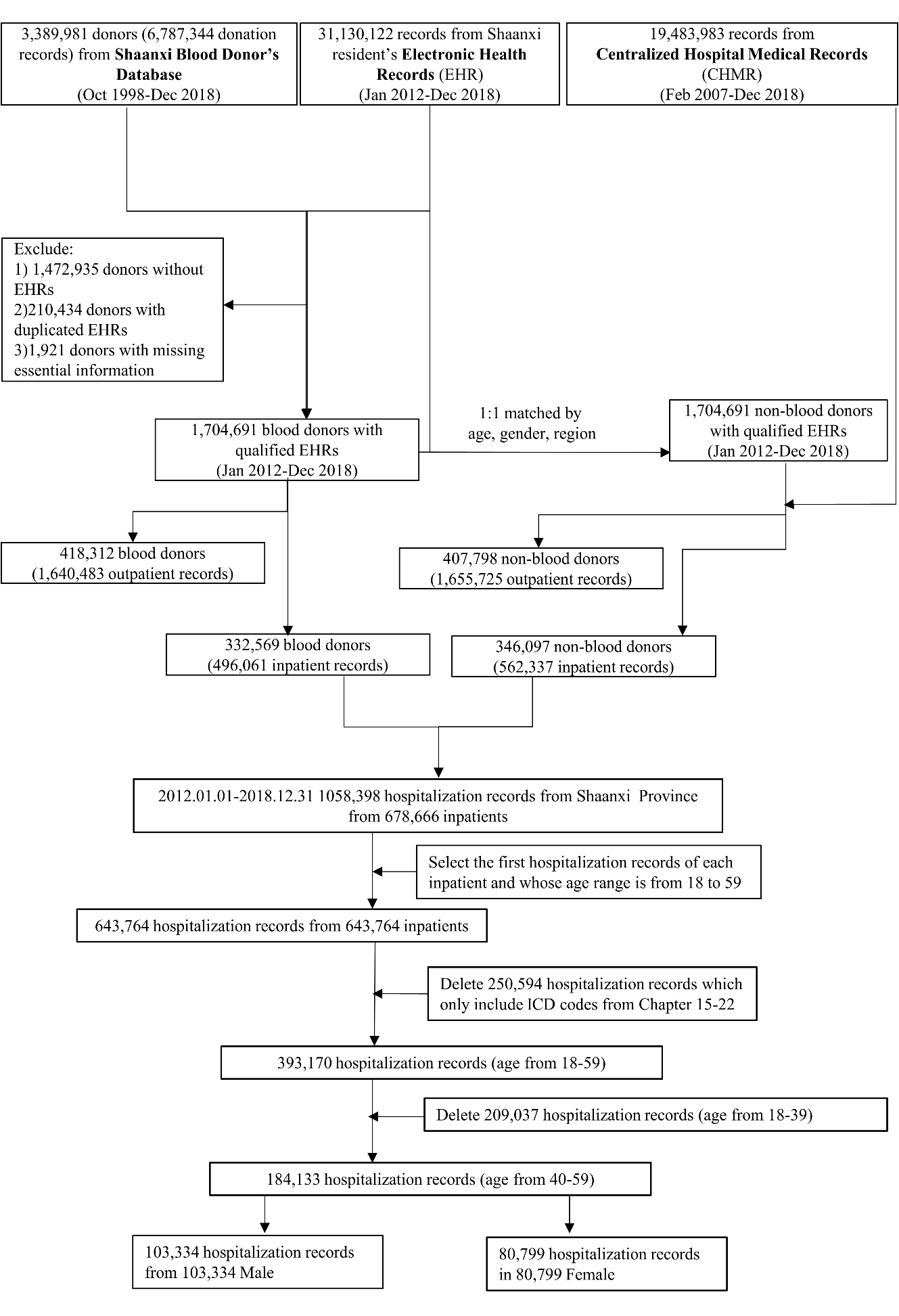


**Figure S2. The records selection flowchart of the UK-Biobank dataset.**


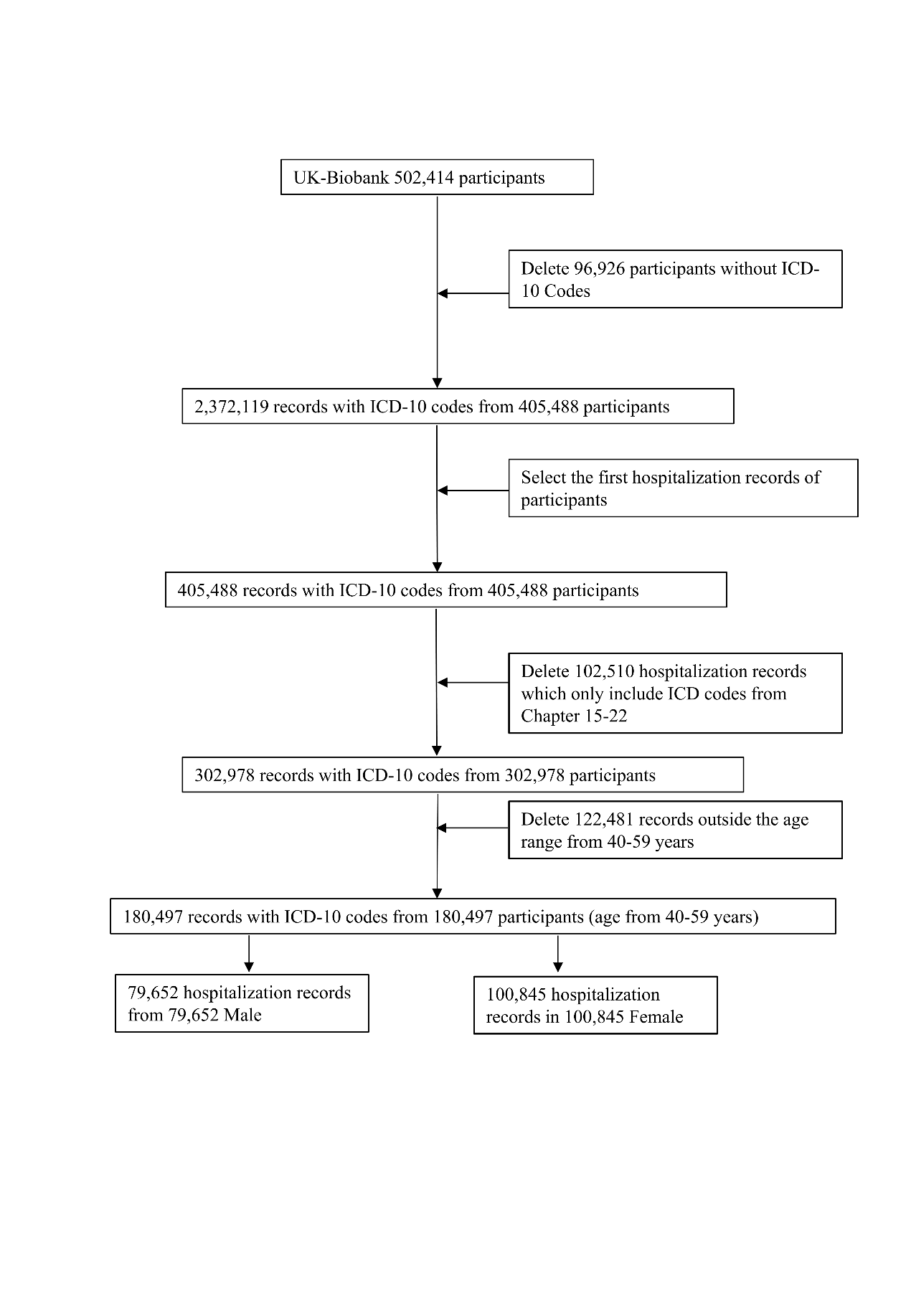


**Figure S3. The selection flowchart of the multimorbidity networks for the overall inpatients among Chinese inpatients.**


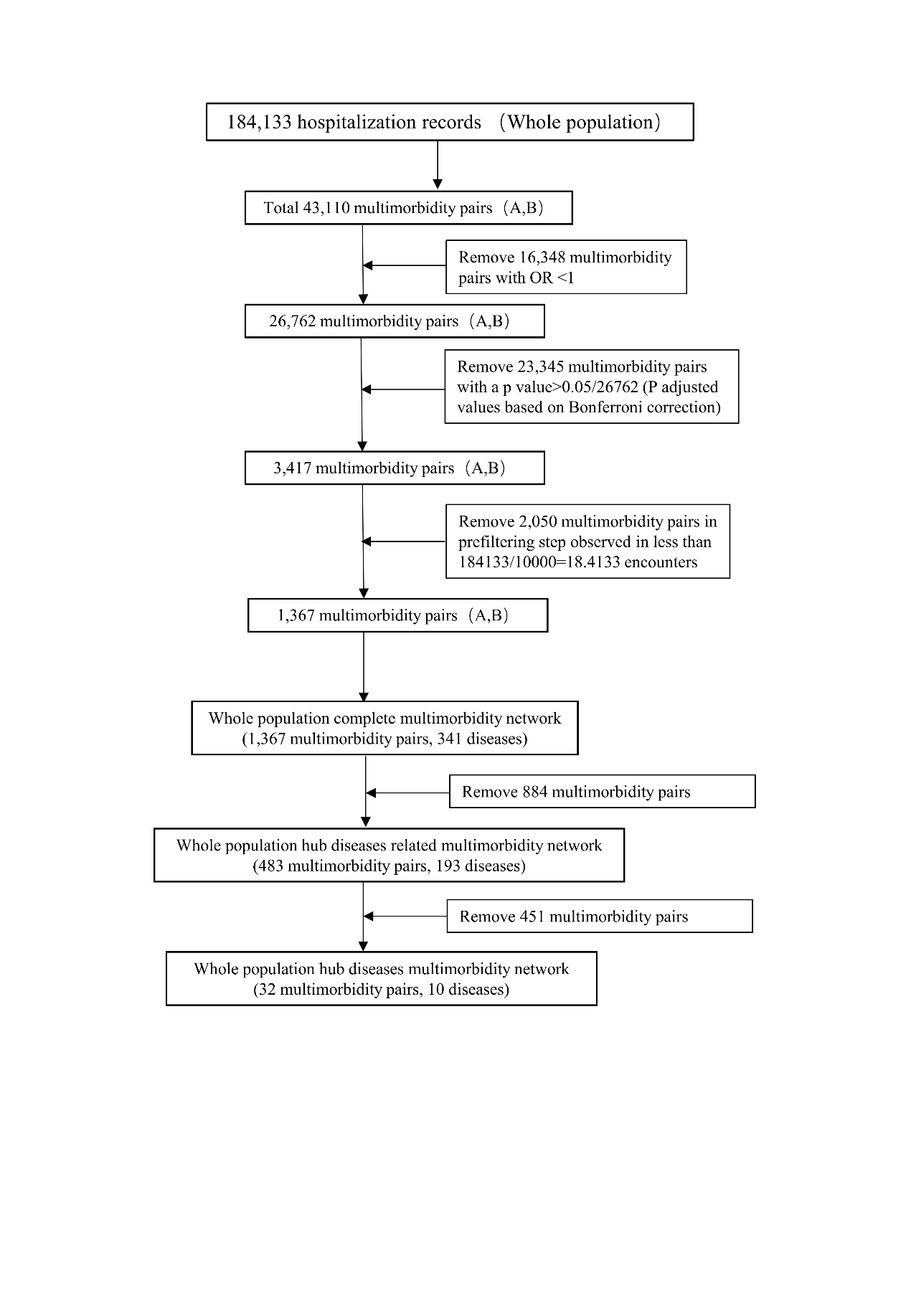


## multimorbidity pairs mean comorbidity patterns; hub diseases related network means hub diseases’ associated network

**Figure S4. The selection flowchart of the multimorbidity networks for male and female among Chinese inpatients.**

## multimorbidity pairs mean comorbidity patterns; hub diseases related network means hub diseases’ associated network

**Figure S5. The selection flowchart of the multimorbidity networks for the overall among UK inpatients.**

#multimorbidity pairs mean comorbidity patterns; hub diseases related network means hub diseases’ associated network

**Figure S6. The selection flowchart of the multimorbidity networks for male and female among UK inpatients.**


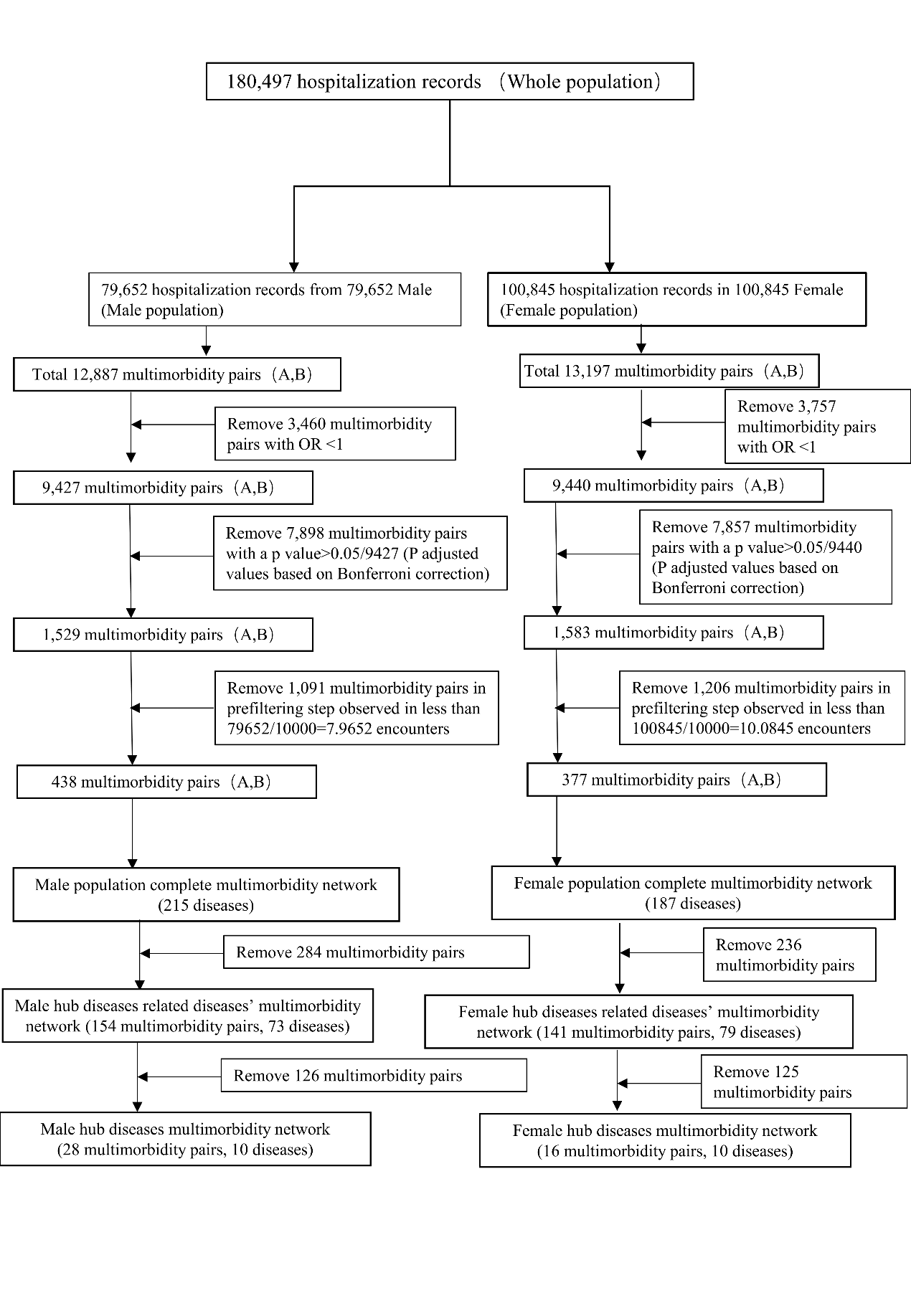
#multimorbidity pairs mean comorbidity patterns; hub diseases related network means hub diseases’ associated network

**Figure S7.1 The number of unique comorbidity patterns related to each Chapter (ICD-10, 1-14 Chapters) in overall, male and female inpatients in China and the United-Kingdom (UK).**

#C1: Certain infectious and parasitic diseases; C2: Neoplasms; C3: Diseases of the blood and blood-forming organs and certain disorders involving the immune mechanism; C4: Endocrine, nutritional and metabolic diseases; C5: Mental and behavioral disorders; C6: Diseases of the nervous system; C7: Diseases of the eye and adnexa; C8: Diseases of the ear and mastoid process; C9: Diseases of the circulatory system; C10: Diseases of the respiratory system; C11: Diseases of the digestive system; C12: Diseases of the skin and subcutaneous tissue; C13: Diseases of the musculoskeletal system and connective tissue; C14: Diseases of the genitourinary system.

**Figure S7.2 The total frequency of comorbidities related to each Chapter (ICD-10, 1-14 Chapters) in overall, male and female inpatients in China and the United-Kingdom (UK).**

# C1: Certain infectious and parasitic diseases; C2: Neoplasms; C3: Diseases of the blood and blood-forming organs and certain disorders involving the immune mechanism; C4: Endocrine, nutritional and metabolic diseases; C5: Mental and behavioral disorders; C6: Diseases of the nervous system; C7: Diseases of the eye and adnexa; C8: Diseases of the ear and mastoid process; C9: Diseases of the circulatory system; C10: Diseases of the respiratory system; C11: Diseases of the digestive system; C12: Diseases of the skin and subcutaneous tissue; C13: Diseases of the musculoskeletal system and connective tissue; C14: Diseases of the genitourinary system.

**Figure S8.** **Property distribution for all nodes and hub nodes of the over, male and female inpatients among China and UK.**

Two-sample Student’s (and Welch’s) t-test were used to judge whether the difference between the two means is significant (p<0.05 is significant).

*: The distributions were statistically significant. MCC: maximal clique centrality, Clo_Cen: closeness centrality, Clu_Coe: clustering coefficient, Bet_Cen: betweenness centrality.

FigureS8 a1-a3: The violin plots of the property distribution for all nodes and hub nodes of the over, male and female inpatients among China and UK.

FigureS8 b1-b3: The violin plots of the property distribution for all nodes of the over, male and female inpatients among China and UK.

FigureS8 c1-c3: The violin plots of the property distribution for hub nodes of the over, male and female inpatients among China and UK

**Figure S9. The** **proportion trend of ICD-10 1-14 chapters by age.**
